# Immune Markers and Risk of Pancreatic Cancer in the European EPIC Cohort

**DOI:** 10.1002/ijc.70581

**Published:** 2026-06-23

**Authors:** Verena A. Katzke, Yue Chen, Srimanti Dutta, Federico Canzian, Julie Louise Munk Andersen, Agnetha Linn Rostgaard‐Hansen, Léa Bouteille, Vinciane Rebours, Thérèse Truong, Matthias B. Schulze, Benedetta Bendinelli, Valeria Pala, Vittorio Simeon, Rosario Tumino, Carlotta Sacerdote, Roel Vermeulen, P. Martijn Kolijn, Sjoerd G. Elias, Marta Crous‐Bou, Maria‐José Sánchez, Ana Jimenez‐Zabala, José María Huerta, Marcela Guevara, Nick Wareham, Marie Breeur, Mattias Johansson, James Yarmolinsky, Daniele Campa, Rudolf Kaaks

**Affiliations:** ^1^ Division of Cancer Epidemiology German Cancer Research Center (DKFZ) Heidelberg Germany; ^2^ The Institute for Medical Information Processing, Biometry, and Epidemiology (IBE) Ludwig Maximilian University of Munich Munich Germany; ^3^ Genomic Epidemiology Group German Cancer Research Center (DKFZ) Heidelberg Germany; ^4^ Danish Cancer Institute Copenhagen Denmark; ^5^ Department of Public Health Aarhus University Aarhus Denmark; ^6^ Paris‐Saclay University, UVSQ, Inserm, Gustave Roussy, CESP Villejuif France; ^7^ Department of Pancreatology and Digestive Oncology, Beaujon Hospital, AP‐HP Paris‐Cité University Clichy France; ^8^ Department of Molecular Epidemiology German Institute of Human Nutrition Potsdam‐Rehbruecke Nuthetal Germany; ^9^ Institute of Nutritional Science University of Potsdam Nuthetal Germany; ^10^ Clinical Epidemiology Unit Institute for Cancer Research, Prevention and Clinical Network—ISPRO Florence Italy; ^11^ Epidemiology and Prevention Unit Fondazione IRCCS Istituto Nazionale Dei Tumori di Milano Milan Italy; ^12^ Medical Statistics Unit, University “L. Vanvitelli” Naples Italy; ^13^ Hyblean Association for Epidemiology Research, A.I.R.Ep. ETS Ragusa Italy; ^14^ Centre for Biostatistics, Epidemiology, and Public Health (C‐BEPH), Department of Clinical and Biological Sciences University of Turin Turin Italy; ^15^ Division of Environmental Epidemiology and Veterinary Public Health, Institute for Risk Assessment Sciences Utrecht University Utrecht the Netherlands; ^16^ Julius Global Health, the Julius Center for Health Sciences and Primary Care University Medical Center Utrecht the Netherlands; ^17^ Department of Epidemiology and Health Economics, Julius Center for Health Sciences and Primary Care University Medical Center Utrecht, Utrecht University Utrecht the Netherlands; ^18^ Unit of Nutrition and Cancer, Cancer Epidemiology Research Program, Catalan Institute of Oncology (ICO)—Bellvitge Biomedical Research Institute (IDIBELL), L'Hospitalet de Llobregat Barcelona Spain; ^19^ Department of Epidemiology Harvard T.H. Chan School of Public Health Boston Massachusetts USA; ^20^ Escuela Andaluza de Salud Pública (EASP) Granada Spain; ^21^ Instituto de Investigación Biosanitaria ibs.GRANADA Granada Spain; ^22^ Centro de Investigación Biomédica en Red de Epidemiología y Salud Pública (CIBERESP) Madrid Spain; ^23^ Biogipuzkoa Health Research Institute Group of Epidemiology of Chronic and Communicable Diseases San Sebastian Gipuzkoa Spain; ^24^ Ministry of Health of the Basque Government Sub‐Directorate for Public Health and Addictions of Gipuzkoa San Sebastian Gipuzkoa Spain; ^25^ Centro de Investigación Biomédica en Red de Epidemiología y Salud Pública (CIBERESP) Madrid Spain; ^26^ Department of Epidemiology Murcia Regional Health Council Murcia Spain; ^27^ Research Group on Epidemiology and Public Health Biomedical Research Institute of Murcia Pascual Parrilla (IMIB) Murcia Spain; ^28^ Instituto de Salud Pública y Laboral de Navarra Pamplona Spain; ^29^ Navarra Institute for Health Research (IdiSNA) Pamplona Spain; ^30^ MRC Epidemiology Unit, University of Cambridge School of Clinical Medicine Institute of Metabolic Science Cambridge UK; ^31^ Cancer Epidemiology Unit, Nuffield Department of Population Health University of Oxford Oxford UK; ^32^ Early Detection, Prevention, and Infections Branch (EPR) International Agency for Research on Cancer (IARC/WHO) Lyon France; ^33^ Imperial College London London UK; ^34^ Department of Biology University of Pisa Pisa Italy

**Keywords:** cohort, EPIC, immune markers, immune‐oncology, inflammation, Olink, proteins

## Abstract

The immune system is a major driver in pancreatic cancer development. Several prospective cohort studies have found associations for single immune system‐derived proteins such as IL6 or CRP, but results are inconclusive, and Omics‐based research is scarce. Hence, we aimed to investigate associations of a comprehensive protein panel with the risk of pancreatic cancer. Within the European Prospective Investigation into Cancer and Nutrition (EPIC) cohort, 92 immune proteins were measured in baseline blood samples of 406 incident pancreatic cancer cases and 406 sex‐ and age‐matched controls, using the Olink Immuno‐Oncology panel. Multivariable adjusted conditional logistic regression was used to estimate odds ratios (OR, 95% CI) for protein levels in association with pancreatic cancer risk. Eight biomarkers were associated with pancreatic cancer risk (MMP12, LAMP3, CD28, IL‐6, IL‐12, FASLG, PD‐L2, and PDCD1) but only MMP12 was significantly associated after multivariable adjustments for confounders and the seven proteins, with OR = 1.56 (95% CI: 1.20–2.03) for a doubling in protein concentration. After correction for multiple testing, none of the proteins were associated with risk. Restricting analyses to cases diagnosed within the first 4 years and 4–8 years after recruitment resulted in OR of 1.89 (95% CI: 1.28–2.80) and 1.37 (95% CI: 1.01–1.86) for MMP12, respectively. Higher levels of MMP12 were associated with pancreatic cancer risk specifically in those diagnosed shortly after recruitment, while other immune‐related factors were not associated with risk. Further cohort studies are needed to confirm our initial findings.

## Abstract (highlights)

What's new?

The immune system has been implicated as a major driver in pancreatic cancer etiology. Previous cohort studies have found associations between pancreatic cancer and single immune system‐derived markers, but omics‐based evidence remains scarce. In this prospective case–control study, the authors investigated 92 proteins involved in immune reactions and inflammation using the Olink Immuno‐Oncology panel. Higher levels of MMP12 were associated with pancreatic cancer risk specifically among patients diagnosed shortly after recruitment, while other immune‐related factors were not associated with risk. MMP12 might be an early sign of immune disturbances up to 8 years preceding a pancreatic cancer diagnosis.

## Graphical abstract

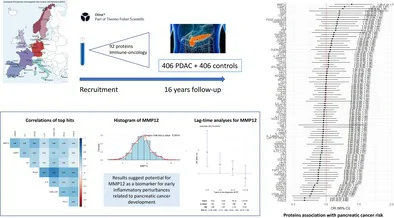

**Abbreviations**
AUC: area under the curve
BH‐FDR: Benjamini–Hochberg False Discovery Rate
BMI: body mass index
CAFs: cancer‐associated fibroblasts
CAR‐T: chimeric antigen receptor T cell
CKB: China Kadoorie Biobank
ECM: extracellular matrix
EPIC: European Prospective Investigation into Cancer and Nutrition
HR: hazard ratio
IARC: International Agency for Research on Cancer
ICD: International Classification of Diseases
IPMN: intraductal papillary mucinous neoplasm
LASSO: Least Absolute Shrinkage and Selection Operator
LOD: limit of detection
MCs: macrophages
NPX: Normalized Protein Expression
OR: odds ratios
PDAC: Pancreatic ductal adenocarcinoma
PEA: Proximity Extension Assay
SAS 9.4: Statistical Analysis System
TME: tumor microenvironment
UKB: UK Biobank

## 1 Introduction

Pancreatic ductal adenocarcinoma (PDAC) is one of the most aggressive and deadly malignancies with a 5‐year survival after diagnosis that approaches 12% [1, 2]. Research over the past few decades has shown a strong role of inflammation in PDAC etiology [3, 4], with both genetic and environmental factors potentially contributing to the development of inflammation. Moreover, several predisposing conditions such as chronic pancreatitis, pre‐existing type 2 diabetes, and intraductal papillary mucinous neoplasm (IPMN) also have a strong inflammatory component [5, 6, 7]. While the etiology of PDAC has yet to be fully clarified, a limited number of putative risk factors have been identified, such as being male, cigarette smoking, obesity, hyperglycemia, diabetes, age, family history of pancreatic cancer, and around 30 germline common genetic variants [8, 9, 10, 11, 12].

At the molecular level, when the pancreas develops precancerous lesions or progresses to an invasive tumor, cancer cells interact with various immune (e.g., T cells, neutrophils, macrophages) and stromal cells (cancer‐associated fibroblasts [CAFs]) to form the tumor microenvironment (TME). CAFs secrete large amounts of proteins that form a dense extracellular matrix (ECM) around tumor cells. Immune cells, through complex regulatory mechanisms, can exert both immune and immunosuppressive effects, helping tumor cells escape immune surveillance and elimination. Although the exact mechanisms of immune response are not fully understood, proteins actively secreted by cells or passively leaked into the bloodstream are often associated with disease onset and progression of pancreatic cancer [13, 14, 15, 16]. The analysis of immune proteins in blood may provide insights into understanding the etiology and status of PDAC.

Multi‐marker proteomic panels have become of great interest given the simultaneous measurement of many peptide analytes in one run and the requirement of small volumes. In a series of clinical studies, a Danish research group applied the Olink immune‐oncology target panel of 92 selected candidate proteins to identify new predictive, diagnostic or prognostic biomarkers or indices for PDAC, with inconsistent results [17, 18, 19, 20, 21]. For example, proteins retained in indices varied in predictive versus prognostic models and those applicable for diagnostic use were not useful to assess resectability. Population‐based epidemiological investigations applying the same panel are limited to a prospective case‐cohort study in China that reported associations of several immune‐system related proteins with risk of PDAC [22] and a more recent investigation in the UK Biobank; however, observing little evidence [23].

To further explore the potential role of immune‐related proteins in the etiology of pancreatic cancer, we conducted a case–control study nested within the European Prospective Investigation into Cancer and Nutrition (EPIC) cohort on 406 case–control pairs and applied the Olink immune‐oncology panel. The immuno‐oncology panel was chosen because of the known associations between inflammation and the development of pancreatic cancer, and because PDAC development is known to be related to an immunosuppressive microenvironment [24].

## 2 Materials and Methods

### 2.1 Study Population

The EPIC cohort was set up to investigate etiological associations of environmental and behavioral factors with chronic disease risks. Between 1992 and 2000 approximately 520,000 participants aged 35–70 years were recruited through 23 collaborating centers in 10 European countries (Denmark, France, Germany, Greece, Italy, the Netherlands, Norway, Spain, Sweden, and the United Kingdom). Participants were asked to fill out questionnaires capturing sociodemographic information (e.g., level of education), lifestyle (e.g., smoking history), nutrition (e.g., alcohol consumption), and disease history (e.g., diabetes). Body weight, height, waist and hip circumference were measured in most centers by trained personnel and the body mass index was derived thereof (BMI, kg/m^2^). Seventy‐four percent of the participants also provided blood samples, which were processed into serum, plasma, red blood cells, and buffy coat containing all nucleated cells, and then stored in liquid nitrogen at −190°C at the International Agency for Research on Cancer (IARC) and at the respective recruitment centers. Incident cancers are identified through a combination of methods including linkage to population cancer registries (Denmark, Italy, the Netherlands, Spain, Sweden, and the United Kingdom) and active follow‐up methods by means of contacting participants or their next‐of‐kin (France, Germany, Greece). Mortality information was collected through population registries (all countries), combined with national health insurance data in France [25, 26].

### 2.2 Nested Case–Control Design

Incident invasive exocrine pancreatic cancer cases (ICD‐10 code C25, 25.0–25.3, 25.7–25.9) diagnosed between 1995 and 2012 were sampled into a nested case–control study. Cases with prior diagnosis of other malignant tumors (except for non‐melanoma skin cancer) or with unavailable blood samples were excluded. For each case, a control person was selected using an incidence density sampling protocol, using study recruitment center, sex, age (±6 months), date (±2 months) and time of blood collection (±2 h), sample specimen (serum or plasma) and fasting status (< 3, 3–6, ≥ 6 h) as matching factors. After excluding internal quality control samples, those without a matched partner and those with completely missing biomarker measurements, a total of 406 matched case–control pairs remained for statistical analyses (Figure S1).

### 2.3 Laboratory Methods

In this study, 92 proteins from the Olink immuno‐oncology panel were measured, using the Proximity Extension Assay (PEA) technology (details on the method in references [27, 28] and names of the proteins in Table S1). Briefly, real‐time quantitative PCR is used to measure relative changes in protein expression. Those are then translated into a relative quantification unit (Normalized Protein eXpression, NPX) using a series of computations, including correction for inter‐plate measurement shifts and muting of background noise. The panel assays were conducted at an Olink‐certified laboratory at Helmholtz Zentrum München, Neuherberg, Germany. Serum and plasma samples were analyzed in batches, sorted by EPIC center, and paired case–control samples were placed in the same batch in random and blinded order. Laboratory personnel remained blinded to the case–control status of the samples. Plasma and serum samples were run separately after re‐calibration of the method to account for differences in biospecimen. Results were reported as NPX values, representing the relative protein expression levels on a log2 scale. High NPX value corresponds to a high protein concentration. Protein measurements below the limit of detection (LOD) were replaced with the corresponding LOD value. For each protein, distribution of missing and LOD values is depicted in Figure S2 and histogram and density are depicted in Figure S3.

### 2.4 Statistical Analysis

For 110 individuals with up to five missing NPX values, multiple imputation was used to fill in the missing data; no individual exceeded five missings (*N* = 94 individuals have one missing value and 16 have up to five missing values; no differences by sample type and case–control status). Generalized McNemar's tests were used to assess differences of categorical baseline characteristics in matched case–control pairs, while Wilcoxon signed‐rank tests were used to compare continuous variables between the paired groups, as these variables were not normally distributed. Associations of proteins with selected baseline characteristics (as potential confounders) in controls were investigated applying Spearman correlation for continuous and Chi^2^ tests for categorical variables, adjusted for batch, country, age at recruitment and sex. Conditional logistic regression was used to derive odds ratios (OR) and 95% confidence intervals (CI) for associations of continuous (log2‐scale) protein levels with pancreatic cancer risk. Minimally adjusted (for matching factors) and multivariable adjusted analyses were performed, selecting as adjustment factors variables that are known to be associated with PDAC risk and/or variables that showed an association with pancreatic risk in this dataset. Specifically, BMI (continuous), smoking (never, former, current, missing), diabetes (yes, no, unknown, missing) and alcohol consumption (categories, including missing) were included in the final model. Level of education (a proxy for socio‐economic status), body height, and physical activity were univariably not associated with pancreatic cancer risk in our setting and, therefore, were excluded from the analyses. Stratification by biospecimen did not alter risks and results were, therefore, shown for plasma and serum combined but models were indirectly adjusted for by using batch.

First conditional logistic regressions including one biomarker at a time were carried out to identify immune proteins significantly associated with PDAC risk. Next, these biomarkers were included in a multivariable model and finally, a multinomial regression model was used to conduct subgroup analyses based on lag‐time to pancreatic cancer diagnosis (0–4, 4–8, 8–12, > 12 years). A secondary screening of the 92 biomarkers was performed using the Least Absolute Shrinkage and Selection Operator (LASSO) regression to limit overfitting through variable shrinkage and selection. To this effect, the 92 proteins and selected confounders (batch, age at recruitment, sex, country, BMI, smoking status, diabetes, alcohol consumption) were included in the LASSO regression. LASSO applies an L1 penalty to the regression coefficients; the penalty parameter λ was determined via 10‐fold cross‐validation. At the optimal λ, the model retained non‐zero protein biomarkers, and their log‐odds coefficients were exponentiated to obtain odds ratios. To enhance the robustness of variable selection, stability selection based on repeated subsampling was further applied, retaining variables with selection probabilities > 0.75 while controlling the per‐family error rate (PFER) at 1.

We corrected for multiple testing applying the Benjamini–Hochberg False Discovery Rate (BH‐FDR) method. Statistical analyses were performed in SAS 9.4 (Statistical Analysis System, RRID:SCR_008567) and R version 4.4.0 (R Project for Statistical Computing, RRID:SCR_001905). Packages in R include Mice, Stats, Survival and Glmnet. All statistical tests are two‐sided with significance level α = 0.05.

## 3 Results

### 3.1 Basic Characteristics

The median age at pancreatic cancer diagnosis was 65 years after a median follow‐up time of 8.6 years. The majority were reported to be situated in the head of the pancreas (*N* = 167, 41%), 10% in the pancreas' body and 8% in the tail, while no tumor sub‐localization was specified for 166 cases (40%). Seventy percent of pancreatic cancer cases (*N* = 284) were microscopically confirmed (histological and/or microscopical examination or autopsy). Stage and grade information was largely missing (62% and 79%, respectively). The proportion of current smokers was higher in cases (33%) compared to controls (23%, *p* = 0.005). About 90% of participants reported being alcohol consumers at time of recruitment into the EPIC study, and almost 50% of participants reported to be physically active, with no apparent differences for either characteristic by case control status. Baseline BMI was marginally higher in cases (median 25.9 kg/m^2^) compared to controls (25.5 kg/m^2^, *p* = 0.04); and cases reported more often having diabetes than controls (5.2 vs. 3.5%, *p* = 0.23, Table 1).

**TABLE 1:** Baseline characteristics of pancreatic cancer cases and matched controls in the EPIC cohort.

Variables (median (IQR) or *n* (%))	Cases (*n* = 406)	Controls (*n* = 406)	*p* *
Age at recruitment, years	57 (52.2–60.9)	57 (52.2–61.0)	Matched
Age at diagnosis, years	65 (60.2–69.7)	—	
Time from recruitment to PC diagnosis, years	8.6 (5.9–10.4)	—	
Sex			Matched
Male	194 (48%)	194 (48%)	
Female	212 (52%)	212 (52%)	
Smoking status			**0.005**
Never smoker	148 (36%)	178 (44%)	
Former smoker	124 (31%)	134 (33%)	
Current smoker	134 (33%)	94 (23%)	
Alcohol intake at recruitment, g/day			0.99
0	40 (10%)	42 (10%)	
> 0–6 (men)/> 0–3 (women)	118 (29%)	120 (30%)	
> 6–12 (men)/> 3–12 (women)	88 (22%)	85 (21%)	
> 12–24	63 (15%)	66 (16%)	
> 24	97 (24%)	93 (23%)	
Physical activity index			0.95
Inactive	66 (16%)	63 (16%)	
Moderately inactive	99 (24%)	107 (26%)	
Moderately active	164 (40%)	160 (39%)	
Active	28 (7%)	28 (7%)	
Missing	49 (12%)	48 (12%)	
Educational level			0.34
Nearly Illiterate/unknown	24 (6%)	27 (7%)	
Primary school	152 (37%)	127 (31%)	
Technical/professional school	103 (25%)	124 (31%)	
Secondary school	52 (13%)	50 (12%)	
Higher education (university degree or above)	75 (19%)	78 (19%)	
BMI, kg/m^2^	25.9 (24–29)	25.5 (23–28)	**0.04**
Diabetes, self‐reported			0.23
Yes	21 (5.2%)	14 (3.5%)	
No	337 (83%)	348 (86%)	
Unknown	48 (12%)	44 (11%)	

*Note:* Controls were matched to cases by age at recruitment; sex; country; laboratory batch. Bold values indicated statistical significance (*P* < 0.05).

* Generalized McNemar's tests for categorical variables, Wilcoxon signed‐rank tests for continuous variables.

### 3.2 Association of Proteins With Each Other and With Potential Confounding Factors

Among controls PDGF.subunit.B, KIR3DL1, and MIC.A.B were negatively correlated with almost all other proteins of the immune‐oncology panel, whereas more than 40 biomarkers, including PD.L1, TNFRSF9, and CD5, showed moderate to high positive correlations with at least half of the other biomarkers (Figure S4). Hierarchical clustering did not reveal any prominent cluster but PDGF.subunit.B appeared to be the protein most consistently and strongly correlated with almost all other proteins. Twenty biomarkers were weakly positively correlated with age at recruitment, while MUC.16 showed a weak negative correlation of r = −0.14 (Figure S5). Eight biomarkers showed a weak positive correlation with BMI (*r* = 0.10–0.23), while six biomarkers showed a weak negative correlation (r = −0.16 to r = −0.11) (Figure S6). Twenty‐three out of the 92 biomarkers were significantly associated with sex and among these, the blood levels of 19 biomarkers were higher in women than men (Figure S7). Twenty‐five biomarkers were significantly associated with smoking status and of these, eight biomarkers showed a progressive increase in levels from never to former and to current smokers, while IL12 levels were lower in current smokers compared to former or never smokers (Figure S8). Ten biomarkers were significantly associated with alcohol consumption; however, none of them exhibited a consistent trend with higher alcohol intake (Figure S9). Four biomarkers (ADGRG1, ANGPT2, CXCL1, CCL19) were significantly associated with diabetes, with higher levels observed in diabetic participants (Figure S10).

### 3.3 Individual Biomarkers and Risk of Pancreatic Cancer

**FIGURE 1:**
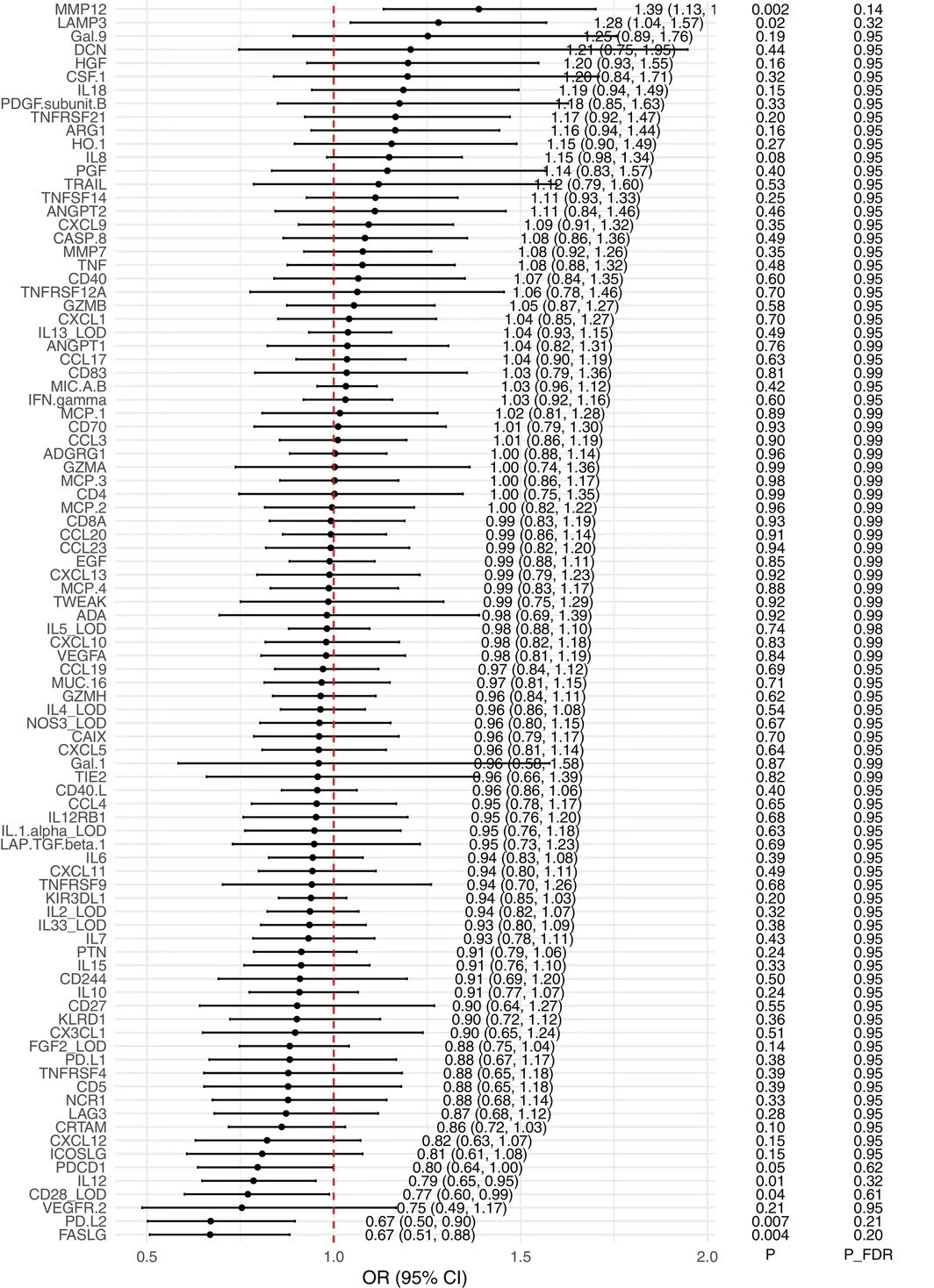
Forest plot of biomarkers association with pancreatic cancer risk, crude. Adjusted for matching factors (age at recruitment, sex, center, laboratory batch). Both crude *p* values (P) and false discovery rate–adjusted *p* values (P_FDR) are reported.

**FIGURE 2:**
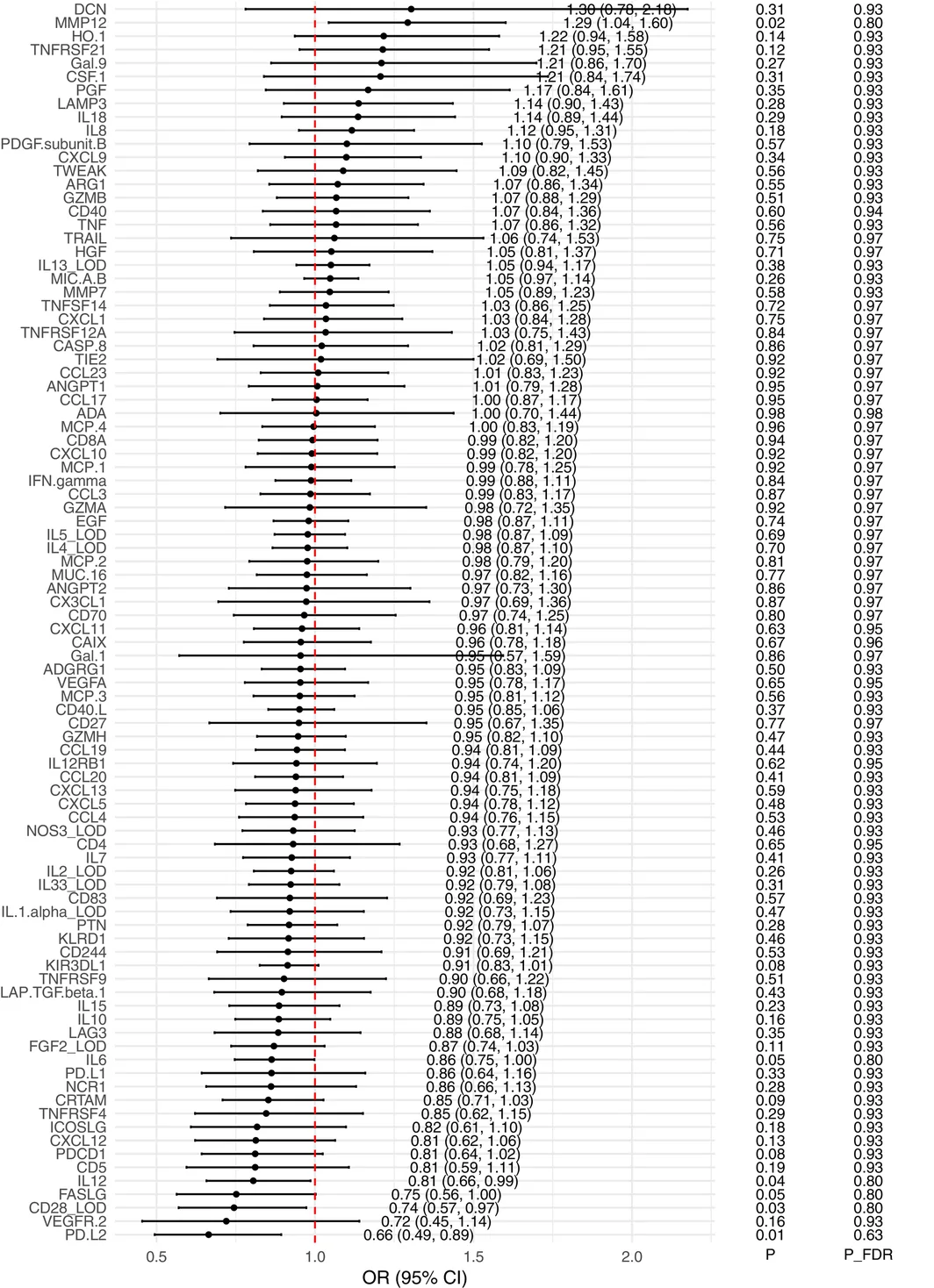
Forest plot of biomarkers associations with pancreatic cancer risk, adjusted. Adjusted for matching factors and smoking status, BMI, diabetes and alcohol at recruitment. Both crude *p* values (P) and false discovery rate–adjusted *p* values (P_FDR) are reported.

In minimally, by matching factors only, adjusted conditional logistic regression models, higher levels of MMP12 (OR = 1.39 [95% CI: 1.13–1.70]) and LAMP3 (OR = 1.28 [1.04–1.57]) were associated with increased pancreatic cancer risk. In contrast, higher levels of IL12 (OR = 0.79 [0.65–0.95]), CD28 (OR = 0.77 [0.60–0.99]), PD‐L2 (OR = 0.67 [0.50–0.90]), FASLG (OR = 0.67 [0.51–0.88]), and of PDCD1 (OR = 0.80 [0.64–1.00]) were associated with lower pancreatic cancer risk (Figure 1). After additional adjustments for smoking status, alcohol consumption, self‐reported diabetes and BMI, only MMP12 (OR = 1.29 [1.04–1.60]) remained associated with increased pancreatic cancer risk, whereas significant inverse associations remained for IL12 (OR = 0.81 [0.66–0.99]), CD28 (OR = 0.74 [0.57–0.97]), PD‐L2 (OR = 0.66 [0.49–0.89]), and IL6 (OR = 0.86 [0.75–100]) (Figure 2). Further analysis showed positive pairwise correlations among all of these eight biomarkers, with Spearman correlation coefficients from *r* = 0.11 to *r* = 0.66 (Figure S11). After correction for multiple testing, none of the proteins were significantly associated with pancreatic cancer risk in the multivariable adjusted model.

Multivariable logistic regression models that included all 8 biomarkers (MMP12, LAMP3, IL12, CD28, PD‐L2, FASLG, PCDC1, and IL6), additionally adjusting for smoking status, alcohol consumption, self‐reported diabetes, and BMI, showed a significant (*p* < 0.05) association of PDAC risk only for MMP12 (OR = 1.56 [1.20–2.03]) and IL6 (OR = 0.84 [0.71–0.99]) (Table 2). By contrast, a LASSO regression approach for all 92 proteins showed significant associations for MMP12, CD28, FASLG, PD‐L2, and IL12, all of which were also selected in the initial single‐marker model analyses.

**TABLE 2:** Associations of selected biomarkers with risk of pancreatic cancer, multivariable and mutually adjusted.

Variable	OR	95% CI	*p*
MMP12^a^ (enzyme)	**1.56**	**1.20–2.03**	**0.001**
LAMP3 (membrane protein)	1.22	0.93–1.61	0.15
IL6 (interleukin)	**0.84**	**0.71–0.99**	0.05
PDCD1 (membrane protein)	1.02	0.71–1.44	0.93
IL12^a^ (interleukin)	0.81	0.64–1.04	0.09
CD28^a^ (receptor protein)	0.82	0.58–1.16	0.25
PD‐L2^a^ (membrane protein)	0.71	0.50–1.01	0.05
FASLG^a^ (transmembrane protein)	0.78	0.56–1.09	0.15

*Note:* OR for a doubling in concentration. Controls matched to cases by age at recruitment, sex, center, laboratory batch. Multivariable adjusted by smoking status, alcohol consumption, self‐reported diabetes, BMI and mutually for the eight biomarkers. Bold values indicated statistical significance (*P* < 0.05).

^a^ Validated through LASSO.

### 3.4 Subgroup Analysis by Lag‐Time of Pancreatic Cancer Diagnosis

**FIGURE 3:**
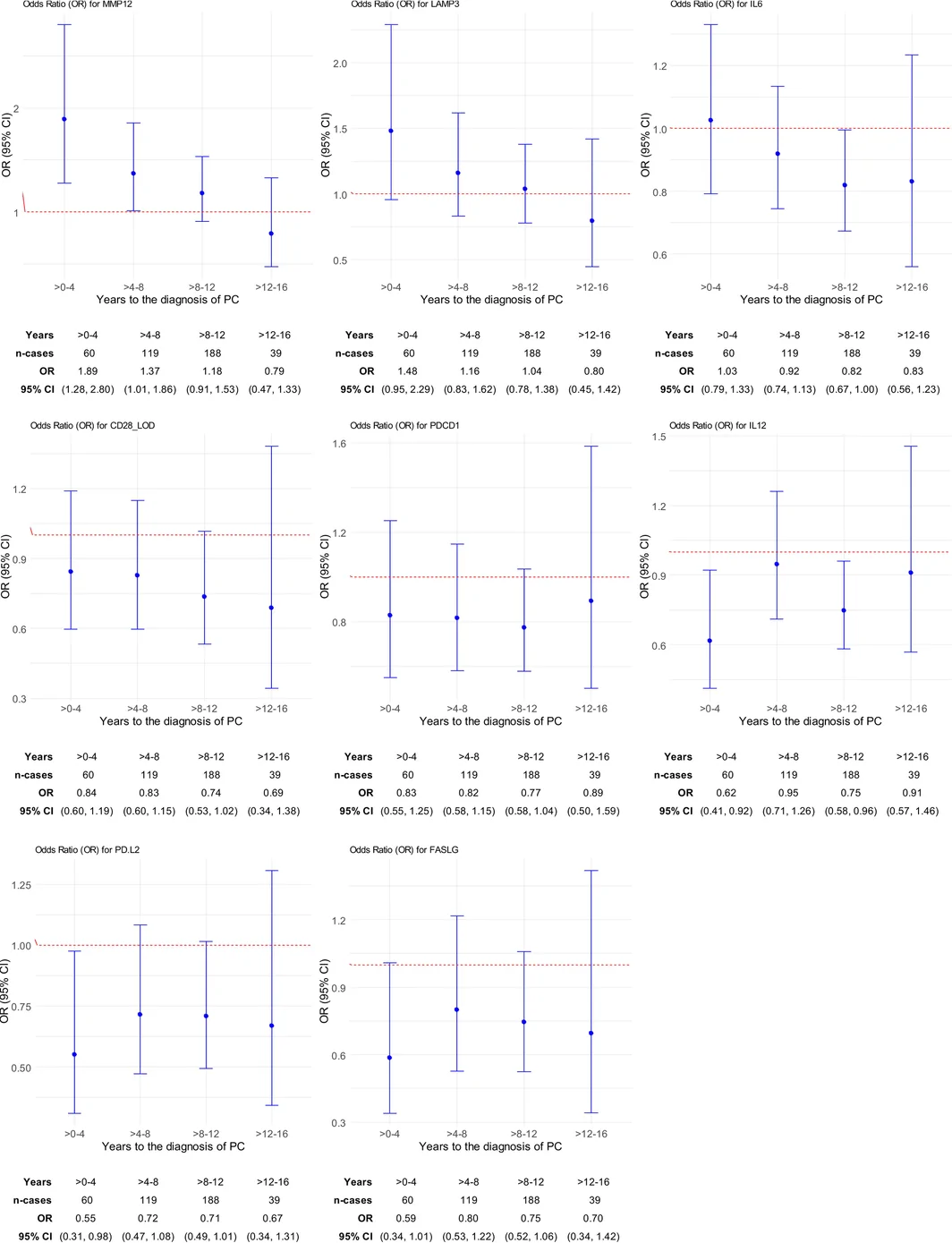
Associations of biomarkers with pancreatic cancer risk by lag time, multivariable adjusted. Adjusted for matching factors (age at recruitment, sex, country, laboratory batch) and smoking status, BMI, diabetes and alcohol at recruitment.

Analyses stratified by 4‐year lag‐time intervals between blood donation and PDAC diagnosis showed ORs with gradually smaller effects sizes for MMP12 and LAMP3 as lag‐times became longer, and ORs < 1 within the last interval of > 12–16 years. The inverse associations for IL6 and CD28 gradually became stronger with longer lag‐times (i.e., drop even further). The effect estimates for CD28 and PDCD1 remain largely unchanged in this analysis. However, only for MMP12 was the OR statistically significant (*p* < 0.05), for the > 0–4 (OR = 1.89 [1.28–2.80]) and > 4–8‐year (OR = 1.37 [1.01–1.86]) lag‐time intervals (Figure 3).

## 4 Discussion

This study investigated the association between 92 proteins involved in immune reactions and inflammation and pancreatic cancer risk within the EPIC cohort. In single‐marker analyses, eight biomarkers showed significant or borderline significant associations with pancreatic cancer risk: MMP12 and LAMP3 were associated with higher risks, whereas CD28, IL6, IL12, PD‐L2, FASLG, and PDCD1 were associated with lower risks. When these eight biomarkers were mutually adjusted in one regression model, a significant association remained for MMP12 and IL‐6. Subgroup analysis based on lag‐time until pancreatic cancer diagnosis showed a higher risk of pancreatic cancer for high MMP12 levels, but only considering a lag‐time of less than 8 years between blood collection (time of marker analysis) and diagnosis.

Utilizing comparable Olink platforms, two studies in prospective cohorts investigated the associations of proteins with risk of incident pancreatic cancer. Using the same Olink immune‐oncology panel, a study nested within the China Kadoorie Biobank (CKB, 610 prospectively diagnosed pancreatic cancer cases plus a random sub‐cohort of 623 study participants) showed increased risk associations of pancreatic cancer with MMP12 (1.74 [1.32–2.29]), in line with our observations. However, the associations in the CKB study were generally limited to risk of PDAC diagnosed within the first year of follow‐up, potentially reflecting reverse causation and not aetiological associations as in our study. Contrasting observations, however, were IL6 (1.85 [1.33–2.04]), PDCD1 (1.21 [1.04–1.41]), and PD‐L2 (1.43 [1.04–1.97]) being associated with higher pancreatic cancer risk in the CKB study, whereas we found a potential inverse association. Furthermore, the CKB study showed associations of several other proteins (e.g., ANGPT2, IL18, CD4, HGF) with PDAC risk that were not confirmed by our analyses. Potential explanations for the inconsistent results could be represented by differences in blood processing protocols, differences related to geographical region and ethnicity, slightly different median ages at cancer diagnosis (60 vs. 65 in our study), or diverging lag‐times (< 1 year in CKB vs. up to 12 years in EPIC).

A recent investigation in the UK Biobank (UKB) project utilizing 44,645 participants and 4921 incident cancer cases (including 106 pancreatic cancer cases) measured 1463 proteins using the Olink platform. Papier et al. [23] did not observe any significant associations with pancreatic cancer risk but MMP12 was associated with an increased risk of cancers of the colon, lung, and NHL (pancreas HR = 1.18 (0.95–1.45)). Discrepancy in results might be due to the extensive corrections for multiple testing applied in the UKB given the almost 1500 proteins, limited number of pancreatic cancer cases (106 vs. 406 in our study), and slightly older age at diagnosis (70 vs. 65 years in our study). Partly deviating results of both cohorts from ours call for further prospective studies to validate either finding.

Besides the CKB and UKB studies, a limited number of case–control and clinical studies have applied the Olink immune‐oncology target panel to describe expression levels in PDAC patients (stage I to IV), patients with chronic pancreatitis, and healthy individuals to identify potentially predictive, diagnostic and/or prognostic biomarkers for pancreatic cancer [17, 18, 19, 20, 21]. For example, MMP12 levels were higher and PD‐L2 lower in 701 predominantly late‐staged pancreatic cancer patients compared to 180 controls in a Danish case–control study [17], in line with risk directions in our study. Further, two small clinical studies reported higher MMP12 serum levels in PDAC patients (*N* = 25 and 51) compared to healthy controls (*N* = 25 and 44) [29, 30]. The latter study by Kahlert et al. [30] also reported perfect discriminatory capability of MMP12 for PDAC patients versus healthy control donors, with AUC = 1% and 100% sensitivity and specificity after correction for multiple testing. And in a clinical study on 363 patients with advanced PDAC both MMP12 and IL6 presented with higher levels in patients with shorter (< 90 days) compared to longer survival (> 2 years) [21].

Evidently, MMP12 levels are elevated in pancreatic cancer patients, most likely as an immune response to the tumor. It appears, however, that higher MMP12 is potentially associated with higher pancreatic cancer risk in prospective studies, and that the associations are stronger when the measurements were made closer to PDAC diagnosis, and risk inverts with longer lead‐times. Potentially, individuals with higher life‐long levels (in an attempt to reduce inflammatory response) may be less likely to develop the disease whereas high acute‐levels more recent to disease diagnosis could be indicative of an immune response to a subclinical disease. Thus, our results suggest that MMP12 could be involved in PDAC etiology, and could be potentially useful as an early biomarker in pre‐disease progression monitoring. In that sense, measuring MMP12 levels over the course of PDAC development would be extremely valuable.

MMP are associated with tumorigenesis and are key modulators of the TME [31]. MMP12, the protein that showed the most consistent association in our study, is synthesized and released by tumor‐stroma‐associated cells such as macrophages (MCs) and can effectively degrade protein components of the ECM. It is also involved in angiogenesis and the regulation of various cytokines (such as TNF‐α and IL‐1), growth factors, and cell surface ligands and receptors [32, 33]. The loss of ECM homeostasis and integrity is considered one of the hallmarks of cancer, evidently also for pancreatic cancer [33].

In addition to MMP12, a limited number of further proteins from the Olink immune‐oncology panel were found to be associated with the risk of pancreatic cancer in our study. However, their potential biological roles in tumorigenesis only partly explain our observations.

The expression of CD28 and PD‐L2—proteins that showed a potential inverse association with PDAC risk, has been closely related to T cell‐driven immune responses, thereby exerting either tumor suppressive (CD28) or tumor promoting (PD‐L2) [34, 35, 36, 37, 38] effects. The anti‐pancreatic tumor effect of CD28 has been widely studied in biological experiments and has become a popular topic in research on chimeric antigen receptor T cell (CAR‐T) therapy for pancreatic cancer [39]. The immunosuppressive roles of PDCD1 and its ligand PD‐L2 have been validated in numerous biological experiments [40] and have led to the development of related immunotherapies. Contrary to mechanistic studies and clinical evidence, PD‐L2 was inversely associated with pancreatic cancer risk in our study, albeit not statistically significant in all our models.

IL6 and IL12 are two interleukins that showed an inverse association with PDAC risk. IL12 is primarily responsible for inducing and enhancing immune responses, promoting cancer cell apoptosis, and inhibiting angiogenesis [41, 42]. IL6 stimulates tumor cell proliferation, survival, and angiogenesis by promoting the secretion of growth, survival, and pro‐angiogenic factors. It can also act on a wide range of immune cells, reducing immune responses while upregulating the activity of regulatory T cells, for example [43]. Okada and colleagues [44] found that IL6 expression increased significantly as pancreatic cancer progressed to late stages. Within the EPIC cohort, using another assay, we previously found a non‐significant increased risk for PDAC risk with increasing IL6 levels [45], contradictory to our present findings, whereas no association between plasma IL6 and risk of pancreatic cancer was found in a prospective study of five US cohorts [46]. However, obtained results are limited to few studies, laboratory methods deviate greatly, and comparisons of results should be treated with caution.

Our nested investigation within the prospective EPIC cohort has some potential limitations. For example, pancreatic cancer staging and grading at time of diagnosis is largely unknown in EPIC, and therefore it was not possible to carry out subgroup analyses considering this. Further, results derived from proteins with a large proportion of measurements below the detection limit are less reliable and should be interpreted with caution, such as CD28 with 35% below LOD. Validation to counteract data overfitting was not possible in our study and needs to be addressed in future investigations. With a sample size of 406 case‐sets and 92 potential biomarkers, associations with pancreatic cancer may have been overlooked whereas the associations observed might present chance findings. However, results for MMP12 are robust across lag‐time analyses and after adjustments for confounders and other proteins of the investigated immune panel.

In conclusion, our prospective study identified particularly MMP12 as being associated with pancreatic cancer risk. This association is more pronounced in short‐to‐median lag‐times (up to 8 years) between measurement and cancer diagnosis, suggesting potential for MMP12 as a biomarker for early inflammatory perturbances related to pancreatic cancer development. Our results are not fully in agreement with two previous investigations and, therefore, further cohort studies are needed to confirm our findings.

## Author Contributions

**Verena A. Katzke:** conceptualization, methodology, data curation, investigation, validation, supervision, visualization, project administration, resources, writing – original draft, writing – review and editing. **Yue Chen:** methodology, visualization, validation, formal analysis, writing – original draft, writing – review and editing. **Srimanti Dutta:** methodology, formal analysis, validation. **Federico Canzian:** writing – review and editing. **Julie Louise Munk Andersen:** writing – review and editing. **Agnetha Linn Rostgaard‐Hansen:** writing – review and editing. **Léa Bouteille:** writing – review and editing. **Vinciane Rebours:** writing – review and editing. **Thérèse Truong:** writing – review and editing. **Matthias B. Schulze:** writing – review and editing. **Benedetta Bendinelli:** writing – review and editing. **Valeria Pala:** writing – review and editing. **Vittorio Simeon:** writing – review and editing. **Rosario Tumino:** writing – review and editing. **Carlotta Sacerdote:** writing – review and editing. **Roel Vermeulen:** writing – review and editing. **P. Martijn Kolijn:** writing – review and editing. **Sjoerd G. Elias:** writing – review and editing. **Marta Crous‐Bou:** writing – review and editing. **Maria‐José Sánchez:** writing – review and editing. **Ana Jimenez‐Zabala:** writing – review and editing. **José María Huerta:** writing – review and editing. **Marcela Guevara:** writing – review and editing. **Nick Wareham:** writing – review and editing. **Marie Breeur:** writing – review and editing. **Mattias Johansson:** writing – review and editing. **James Yarmolinsky:** writing – review and editing. **Daniele Campa:** writing – review and editing. **Rudolf Kaaks:** conceptualization, methodology, data curation, investigation, validation, supervision, visualization, project administration, resources, writing – original draft, writing – review and editing.

## Funding

The authors received no specific funding for this work. The coordination of EPIC‐Europe is financially supported by the International Agency for Research on Cancer (IARC) and also by the Department of Epidemiology and Biostatistics, School of Public Health, Imperial College London, which has additional infrastructure support provided by the NIHR Imperial Biomedical Research Centre (BRC). The national cohorts are supported by: Danish Cancer Society (Denmark); Ligue Nationale Contre le Cancer, Institut Gustave Roussy, Mutuelle Générale de l'Education Nationale (MGEN), Institut National de la Santé et de la Recherche Médicale (INSERM), French National Research Agency (ANR, reference ANR‐10‐COHO‐0006), French Ministry for Higher Education (subsidy 2102918823, 2103236497, and 2103586016) (France); German Cancer Aid, German Cancer Research Center (DKFZ), German Institute of Human Nutrition Potsdam‐Rehbruecke (DIfE), Federal Ministry of Research, Technology and Space (BMFTR) (Germany); Associazione Italiana per la Ricerca sul Cancro‐AIRC‐Italy, Italian Ministry of Health, Italian Ministry of University and Research (MUR), Compagnia di San Paolo (Italy); Dutch Ministry of Public Health, Welfare and Sports (VWS), the Netherlands Organisation for Health Research and Development (ZonMW), World Cancer Research Fund (WCRF), (The Netherlands); UiT The Arctic University of Norway; Health Research Fund (FIS)—Instituto de Salud Carlos III (ISCIII), Regional Governments of Andalucía, Asturias, Basque Country, Murcia and Navarra, and the Catalan Institute of Oncology—ICO (Spain); Swedish Cancer Society, Swedish Research Council of Region Skåne and Region Västerbotten (Sweden); Cancer Research UK (C864/A14136 to EPIC‐Norfolk; C8221/A29017 to EPIC‐Oxford), Medical Research Council (MR/N003284/1, MC‐UU_12015/1 and MC_UU_00006/1 to EPIC‐Norfolk (DOI 10.22025/2019.10.105.00004); MR/Y013662/1 to EPIC‐Oxford) (United Kingdom). Previous support has come from the “Europe against Cancer” Program of the European Commission (DG SANCO).

## Disclosure

Where authors are identified as personnel of the International Agency for Research on Cancer/World Health Organization, the authors alone are responsible for the views expressed in this article and they do not necessarily represent the decisions, policy or views of the International Agency for Research on Cancer/World Health Organization.

## Ethics Statement

All EPIC study participants have given written consent for future analyses of their data and blood samples for research purposes. The EPIC study was approved by the International Agency for Research on Cancer (IARC, Lyon, France) Ethics Committee and by the local study center ethical committees.

## Conflicts of Interest

The authors declare no conflicts of interest.

## Supporting information


**Table S1:** Proteins in the Olink immune‐oncology panel.
**Figure S1:** Flow diagram of participants in the EPIC pancreatic cancer nested case–control study.
**Figure S2:** Distribution of missing values and LOD values in the original EPIC nested case–control dataset.
**Figure S3:** Histogram and density of biomarkers after imputation and replacement of LOD values, among controls.
**Figure S4:** Spearman partial correlation coefficients between proteins with hierarchical clustering, among controls, basic adjusted.
**Figure S5:** Forest plot of Spearman partial correlation coefficients of proteins with age at recruitment, among controls.
**Figure S6:** Forest plot of Spearman partial correlation coefficients of proteins with BMI, among controls.
**Figure S7:** Mean and 95% CI of biomarkers by sex, among controls.
**Figure S8:** Mean and 95% CI of Biomarkers by Smoking Status, among controls.
**Figure S9:** Mean and 95% CI of Biomarkers by Alcohol Consumption, among controls.
**Figure S10:** Mean and 95% CI of Biomarkers by Self‐reported Diabetes Status, among controls.
**Figure S11:** Spearman partial correlation coefficients between the 8 significant biomarkers, among controls.

## Data Availability

The data that support the findings of this study are available from the corresponding author upon reasonable request.
