# Supplementary material for: Immune Markers and Risk of Pancreatic Cancer in the European EPIC Cohort
Source: Int J Cancer. 2026 Jun 23;159(9):2146–57. doi: 10.1002/ijc.70581 (PMC13547983; doi:10.1002/ijc.70581)
Supplement: Supplementary file 1 [file IJC-159-2146-s001.pdf]

SUPPLEMENTARY MATERIAL to

Title:

Immune markers and risk of pancreatic cancer in the European EPIC cohort

Authors:

Verena A Katzke, Yue Chen, Srimanti Dutta, Federico Canzian, Julie Louise Munk Andersen, Agnetha Linn Rostgaard-Hansen, Agnetha Linn Rostgaard-Hansen, Léa Bouteille, Vinciane Rebours, Thérèse Truong, Matthias B. Schulze, Benedetta Bendinelli, Valeria Pala, Vittorio Simeon, Rosario Tumino, Carlotta Sacerdote, Roel Vermeulen, P Martijn Kolijn, Sjoerd G Elias, Marta Crous-Bou, Maria-José Sánchez, Ana Jimenez-Zabala, José María Huerta, Marcela Guevara, Nick Wareham, Marie Breeur, Mattias Johansson, James Yarmolinsky, Daniele Campa, Rudolf Kaaks

Table of contents:

Table S1: Proteins in the Olink® immune-oncology panel

Figure S1: Flow diagram of participants in the EPIC pancreatic cancer nested case-control study

Figure S2: Distribution of missing values and LOD values in the original EPIC nested case-control dataset

Figure S3: Histogram and density of biomarkers after imputation and replacement of LOD values, among controls

Figure S4: Spearman partial correlation coefficients between proteins with hierarchical clustering, among controls, basic adjusted

Figure S5: Forest plot of Spearman partial correlation coefficients of proteins with age at recruitment, among controls

Figure S6: Forest plot of Spearman partial correlation coefficients of proteins with BMI, among controls

Figure S7: Mean and 95% CI of biomarkers by sex, among controls

Figure S8: Mean and 95% CI of Biomarkers by Smoking Status, among controls

Figure S9: Mean and 95% CI of Biomarkers by Alcohol Consumption, among controls

Figure S10: Mean and 95% CI of Biomarkers by Self-reported Diabetes Status, among controls

Figure S11: Spearman partial correlation coefficients between the 8 significant biomarkers, among controls

Table S1: Proteins in the Olink® immune-oncology panel

UniProt ID	Gene	Protein name
P00813	ADA	Adenosine deaminase
Q9Y653	ADGRG1	Adhesion G-protein coupled receptor G1
Q15389	ANGPT1	Angiopoietin-1
O15123	ANGPT2	Angiopoietin-2
P05089	ARG1	Arginase-1
Q16790	CA9	Carbonic anhydrase 9 / CAIX
Q14790	CASP8	Caspase-8
Q92583	CCL17	C-C motif chemokine 17
Q99731	CCL19	C-C motif chemokine 19
P78556	CCL20	C-C motif chemokine 20
P55773	CCL23	C-C motif chemokine 23
P10147	CCL3	C-C motif chemokine 3
P13236	CCL4	C-C motif chemokine 4
Q99616	CCL13	C-C motif chemokine 13 / MCP4
P13500	CCL2	C-C motif chemokine 2 / MCP1
P80098	CCL7	C-C motif chemokine 7 / MCP3
P80075	CCL8	C-C motif chemokine 8 / MCP2
Q9BZW8	CD244	Natural killer cell receptor 2B4
P26842	CD27	CD27 antigen
P10747	CD28	T-cell-specific surface glycoprotein CD28
P01730	CD4	T-cell surface glycoprotein CD4
P25942	CD40	Tumor necrosis factor receptor superfamily member 5
P29965	CD40LG	CD40 ligand / CD40.L
P06127	CD5	T-cell surface glycoprotein CD5
P32970	CD70	CD70 antigen
Q01151	CD83	CD83 antigen
P01732	CD8A	T-cell surface glycoprotein CD8 alpha chain
O95727	CRTAM	Cytotoxic and regulatory T-cell molecule
P09603	CSF1	Macrophage colony-stimulating factor 1
P78423	CX3CL1	Fractalkine
P09341	CXCL1	Growth-regulated alpha protein
P02778	CXCL10	C-X-C motif chemokine 10
O14625	CXCL11	C-X-C motif chemokine 11
P48061	CXCL12	Stromal cell-derived factor 1
O43927	CXCL13	C-X-C motif chemokine 13
P42830	CXCL5	C-X-C motif chemokine 5
P10145	CXCL8	Interleukin-8 / IL8
Q07325	CXCL9	C-X-C motif chemokine 9
P07585	DCN	Decorin
P01133	EGF	Pro-epidermal growth factor
P48023	FASLG	Tumor necrosis factor ligand superfamily member 6
P09038	FGF2	Fibroblast growth factor 2
P12544	GZMA	Granzyme A
P10144	GZMB	Granzyme B
P20718	GZMH	Granzyme H
P14210	HGF	Hepatocyte growth factor
P09601	HMOX1	Heme oxygenase 1 / HO.1
O75144	ICOSLG	ICOS ligand

P01579	IFNG	Interferon gamma / IFN.gamma
P01583	IL1A	Interleukin-1 alpha / IL.1.alpha
P22301	IL10	Interleukin-10
P29459_P29460	IL12A_IL12B	Interleukin-12 / IL12
P42701	IL12RB1	Interleukin-12 receptor subunit beta-1
P35225	IL13	Interleukin-13
P40933	IL15	Interleukin-15
Q14116	IL18	Interleukin-18
P60568	IL2	Interleukin-2
O95760	IL33	Interleukin-33
P05112	IL4	Interleukin-4
P05113	IL5	Interleukin-5
P05231	IL6	Interleukin-6
P13232	IL7	Interleukin-7
P43629	KIR3DL1	Killer cell immunoglobulin-like receptor 3DL1
Q13241	KLRD1	Natural killer cells antigen CD94
P18627	LAG3	Lymphocyte activation gene 3 protein
Q9UQV4	LAMP3	Lysosome-associated membrane glycoprotein 3
P09382	LGALS1	Galectin-1 / Gal.1
O00182	LGALS9	Galectin-9 / Gal.9
P01137	TGFB1	Transforming growth factor beta-1 proprotein / LAP.TGF.beta.1
Q29983_Q29980	MICA_MICB	MHC class I polypeptide-related sequence A and sequence B / / MIC.A.B
P39900	MMP12	Macrophage metalloelastase
P09237	MMP7	Matrilysin
Q8WXI7	MUC16	Mucin-16
O76036	NCR1	Natural cytotoxicity triggering receptor 1
P29474	NOS3	Nitric oxide synthase, endothelial
Q9NZQ7	CD274	Programmed cell death 1 ligand 1 / PD.L1
Q9BQ51	PDCD1LG2	Programmed cell death 1 ligand 2 / PD.L2
Q15116	PDCD1	Programmed cell death protein 1
P01127	PDGFB	Platelet-derived growth factor subunit B / PDGF.subunit.B
P49763	PGF	Placenta growth factor
P21246	PTN	Pleiotrophin
Q02763	TEK	Angiopoietin-1 receptor / TIE2
P01375	TNF	Tumor necrosis factor
Q9NP84	TNFRSF12A	Tumor necrosis factor receptor superfamily member 12A
O75509	TNFRSF21	Tumor necrosis factor receptor superfamily member 21
P43489	TNFRSF4	Tumor necrosis factor receptor superfamily member 4
Q07011	TNFRSF9	Tumor necrosis factor receptor superfamily member 9
P50591	TNFSF10	Tumor necrosis factor ligand superfamily member 10 / TRAIL
O43508	TNFSF12	Tumor necrosis factor ligand superfamily member 12 / TWEAK
O43557	TNFSF14	Tumor necrosis factor ligand superfamily member 14
P15692	VEGFA	Vascular endothelial growth factor A
P35968	KDR	Vascular endothelial growth factor receptor 2 / VEGFR.2

Figure S1: Flow diagram of participants in the EPIC pancreatic cancer nested case-control study

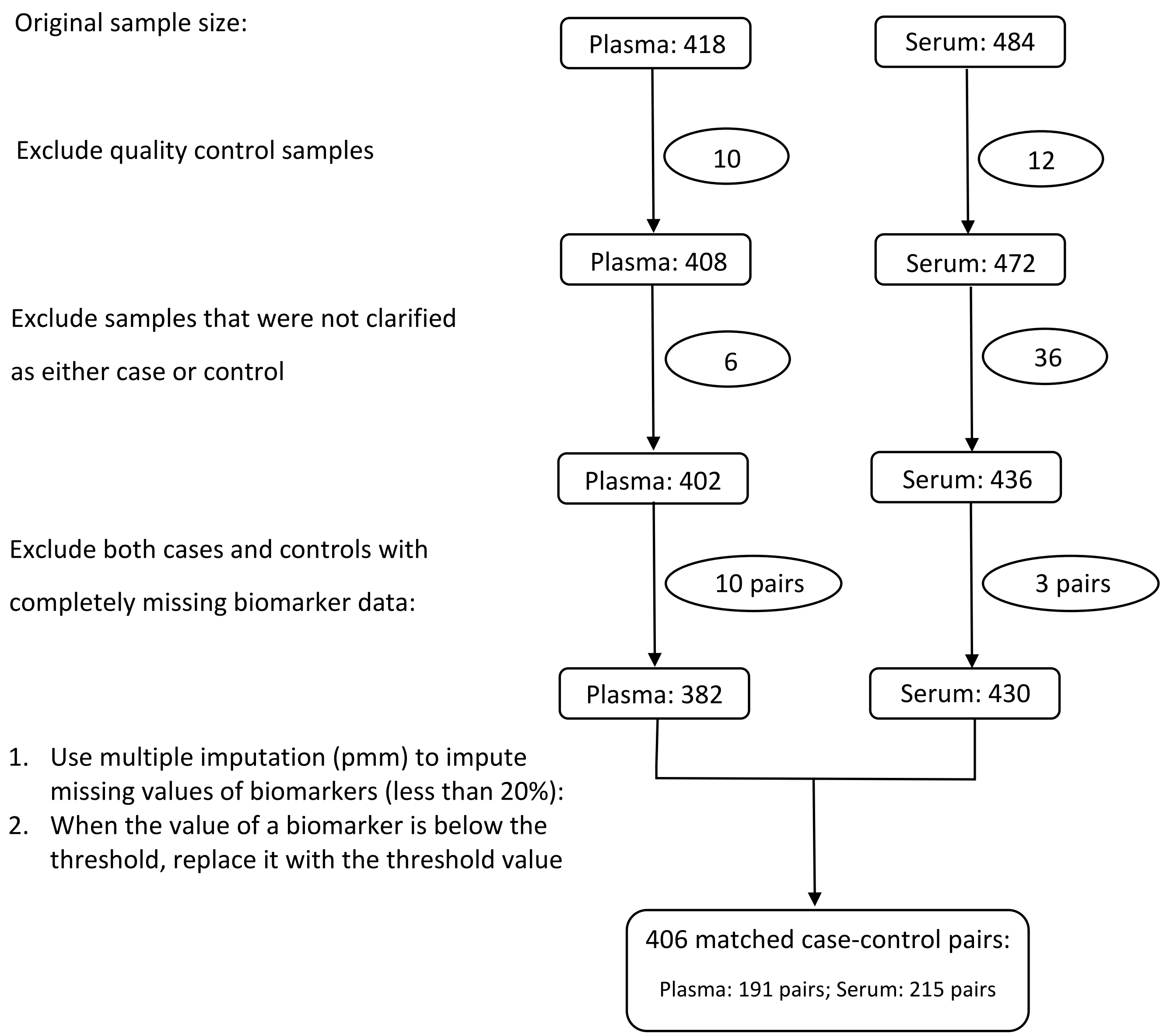

Figure S2: Distribution of missing values and LOD values in the original EPIC nested case-control dataset

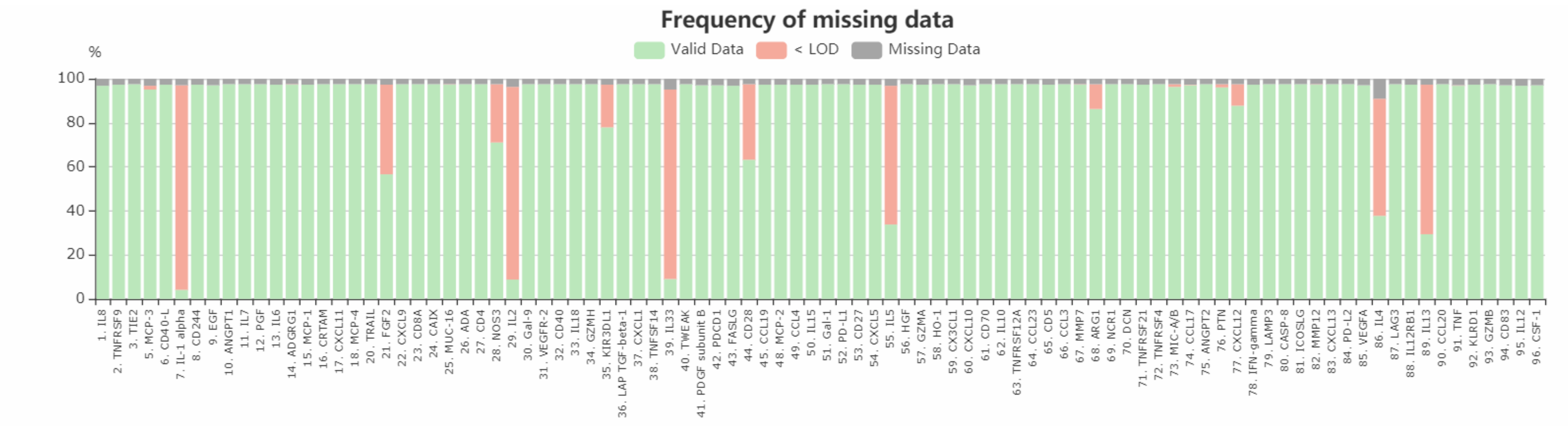

Figure S3: Histogram and density of biomarkers after imputation and replacement of LOD values, among controls

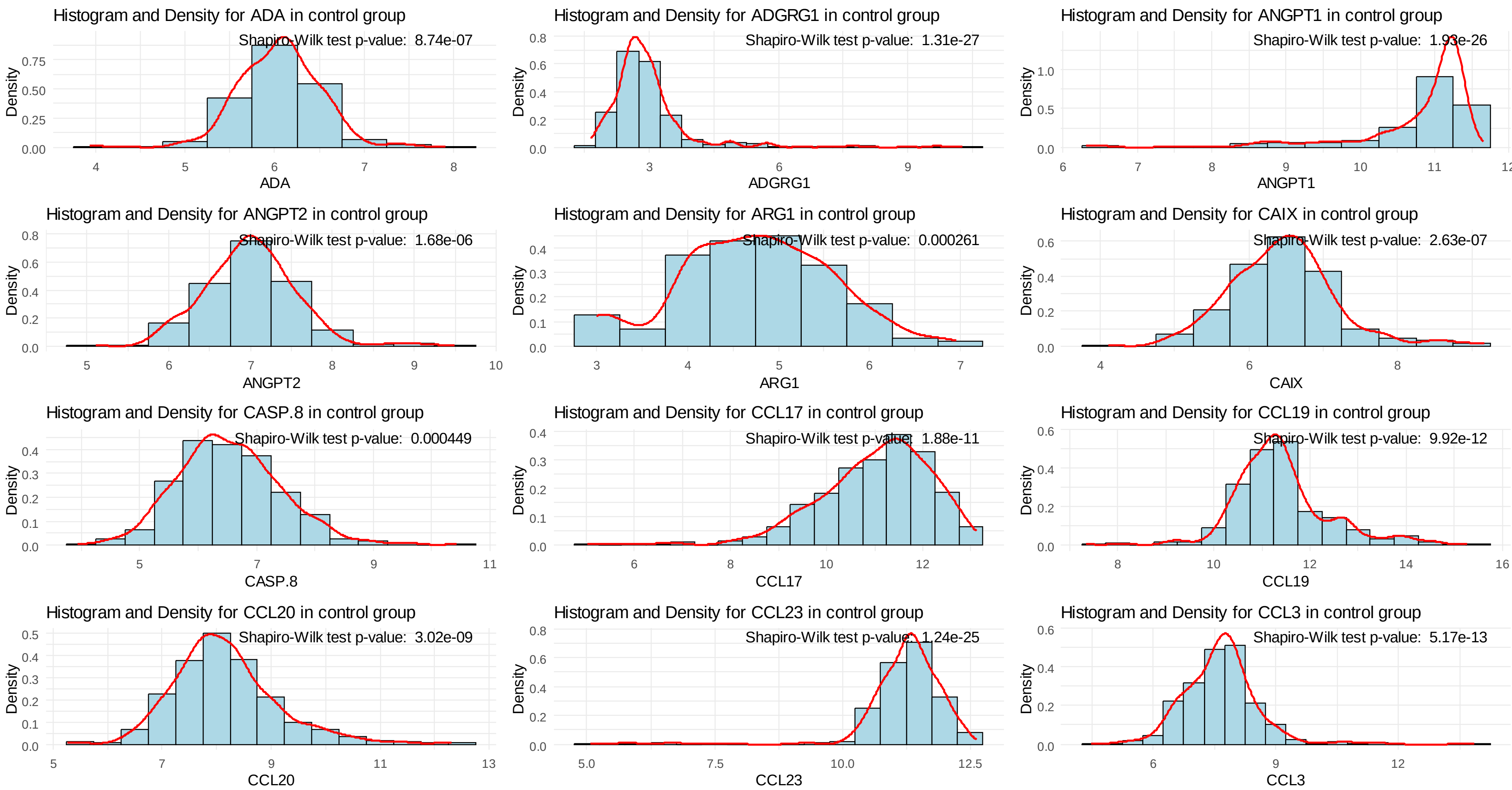

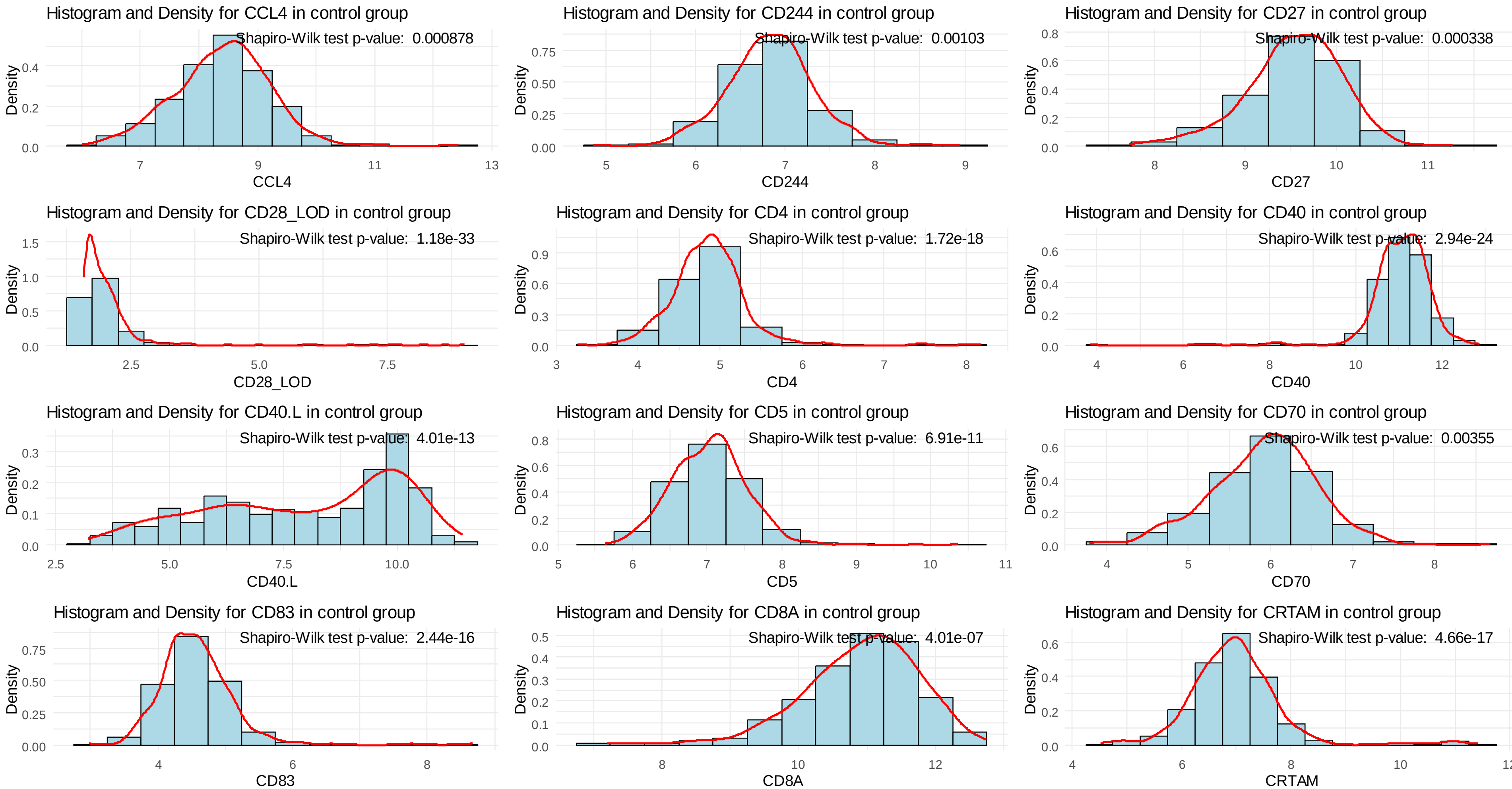
Histogram and Density for CCL4 in control group
Shapiro-Wilk test p-value: 0.000878
Density
CCL4
Histogram and Density for CD244 in control group
Shapiro-Wilk test p-value: 0.00103
Density
CD244
Histogram and Density for CD27 in control group
Shapiro-Wilk test p-value: 0.000338
Density
CD27
Histogram and Density for CD28_LOD in control group
Shapiro-Wilk test p-value: 1.18e-33
Density
CD28_LOD
Histogram and Density for CD4 in control group
Shapiro-Wilk test p-value: 1.72e-18
Density
CD4
Histogram and Density for CD40 in control group
Shapiro-Wilk test p-value: 2.94e-24
Density
CD40
Histogram and Density for CD40.L in control group
Shapiro-Wilk test p-value: 4.01e-13
Density
CD40.L
Histogram and Density for CD5 in control group
Shapiro-Wilk test p-value: 6.91e-11
Density
CD5
Histogram and Density for CD70 in control group
Shapiro-Wilk test p-value: 0.00355
Density
CD70
Histogram and Density for CD83 in control group
Shapiro-Wilk test p-value: 2.44e-16
Density
CD83
Histogram and Density for CD8A in control group
Shapiro-Wilk test p-value: 4.01e-07
Density
CD8A
Histogram and Density for CRTAM in control group
Shapiro-Wilk test p-value: 4.66e-17
Density
CRTAM

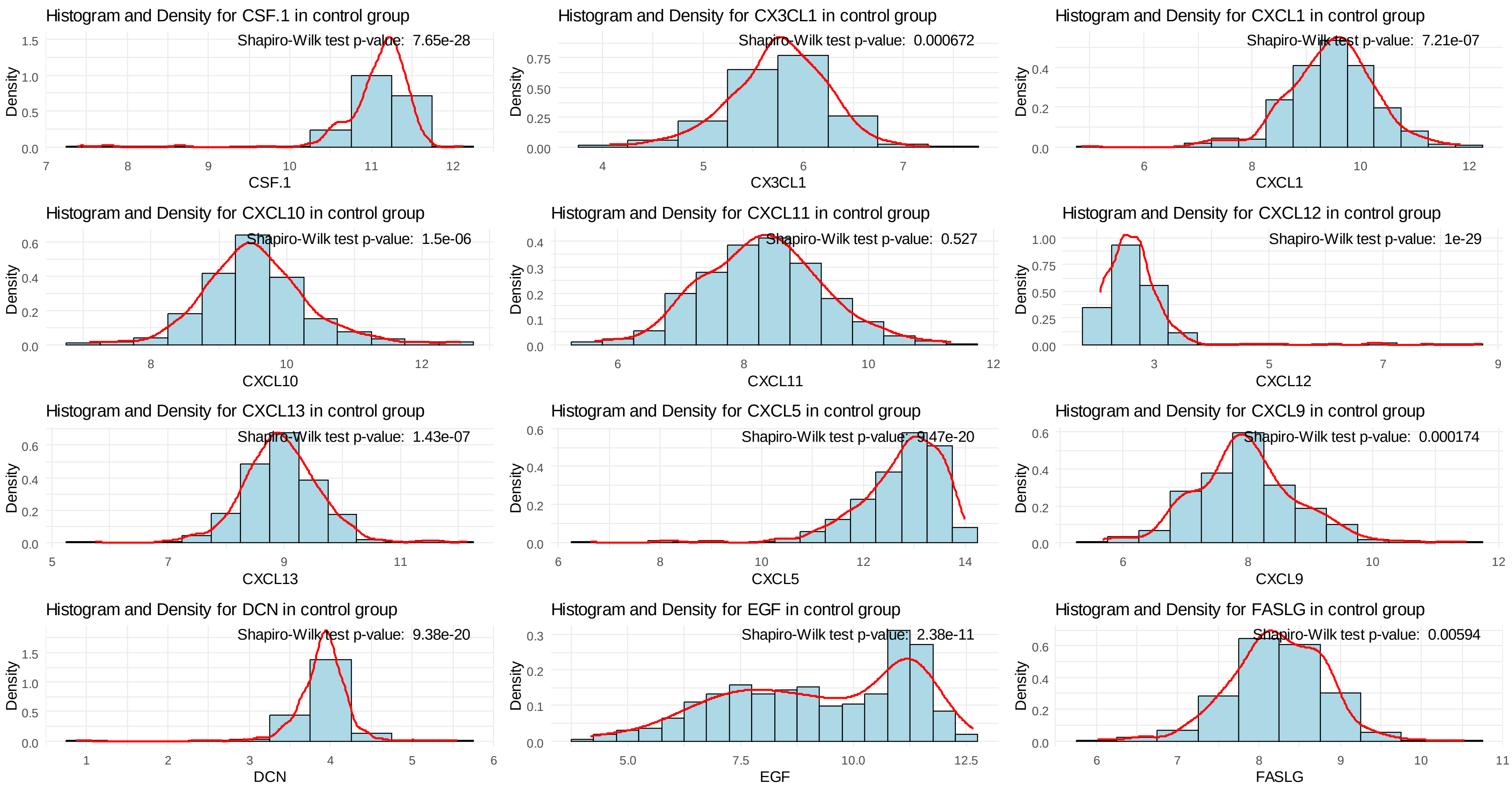
Histogram and Density for CSF.1 in control group
Shapiro-Wilk test p-value: 7.65e-28
Density
CSF.1
Histogram and Density for CX3CL1 in control group
Shapiro-Wilk test p-value: 0.000672
Density
CX3CL1
Histogram and Density for CXCL1 in control group
Shapiro-Wilk test p-value: 7.21e-07
Density
CXCL1
Histogram and Density for CXCL10 in control group
Shapiro-Wilk test p-value: 1.5e-06
Density
CXCL10
Histogram and Density for CXCL11 in control group
Shapiro-Wilk test p-value: 0.527
Density
CXCL11
Histogram and Density for CXCL12 in control group
Shapiro-Wilk test p-value: 1e-29
Density
CXCL12
Histogram and Density for CXCL13 in control group
Shapiro-Wilk test p-value: 1.43e-07
Density
CXCL13
Histogram and Density for CXCL5 in control group
Shapiro-Wilk test p-value: 9.47e-20
Density
CXCL5
Histogram and Density for CXCL9 in control group
Shapiro-Wilk test p-value: 0.000174
Density
CXCL9
Histogram and Density for DCN in control group
Shapiro-Wilk test p-value: 9.38e-20
Density
DCN
Histogram and Density for EGF in control group
Shapiro-Wilk test p-value: 2.38e-11
Density
EGF
Histogram and Density for FASLG in control group
Shapiro-Wilk test p-value: 0.00594
Density
FASLG

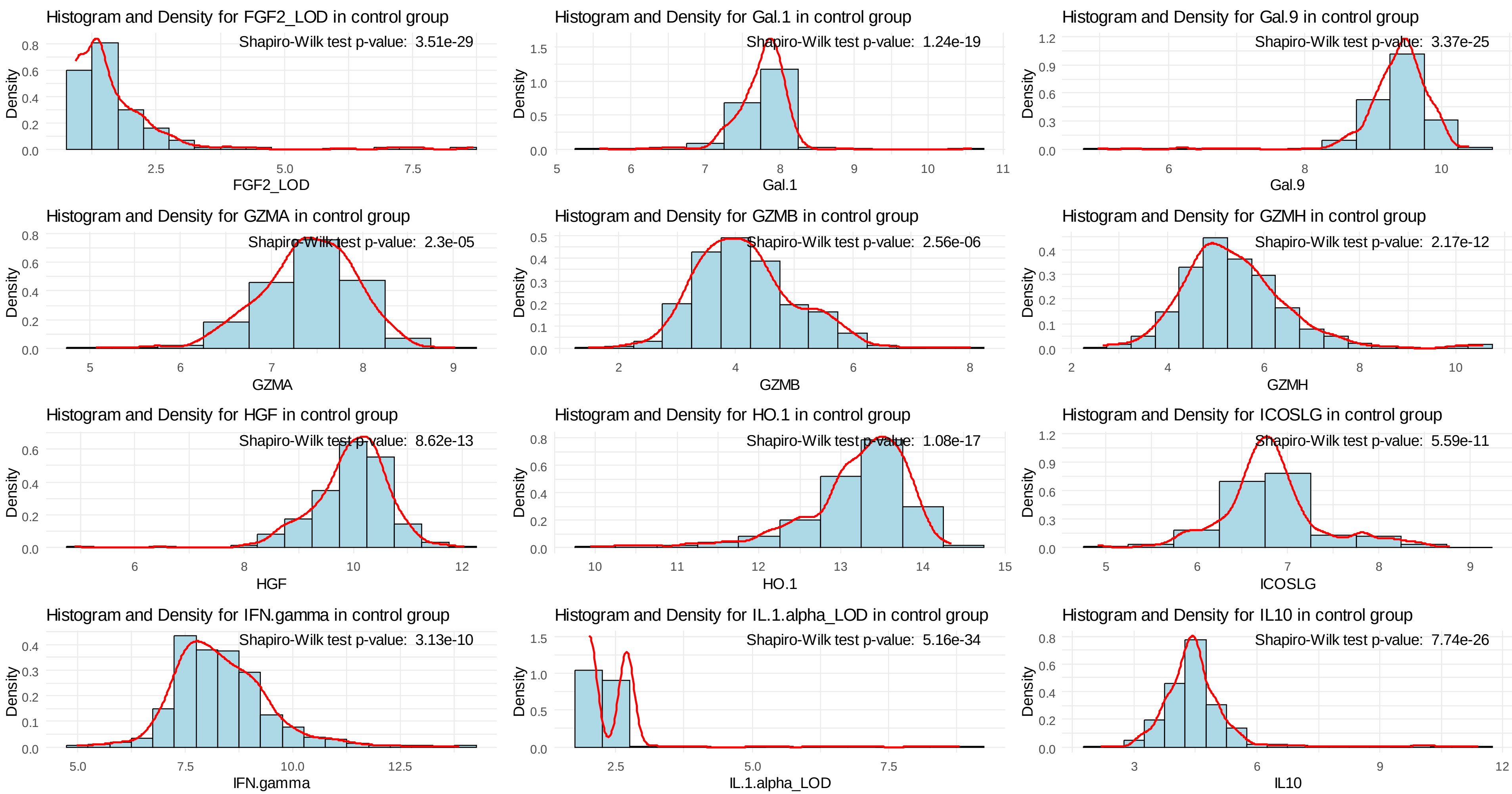

Histogram and Density for FGF2_LOD in control group
Shapiro-Wilk test p-value: 3.51e-29
Density
FGF2_LOD
Histogram and Density for Gal.1 in control group
Shapiro-Wilk test p-value: 1.24e-19
Density
Gal.1
Histogram and Density for Gal.9 in control group
Shapiro-Wilk test p-value: 3.37e-25
Density
Gal.9
Histogram and Density for GZMA in control group
Shapiro-Wilk test p-value: 2.3e-05
Density
GZMA
Histogram and Density for GZMB in control group
Shapiro-Wilk test p-value: 2.56e-06
Density
GZMB
Histogram and Density for GZMH in control group
Shapiro-Wilk test p-value: 2.17e-12
Density
GZMH
Histogram and Density for HGF in control group
Shapiro-Wilk test p-value: 8.62e-13
Density
HGF
Histogram and Density for HO.1 in control group
Shapiro-Wilk test p-value: 1.08e-17
Density
HO.1
Histogram and Density for ICOSLG in control group
Shapiro-Wilk test p-value: 5.59e-11
Density
ICOSLG
Histogram and Density for IFN.gamma in control group
Shapiro-Wilk test p-value: 3.13e-10
Density
IFN.gamma
Histogram and Density for IL.1.alpha_LOD in control group
Shapiro-Wilk test p-value: 5.16e-34
Density
IL.1.alpha_LOD
Histogram and Density for IL10 in control group
Shapiro-Wilk test p-value: 7.74e-26
Density
IL10

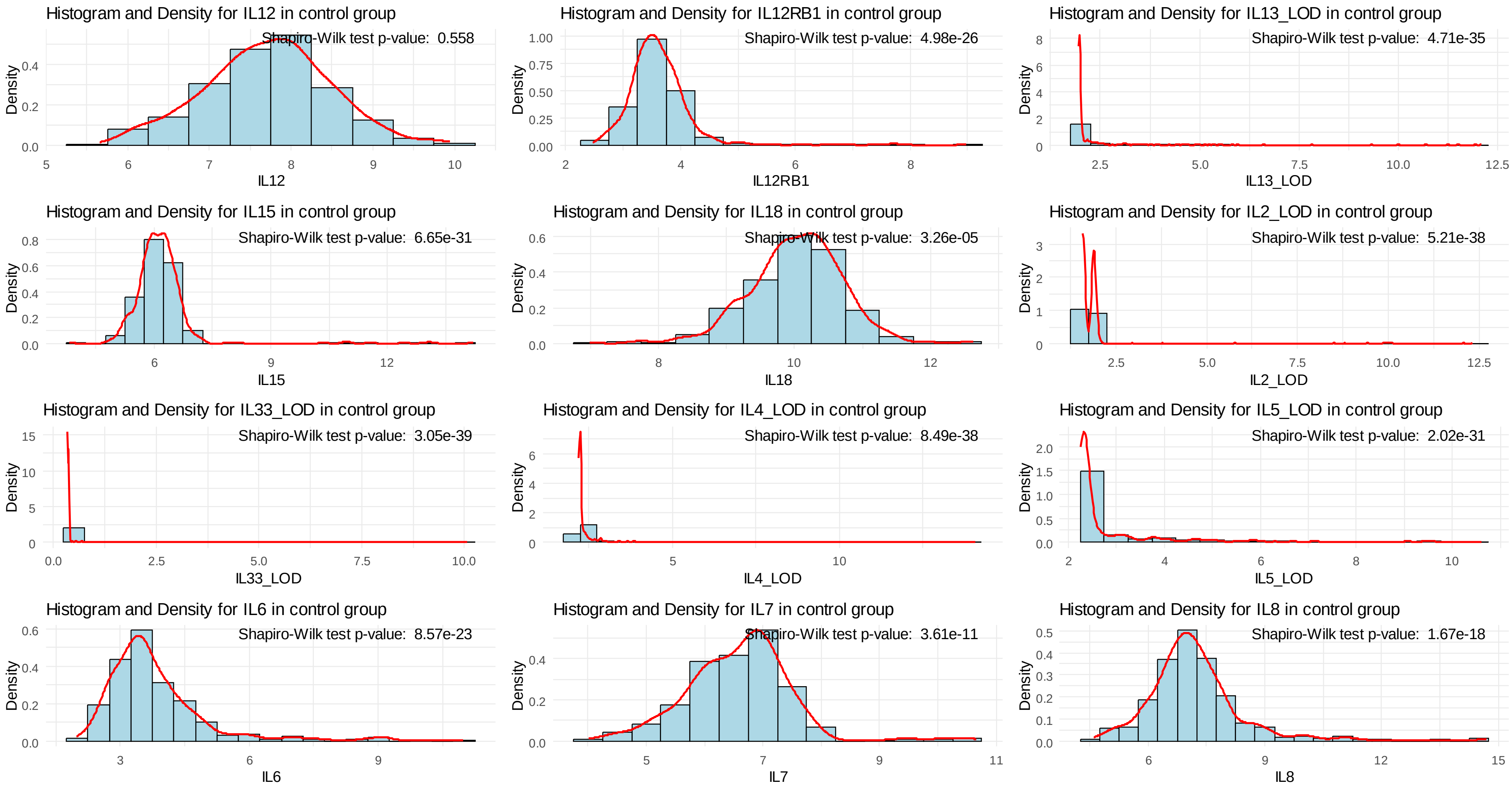
Histogram and Density for IL12 in control group
Shapiro-Wilk test p-value: 0.558
Density
IL12
Histogram and Density for IL12RB1 in control group
Shapiro-Wilk test p-value: 4.98e-26
Density
IL12RB1
Histogram and Density for IL13_LOD in control group
Shapiro-Wilk test p-value: 4.71e-35
Density
IL13_LOD
Histogram and Density for IL15 in control group
Shapiro-Wilk test p-value: 6.65e-31
Density
IL15
Histogram and Density for IL18 in control group
Shapiro-Wilk test p-value: 3.26e-05
Density
IL18
Histogram and Density for IL2_LOD in control group
Shapiro-Wilk test p-value: 5.21e-38
Density
IL2_LOD
Histogram and Density for IL33_LOD in control group
Shapiro-Wilk test p-value: 3.05e-39
Density
IL33_LOD
Histogram and Density for IL4_LOD in control group
Shapiro-Wilk test p-value: 8.49e-38
Density
IL4_LOD
Histogram and Density for IL5_LOD in control group
Shapiro-Wilk test p-value: 2.02e-31
Density
IL5_LOD
Histogram and Density for IL6 in control group
Shapiro-Wilk test p-value: 8.57e-23
Density
IL6
Histogram and Density for IL7 in control group
Shapiro-Wilk test p-value: 3.61e-11
Density
IL7
Histogram and Density for IL8 in control group
Shapiro-Wilk test p-value: 1.67e-18
Density
IL8

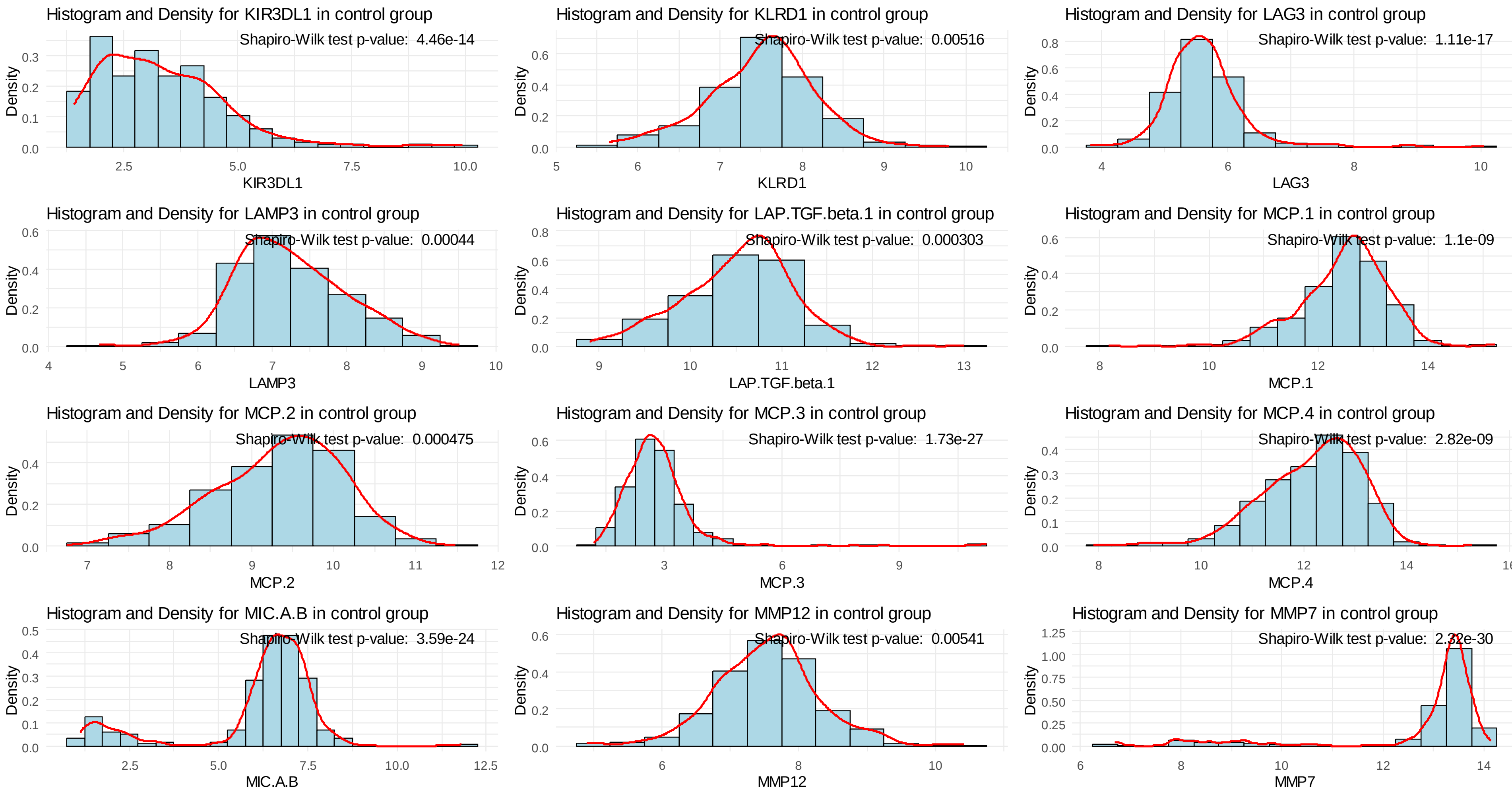
Histogram and Density for KIR3DL1 in control group
Shapiro-Wilk test p-value: 4.46e-14
Density
KIR3DL1
Histogram and Density for KLRD1 in control group
Shapiro-Wilk test p-value: 0.00516
Density
KLRD1
Histogram and Density for LAG3 in control group
Shapiro-Wilk test p-value: 1.11e-17
Density
LAG3
Histogram and Density for LAMP3 in control group
Shapiro-Wilk test p-value: 0.00044
Density
LAMP3
Histogram and Density for LAP.TGF.beta.1 in control group
Shapiro-Wilk test p-value: 0.000303
Density
LAP.TGF.beta.1
Histogram and Density for MCP.1 in control group
Shapiro-Wilk test p-value: 1.1e-09
Density
MCP.1
Histogram and Density for MCP.2 in control group
Shapiro-Wilk test p-value: 0.000475
Density
MCP.2
Histogram and Density for MCP.3 in control group
Shapiro-Wilk test p-value: 1.73e-27
Density
MCP.3
Histogram and Density for MCP.4 in control group
Shapiro-Wilk test p-value: 2.82e-09
Density
MCP.4
Histogram and Density for MIC.A.B in control group
Shapiro-Wilk test p-value: 3.59e-24
Density
MIC.A.B
Histogram and Density for MMP12 in control group
Shapiro-Wilk test p-value: 0.00541
Density
MMP12
Histogram and Density for MMP7 in control group
Shapiro-Wilk test p-value: 2.32e-30
Density
MMP7

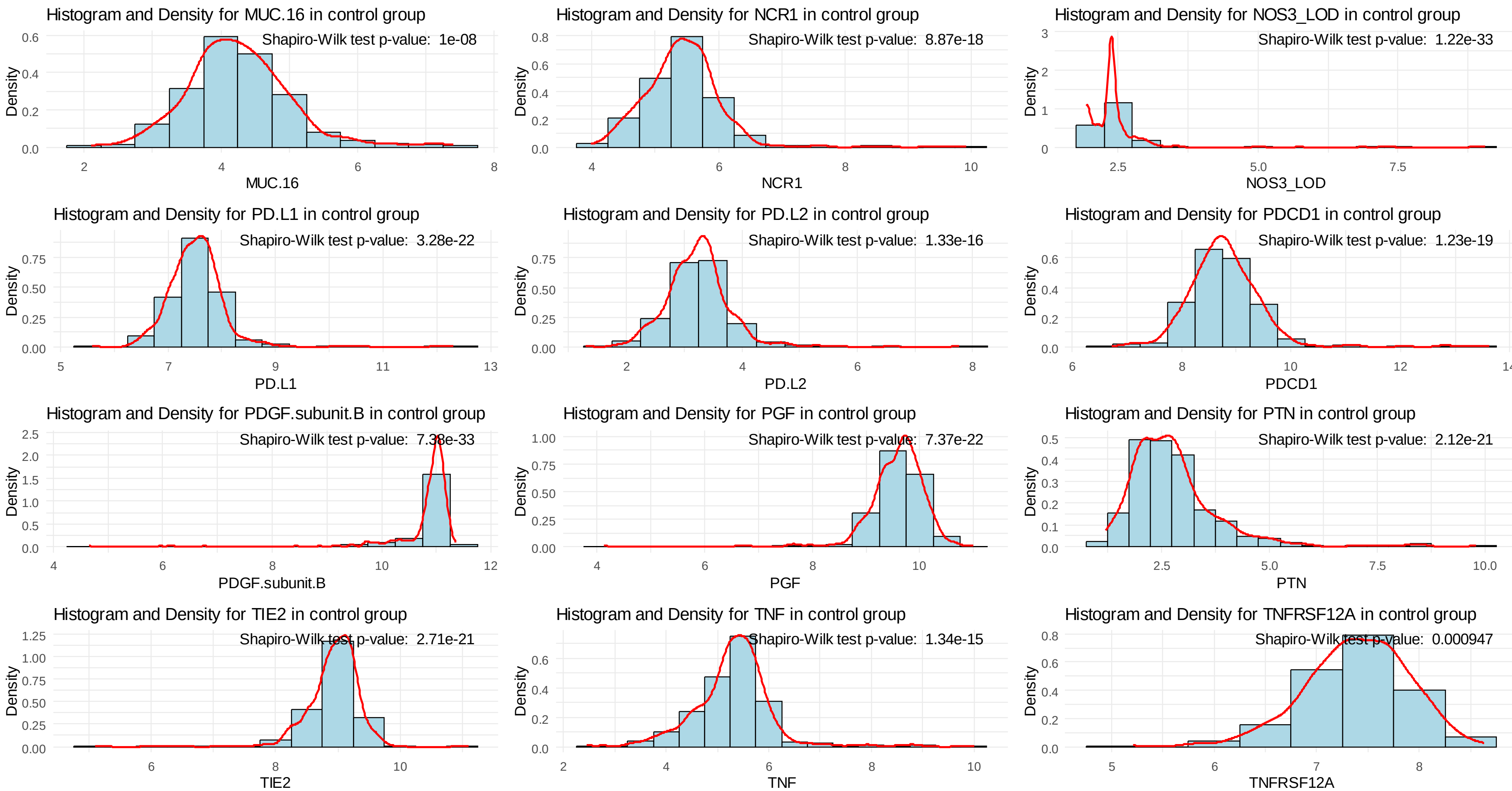
Histogram and Density for MUC.16 in control group
Shapiro-Wilk test p-value: 1e-08
Density
MUC.16
Histogram and Density for NCR1 in control group
Shapiro-Wilk test p-value: 8.87e-18
Density
NCR1
Histogram and Density for NOS3_LOD in control group
Shapiro-Wilk test p-value: 1.22e-33
Density
NOS3_LOD
Histogram and Density for PD.L1 in control group
Shapiro-Wilk test p-value: 3.28e-22
Density
PD.L1
Histogram and Density for PD.L2 in control group
Shapiro-Wilk test p-value: 1.33e-16
Density
PD.L2
Histogram and Density for PDCD1 in control group
Shapiro-Wilk test p-value: 1.23e-19
Density
PDCD1
Histogram and Density for PDGF.subunit.B in control group
Shapiro-Wilk test p-value: 7.38e-33
Density
PDGF.subunit.B
Histogram and Density for PGF in control group
Shapiro-Wilk test p-value: 7.37e-22
Density
PGF
Histogram and Density for PTN in control group
Shapiro-Wilk test p-value: 2.12e-21
Density
PTN
Histogram and Density for TIE2 in control group
Shapiro-Wilk test p-value: 2.71e-21
Density
TIE2
Histogram and Density for TNF in control group
Shapiro-Wilk test p-value: 1.34e-15
Density
TNF
Histogram and Density for TNFRSF12A in control group
Shapiro-Wilk test p-value: 0.000947
Density
TNFRSF12A

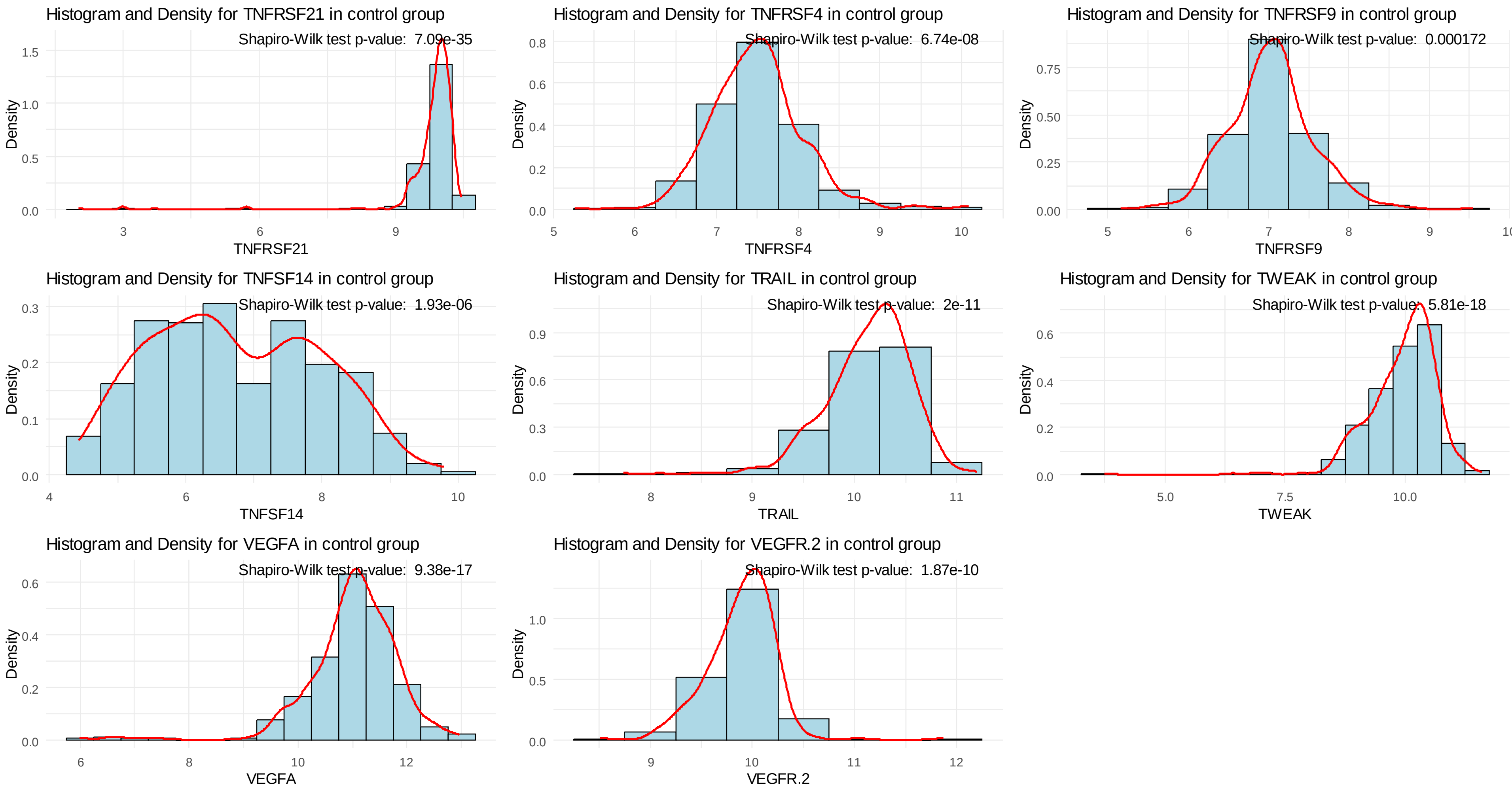

Histogram and Density for TNFRSF21 in control group
Shapiro-Wilk test p-value: 7.09e-35
Density
TNFRSF21
Histogram and Density for TNFRSF4 in control group
Shapiro-Wilk test p-value: 6.74e-08
Density
TNFRSF4
Histogram and Density for TNFRSF9 in control group
Shapiro-Wilk test p-value: 0.000172
Density
TNFRSF9
Histogram and Density for TNFSF14 in control group
Shapiro-Wilk test p-value: 1.93e-06
Density
TNFSF14
Histogram and Density for TRAIL in control group
Shapiro-Wilk test p-value: 2e-11
Density
TRAIL
Histogram and Density for TWEAK in control group
Shapiro-Wilk test p-value: 5.81e-18
Density
TWEAK
Histogram and Density for VEGFA in control group
Shapiro-Wilk test p-value: 9.38e-17
Density
VEGFA
Histogram and Density for VEGFR.2 in control group
Shapiro-Wilk test p-value: 1.87e-10
Density
VEGFR.2

Figure S4: Spearman partial correlation coefficients between proteins with hierarchical clustering, among controls, basic adjusted*

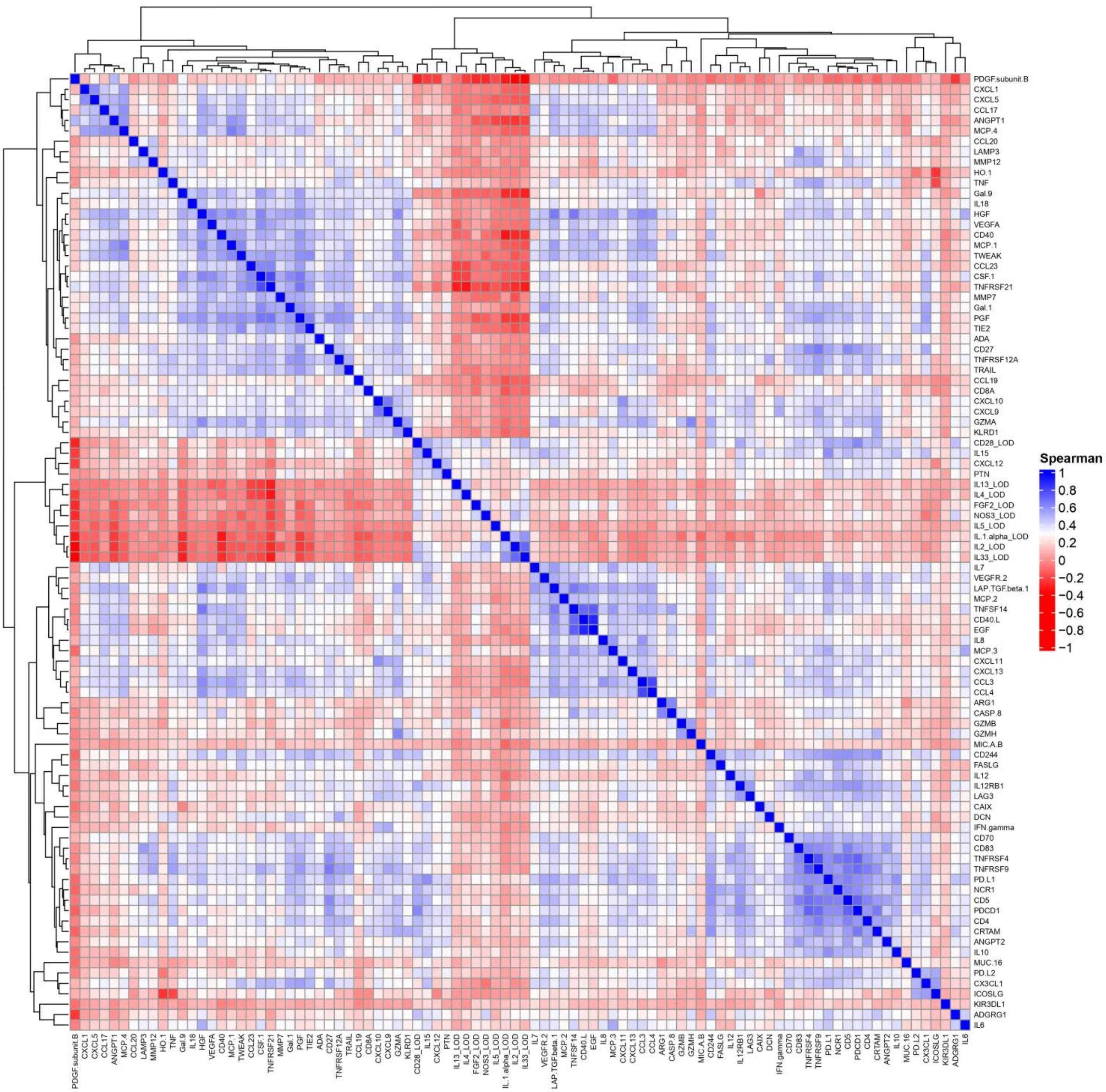

*Adjusted for batch, age at recruitment, sex and country

Figure S5: Forest plot of Spearman partial correlation coefficients of proteins with age at recruitment, among controls*

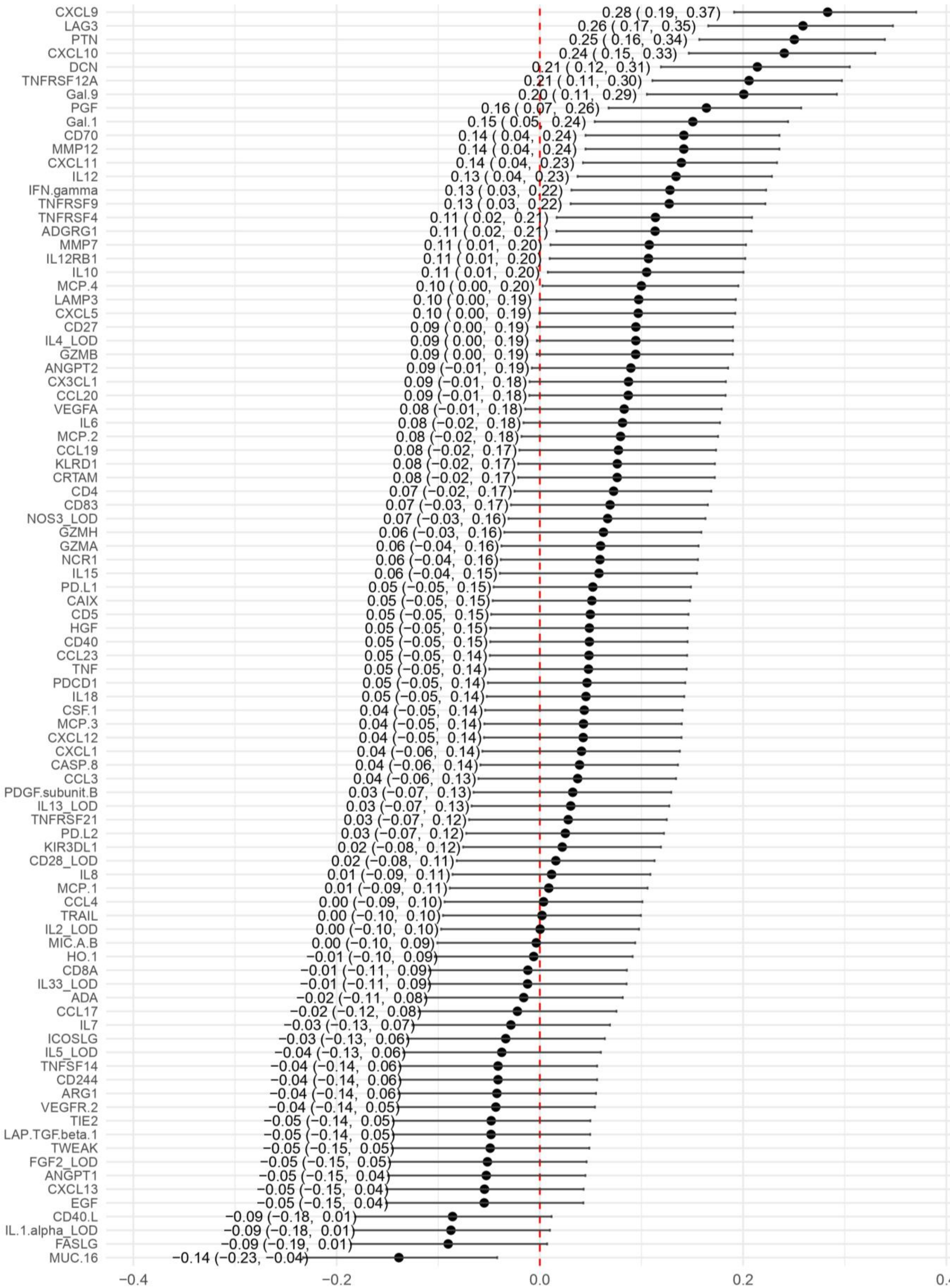

*Adjusted for country, batch and sex

Figure S6: Forest plot of Spearman partial correlation coefficients of proteins with BMI, among controls*

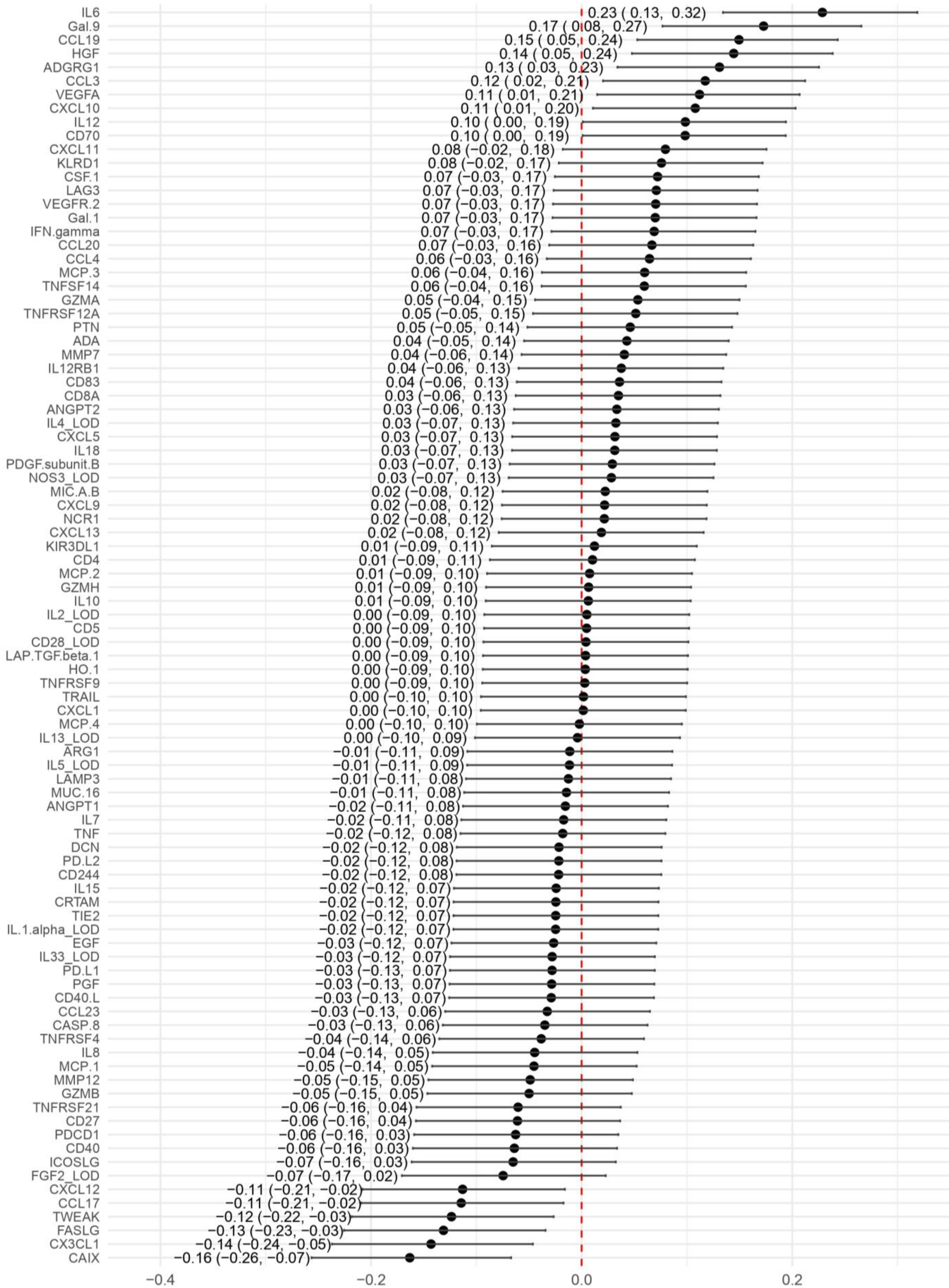

*Adjusted for batch, country, sex and age at recruitment

Figure S7: Mean and 95% CI of biomarkers by sex, among controls *

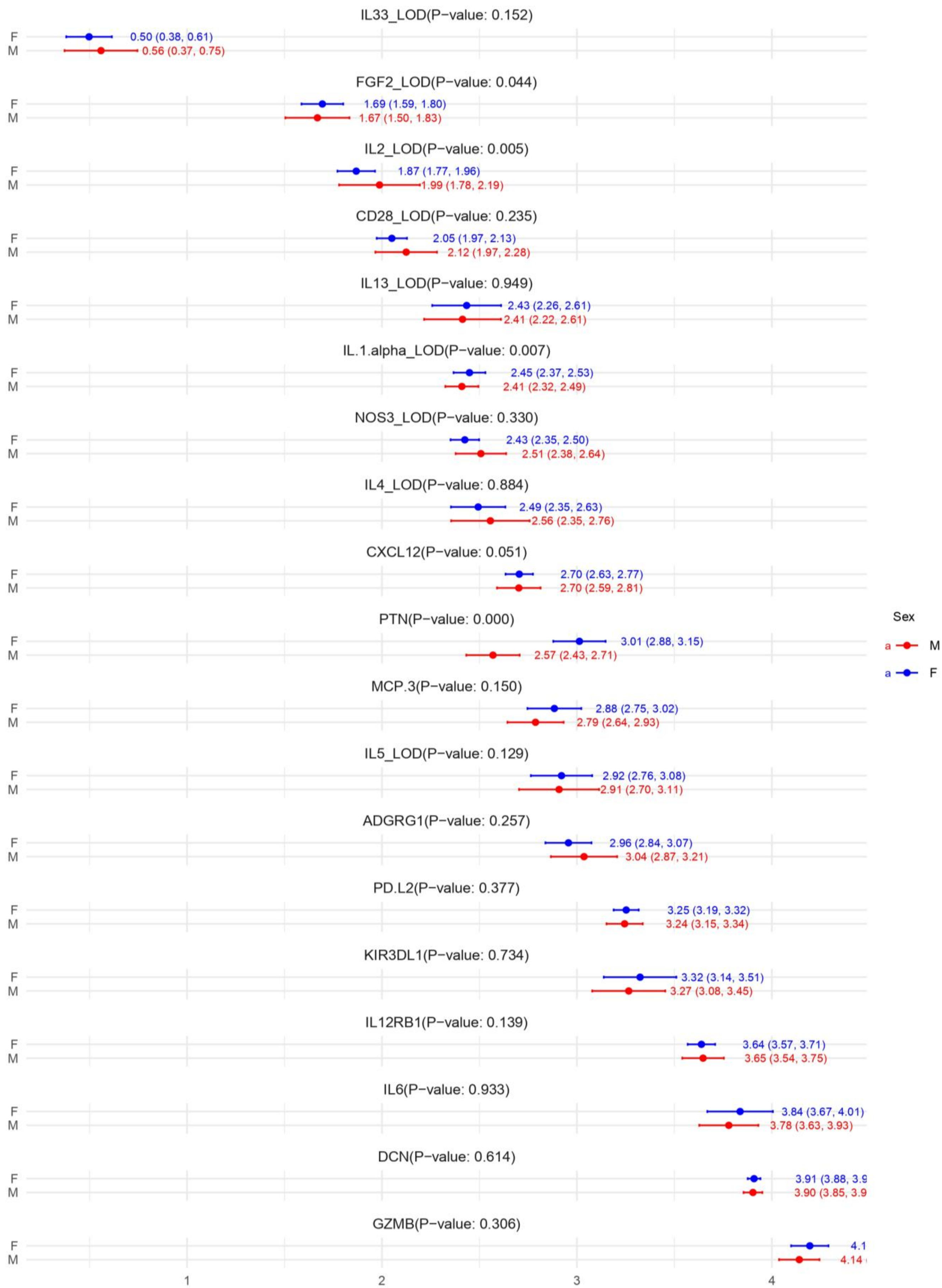

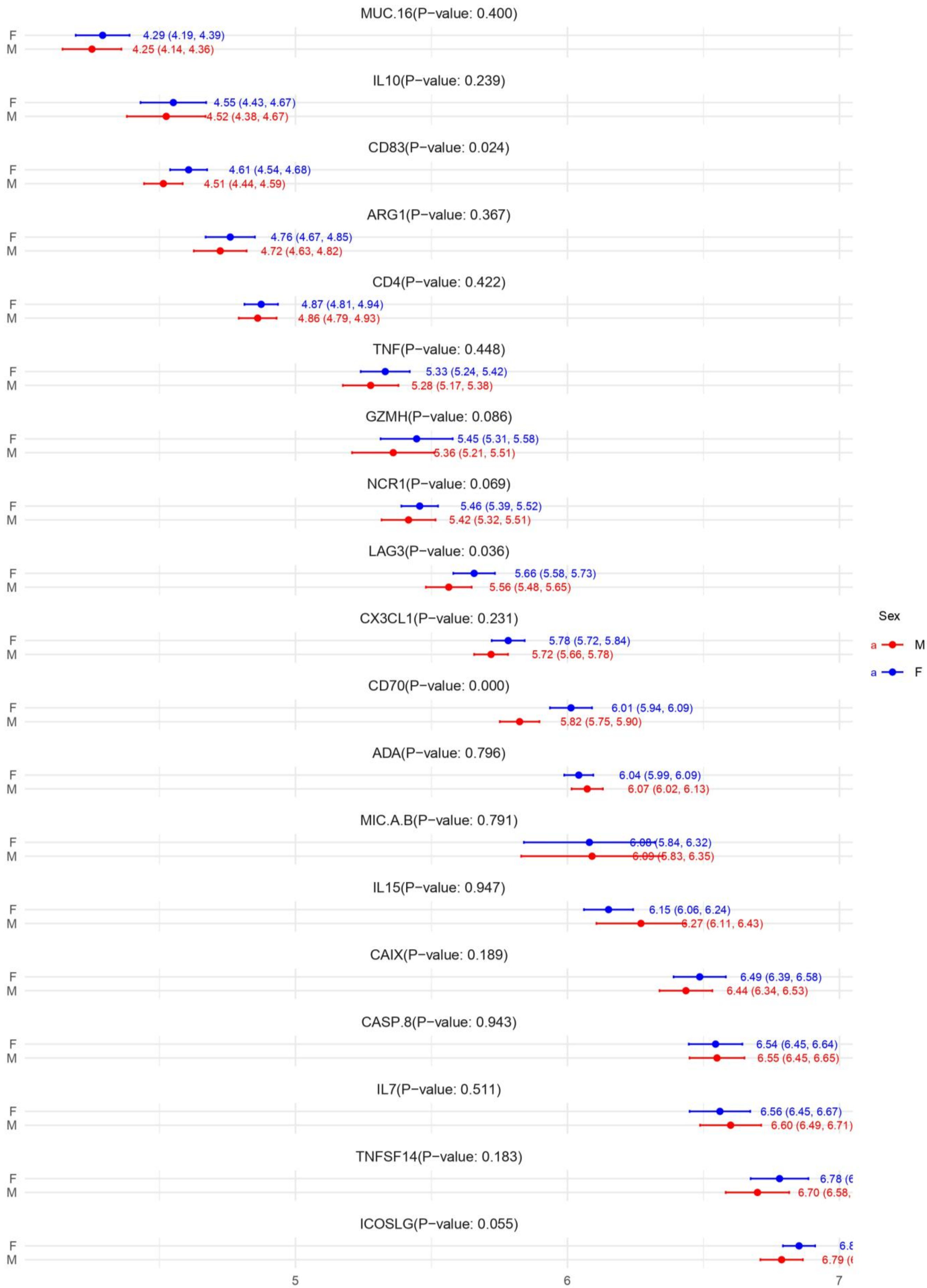

MUC.16(P−value: 0.400)
F
4.29 (4.19, 4.39)
M
4.25 (4.14, 4.36)
IL10(P−value: 0.239)
F
4.55 (4.43, 4.67)
M
4.52 (4.38, 4.67)
CD83(P−value: 0.024)
F
4.61 (4.54, 4.68)
M
4.51 (4.44, 4.59)
ARG1(P−value: 0.367)
F
4.76 (4.67, 4.85)
M
4.72 (4.63, 4.82)
CD4(P−value: 0.422)
F
4.87 (4.81, 4.94)
M
4.86 (4.79, 4.93)
TNF(P−value: 0.448)
F
5.33 (5.24, 5.42)
M
5.28 (5.17, 5.38)
GZMH(P−value: 0.086)
F
5.45 (5.31, 5.58)
M
5.36 (5.21, 5.51)
NCR1(P−value: 0.069)
F
5.46 (5.39, 5.52)
M
5.42 (5.32, 5.51)
LAG3(P−value: 0.036)
F
5.66 (5.58, 5.73)
M
5.56 (5.48, 5.65)
CX3CL1(P−value: 0.231)
F
5.78 (5.72, 5.84)
M
5.72 (5.66, 5.78)
CD70(P−value: 0.000)
F
6.01 (5.94, 6.09)
M
5.82 (5.75, 5.90)
ADA(P−value: 0.796)
F
6.04 (5.99, 6.09)
M
6.07 (6.02, 6.13)
MIC.A.B(P−value: 0.791)
F
6.08 (5.84, 6.32)
M
6.09 (5.83, 6.35)
IL15(P−value: 0.947)
F
6.15 (6.06, 6.24)
M
6.27 (6.11, 6.43)
CAIX(P−value: 0.189)
F
6.49 (6.39, 6.58)
M
6.44 (6.34, 6.53)
CASP.8(P−value: 0.943)
F
6.54 (6.45, 6.64)
M
6.55 (6.45, 6.65)
IL7(P−value: 0.511)
F
6.56 (6.45, 6.67)
M
6.60 (6.49, 6.71)
TNFSF14(P−value: 0.183)
F
6.78 (6
M
6.70 (6.58,
ICOSLG(P−value: 0.055)
F
6.8
M
6.79 (6
5
6
7
Sex
M
F

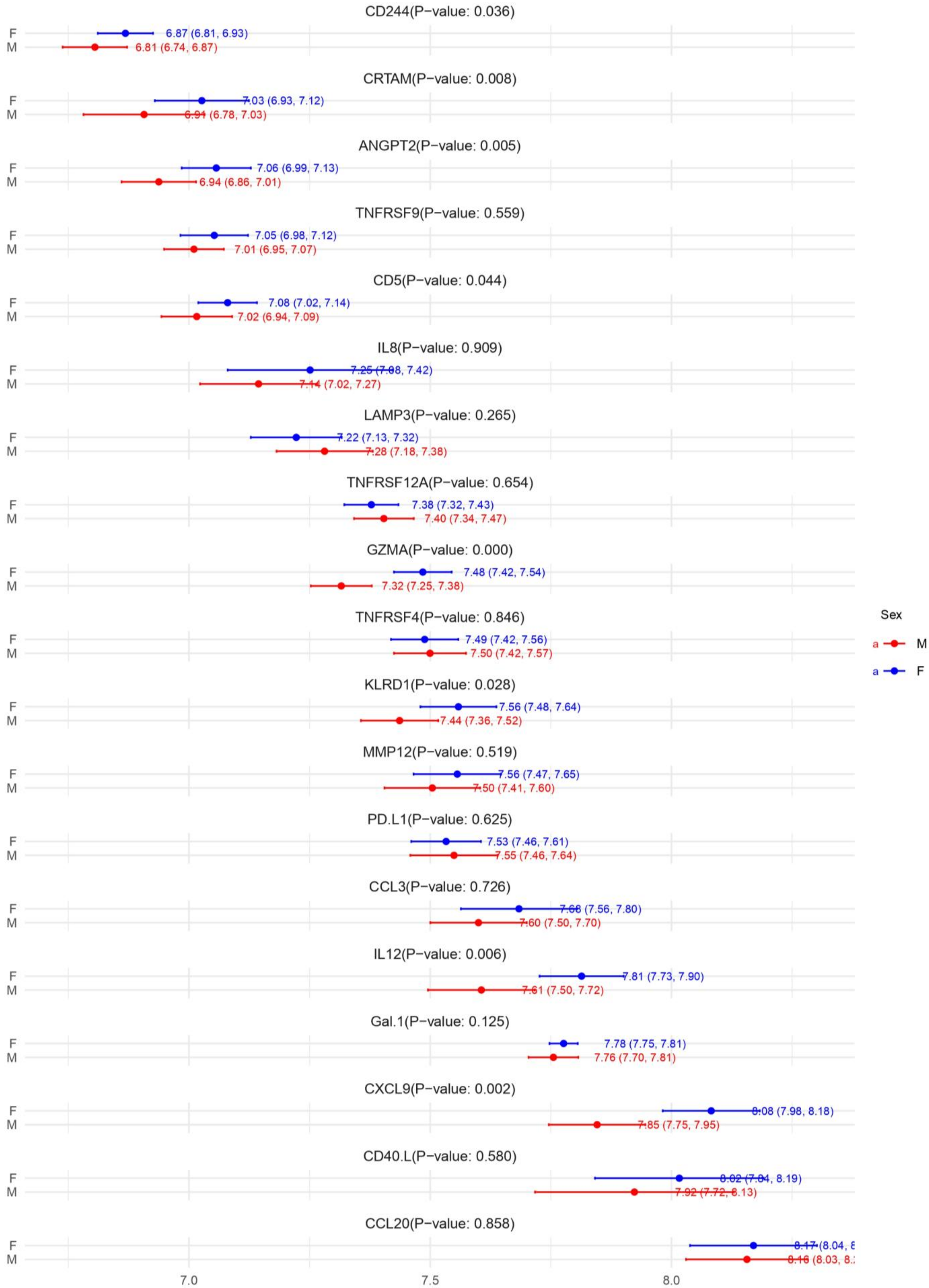

CD244(P−value: 0.036)
F 6.87 (6.81, 6.93)
M 6.81 (6.74, 6.87)
CRTAM(P−value: 0.008)
F 7.03 (6.93, 7.12)
M 6.91 (6.78, 7.03)
ANGPT2(P−value: 0.005)
F 7.06 (6.99, 7.13)
M 6.94 (6.86, 7.01)
TNFRSF9(P−value: 0.559)
F 7.05 (6.98, 7.12)
M 7.01 (6.95, 7.07)
CD5(P−value: 0.044)
F 7.08 (7.02, 7.14)
M 7.02 (6.94, 7.09)
IL8(P−value: 0.909)
F 7.25 (7.08, 7.42)
M 7.14 (7.02, 7.27)
LAMP3(P−value: 0.265)
F 7.22 (7.13, 7.32)
M 7.28 (7.18, 7.38)
TNFRSF12A(P−value: 0.654)
F 7.38 (7.32, 7.43)
M 7.40 (7.34, 7.47)
GZMA(P−value: 0.000)
F 7.48 (7.42, 7.54)
M 7.32 (7.25, 7.38)
TNFRSF4(P−value: 0.846)
F 7.49 (7.42, 7.56)
M 7.50 (7.42, 7.57)
KLRD1(P−value: 0.028)
F 7.56 (7.48, 7.64)
M 7.44 (7.36, 7.52)
MMP12(P−value: 0.519)
F 7.56 (7.47, 7.65)
M 7.50 (7.41, 7.60)
PD.L1(P−value: 0.625)
F 7.53 (7.46, 7.61)
M 7.55 (7.46, 7.64)
CCL3(P−value: 0.726)
F 7.68 (7.56, 7.80)
M 7.60 (7.50, 7.70)
IL12(P−value: 0.006)
F 7.81 (7.73, 7.90)
M 7.61 (7.50, 7.72)
Gal.1(P−value: 0.125)
F 7.78 (7.75, 7.81)
M 7.76 (7.70, 7.81)
CXCL9(P−value: 0.002)
F 8.08 (7.98, 8.18)
M 7.85 (7.75, 7.95)
CD40.L(P−value: 0.580)
F 8.02 (7.84, 8.19)
M 7.92 (7.72, 8.13)
CCL20(P−value: 0.858)
F 8.17 (8.04, 8
M 8.16 (8.03, 8.
7.0
7.5
8.0
Sex
M
F

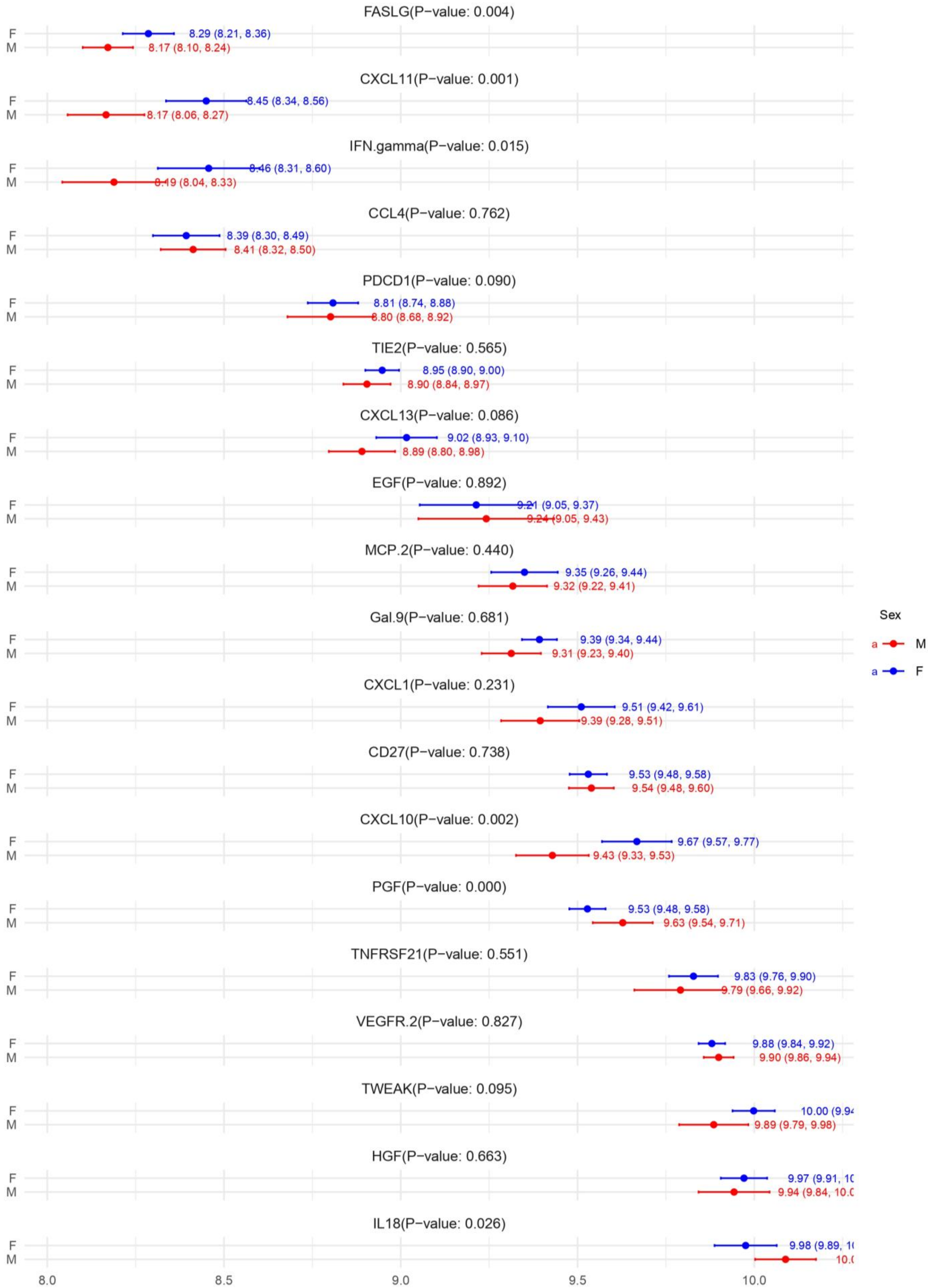

FASLG(P−value: 0.004)
F
M
8.29 (8.21, 8.36)
8.17 (8.10, 8.24)
CXCL11(P−value: 0.001)
8.45 (8.34, 8.56)
8.17 (8.06, 8.27)
IFN.gamma(P−value: 0.015)
8.46 (8.31, 8.60)
8.19 (8.04, 8.33)
CCL4(P−value: 0.762)
8.39 (8.30, 8.49)
8.41 (8.32, 8.50)
PDCD1(P−value: 0.090)
8.81 (8.74, 8.88)
8.80 (8.68, 8.92)
TIE2(P−value: 0.565)
8.95 (8.90, 9.00)
8.90 (8.84, 8.97)
CXCL13(P−value: 0.086)
9.02 (8.93, 9.10)
8.89 (8.80, 8.98)
EGF(P−value: 0.892)
9.21 (9.05, 9.37)
9.24 (9.05, 9.43)
MCP.2(P−value: 0.440)
9.35 (9.26, 9.44)
9.32 (9.22, 9.41)
Gal.9(P−value: 0.681)
9.39 (9.34, 9.44)
9.31 (9.23, 9.40)
CXCL1(P−value: 0.231)
9.51 (9.42, 9.61)
9.39 (9.28, 9.51)
CD27(P−value: 0.738)
9.53 (9.48, 9.58)
9.54 (9.48, 9.60)
CXCL10(P−value: 0.002)
9.67 (9.57, 9.77)
9.43 (9.33, 9.53)
PGF(P−value: 0.000)
9.53 (9.48, 9.58)
9.63 (9.54, 9.71)
TNFRSF21(P−value: 0.551)
9.83 (9.76, 9.90)
9.79 (9.66, 9.92)
VEGFR.2(P−value: 0.827)
9.88 (9.84, 9.92)
9.90 (9.86, 9.94)
TWEAK(P−value: 0.095)
10.00 (9.9
9.89 (9.79, 9.98)
HGF(P−value: 0.663)
9.97 (9.91, 1
9.94 (9.84, 10.
IL18(P−value: 0.026)
9.98 (9.89, 1
10.
8.0
8.5
9.0
9.5
10.0
Sex
M
F

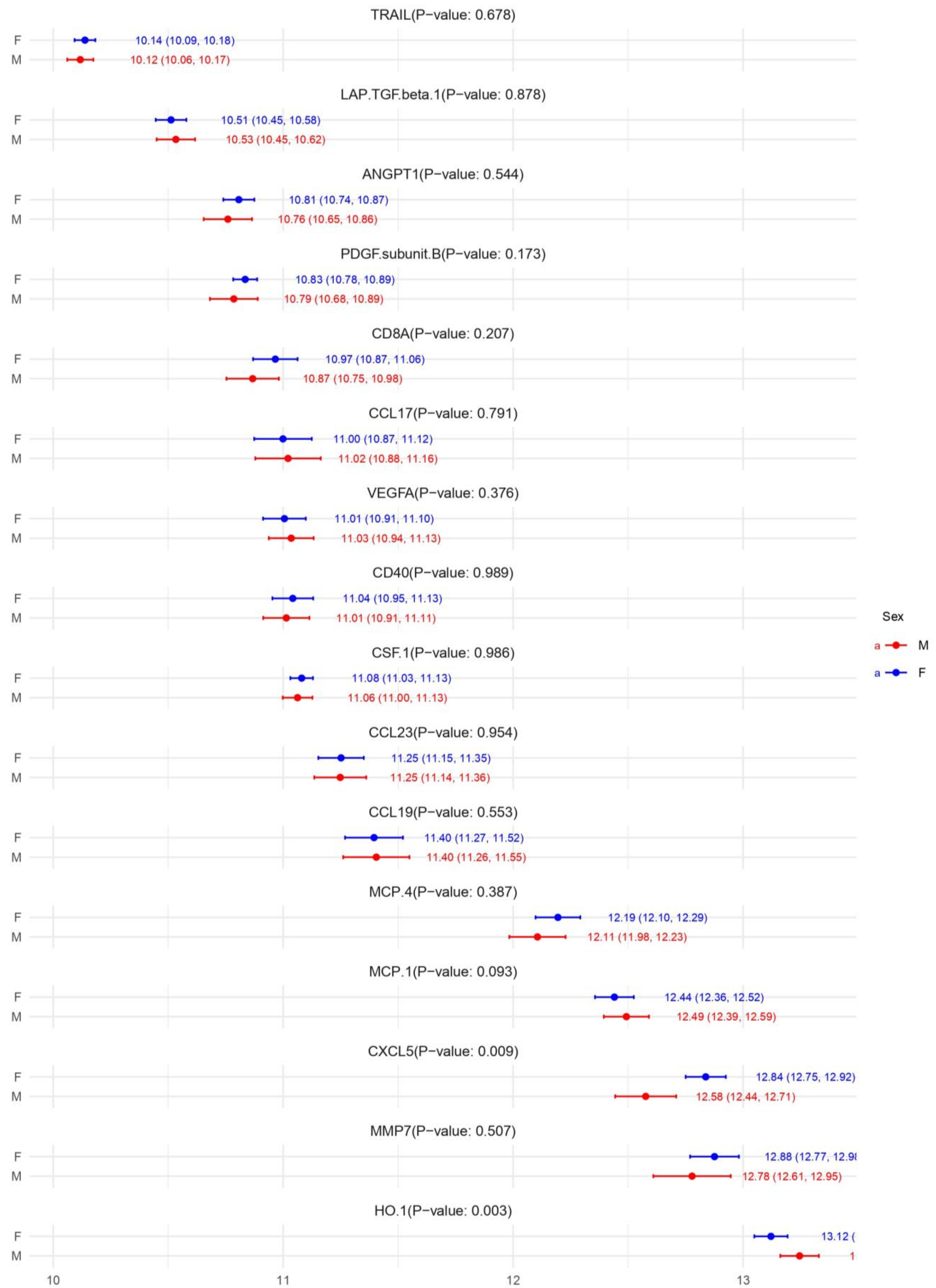

*Adjusted for batch, age at recruitment and country. F = female, M = male. The p-value was obtained using the Mann-Whitney U test because the 92 biomarkers do not follow a normal distribution.

Figure S8: Mean and 95% CI of Biomarkers by Smoking Status, among controls *

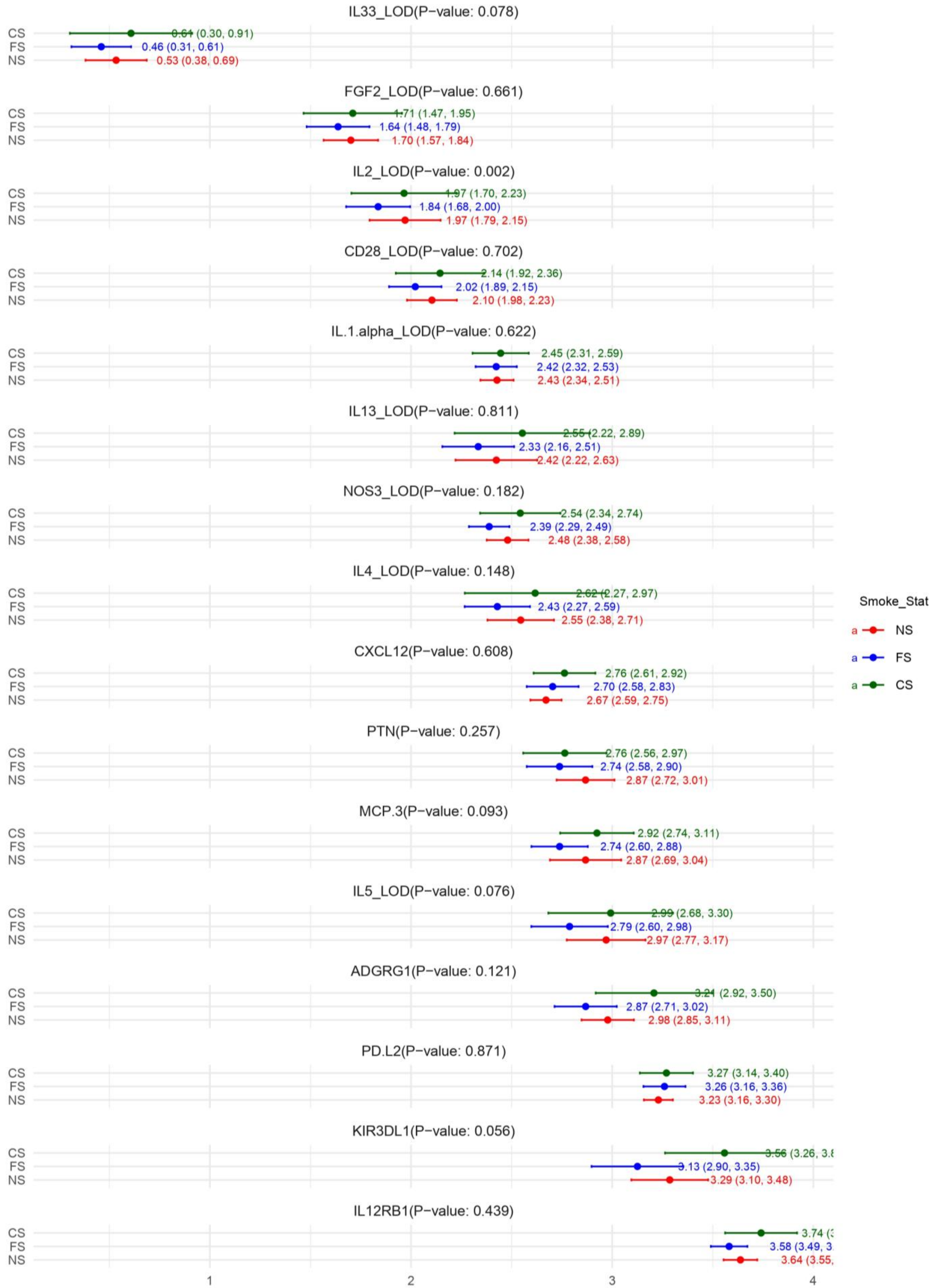

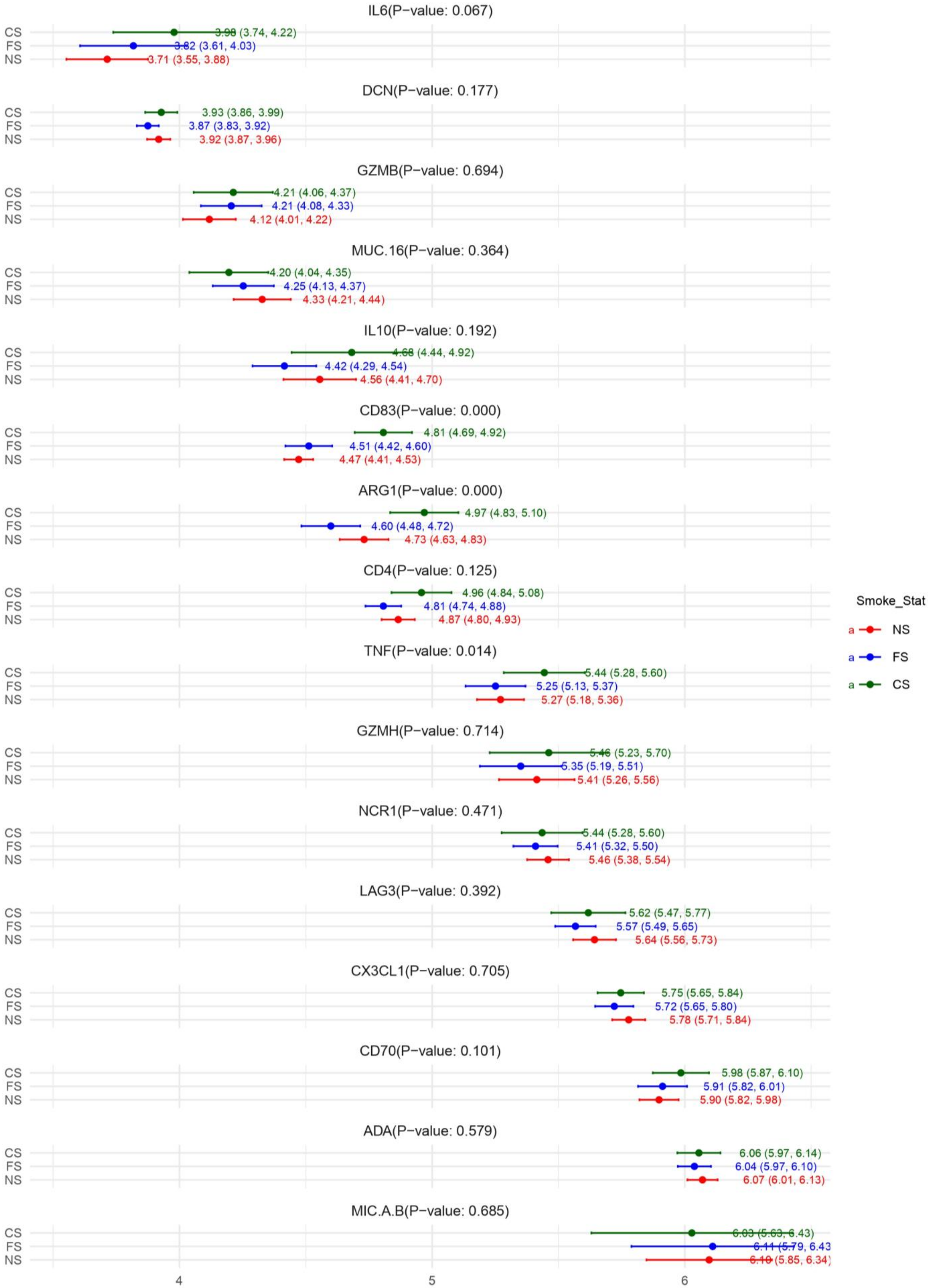
IL6(P-value: 0.067)
CS 3.98 (3.74, 4.22)
FS 3.82 (3.61, 4.03)
NS 3.71 (3.55, 3.88)
DCN(P-value: 0.177)
CS 3.93 (3.86, 3.99)
FS 3.87 (3.83, 3.92)
NS 3.92 (3.87, 3.96)
GZMB(P-value: 0.694)
CS 4.21 (4.06, 4.37)
FS 4.21 (4.08, 4.33)
NS 4.12 (4.01, 4.22)
MUC.16(P-value: 0.364)
CS 4.20 (4.04, 4.35)
FS 4.25 (4.13, 4.37)
NS 4.33 (4.21, 4.44)
IL10(P-value: 0.192)
CS 4.68 (4.44, 4.92)
FS 4.42 (4.29, 4.54)
NS 4.56 (4.41, 4.70)
CD83(P-value: 0.000)
CS 4.81 (4.69, 4.92)
FS 4.51 (4.42, 4.60)
NS 4.47 (4.41, 4.53)
ARG1(P-value: 0.000)
CS 4.97 (4.83, 5.10)
FS 4.60 (4.48, 4.72)
NS 4.73 (4.63, 4.83)
CD4(P-value: 0.125)
CS 4.96 (4.84, 5.08)
FS 4.81 (4.74, 4.88)
NS 4.87 (4.80, 4.93)
TNF(P-value: 0.014)
CS 5.44 (5.28, 5.60)
FS 5.25 (5.13, 5.37)
NS 5.27 (5.18, 5.36)
GZMH(P-value: 0.714)
CS 5.46 (5.23, 5.70)
FS 5.35 (5.19, 5.51)
NS 5.41 (5.26, 5.56)
NCR1(P-value: 0.471)
CS 5.44 (5.28, 5.60)
FS 5.41 (5.32, 5.50)
NS 5.46 (5.38, 5.54)
LAG3(P-value: 0.392)
CS 5.62 (5.47, 5.77)
FS 5.57 (5.49, 5.65)
NS 5.64 (5.56, 5.73)
CX3CL1(P-value: 0.705)
CS 5.75 (5.65, 5.84)
FS 5.72 (5.65, 5.80)
NS 5.78 (5.71, 5.84)
CD70(P-value: 0.101)
CS 5.98 (5.87, 6.10)
FS 5.91 (5.82, 6.01)
NS 5.90 (5.82, 5.98)
ADA(P-value: 0.579)
CS 6.06 (5.97, 6.14)
FS 6.04 (5.97, 6.10)
NS 6.07 (6.01, 6.13)
MIC.A.B(P-value: 0.685)
CS 6.03 (5.63, 6.43)
FS 6.11 (5.79, 6.43
NS 6.10 (5.85, 6.34)
4
5
6
Smoke_Stat
a NS
a FS
a CS

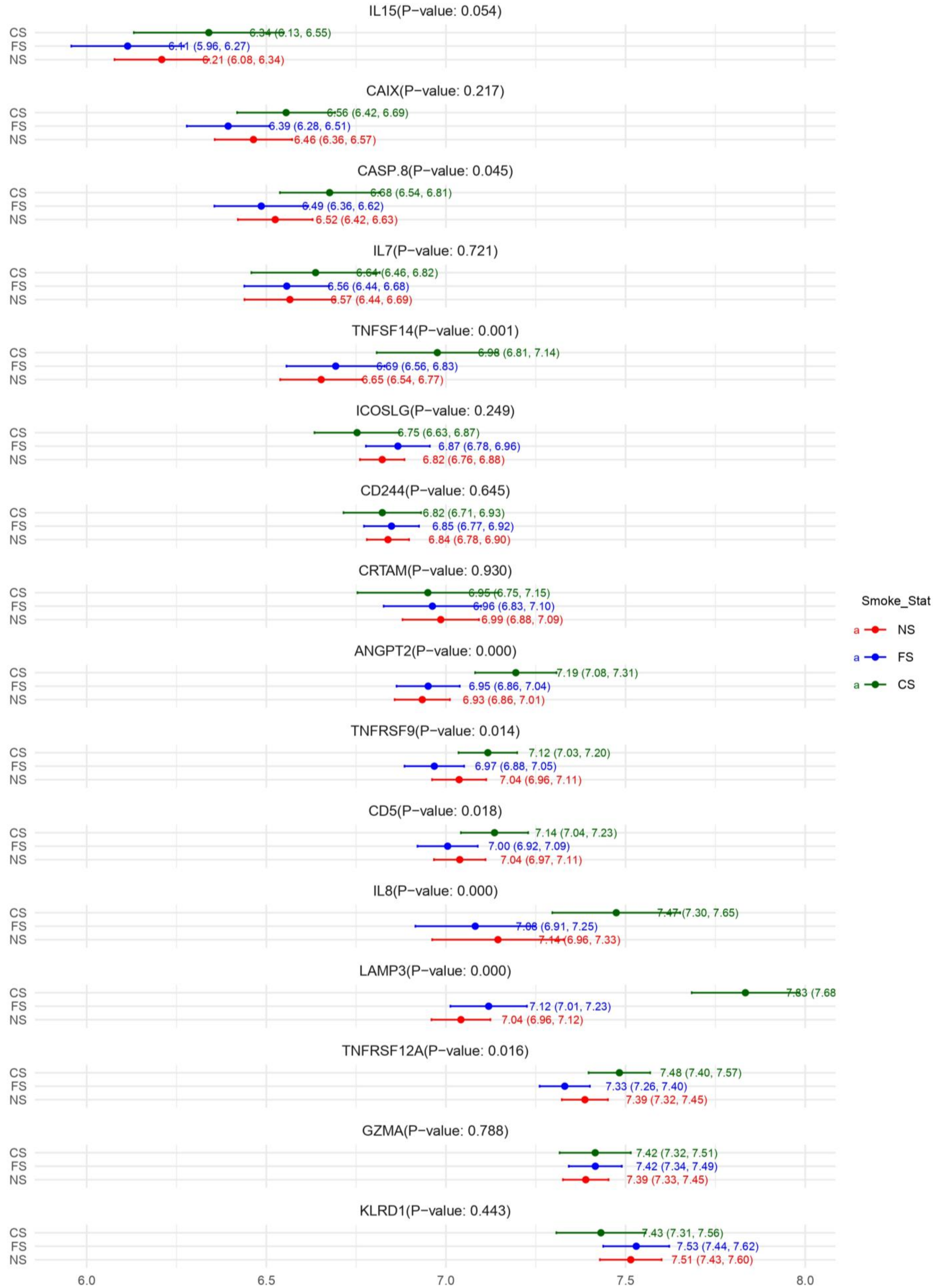

IL15(P−value: 0.054)
CS 6.34 (6.13, 6.55)
FS 6.11 (5.96, 6.27)
NS 6.21 (6.08, 6.34)
CAIX(P−value: 0.217)
CS 6.56 (6.42, 6.69)
FS 6.39 (6.28, 6.51)
NS 6.46 (6.36, 6.57)
CASP.8(P−value: 0.045)
CS 6.68 (6.54, 6.81)
FS 6.49 (6.36, 6.62)
NS 6.52 (6.42, 6.63)
IL7(P−value: 0.721)
CS 6.64 (6.46, 6.82)
FS 6.56 (6.44, 6.68)
NS 6.57 (6.44, 6.69)
TNFSF14(P−value: 0.001)
CS 6.98 (6.81, 7.14)
FS 6.69 (6.56, 6.83)
NS 6.65 (6.54, 6.77)
ICOSLG(P−value: 0.249)
CS 6.75 (6.63, 6.87)
FS 6.87 (6.78, 6.96)
NS 6.82 (6.76, 6.88)
CD244(P−value: 0.645)
CS 6.82 (6.71, 6.93)
FS 6.85 (6.77, 6.92)
NS 6.84 (6.78, 6.90)
CRTAM(P−value: 0.930)
CS 6.95 (6.75, 7.15)
FS 6.96 (6.83, 7.10)
NS 6.99 (6.88, 7.09)
ANGPT2(P−value: 0.000)
CS 7.19 (7.08, 7.31)
FS 6.95 (6.86, 7.04)
NS 6.93 (6.86, 7.01)
TNFRSF9(P−value: 0.014)
CS 7.12 (7.03, 7.20)
FS 6.97 (6.88, 7.05)
NS 7.04 (6.96, 7.11)
CD5(P−value: 0.018)
CS 7.14 (7.04, 7.23)
FS 7.00 (6.92, 7.09)
NS 7.04 (6.97, 7.11)
IL8(P−value: 0.000)
CS 7.47 (7.30, 7.65)
FS 7.08 (6.91, 7.25)
NS 7.14 (6.96, 7.33)
LAMP3(P−value: 0.000)
CS 7.83 (7.68
FS 7.12 (7.01, 7.23)
NS 7.04 (6.96, 7.12)
TNFRSF12A(P−value: 0.016)
CS 7.48 (7.40, 7.57)
FS 7.33 (7.26, 7.40)
NS 7.39 (7.32, 7.45)
GZMA(P−value: 0.788)
CS 7.42 (7.32, 7.51)
FS 7.42 (7.34, 7.49)
NS 7.39 (7.33, 7.45)
KLRD1(P−value: 0.443)
CS 7.43 (7.31, 7.56)
FS 7.53 (7.44, 7.62)
NS 7.51 (7.43, 7.60)
6.0
6.5
7.0
7.5
8.0
Smoke_Stat
NS
FS
CS

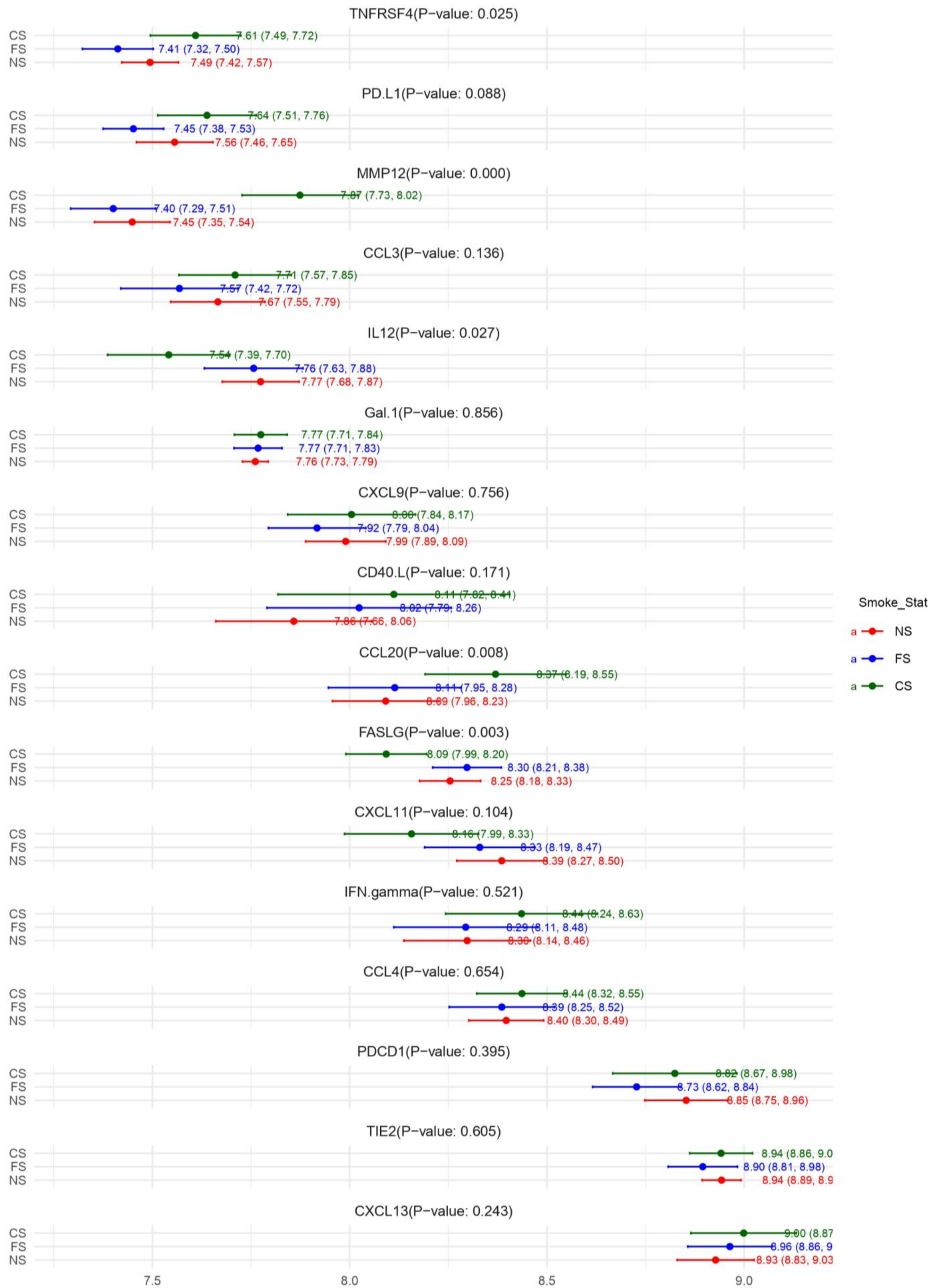
TNFRSF4(P−value: 0.025)
CS 7.61 (7.49, 7.72)
FS 7.41 (7.32, 7.50)
NS 7.49 (7.42, 7.57)
PD.L1(P−value: 0.088)
CS 7.64 (7.51, 7.76)
FS 7.45 (7.38, 7.53)
NS 7.56 (7.46, 7.65)
MMP12(P−value: 0.000)
CS 7.87 (7.73, 8.02)
FS 7.40 (7.29, 7.51)
NS 7.45 (7.35, 7.54)
CCL3(P−value: 0.136)
CS 7.71 (7.57, 7.85)
FS 7.57 (7.42, 7.72)
NS 7.67 (7.55, 7.79)
IL12(P−value: 0.027)
CS 7.54 (7.39, 7.70)
FS 7.76 (7.63, 7.88)
NS 7.77 (7.68, 7.87)
Gal.1(P−value: 0.856)
CS 7.77 (7.71, 7.84)
FS 7.77 (7.71, 7.83)
NS 7.76 (7.73, 7.79)
CXCL9(P−value: 0.756)
CS 8.00 (7.84, 8.17)
FS 7.92 (7.79, 8.04)
NS 7.99 (7.89, 8.09)
CD40.L(P−value: 0.171)
CS 8.11 (7.82, 8.41)
FS 8.02 (7.79, 8.26)
NS 7.86 (7.66, 8.06)
CCL20(P−value: 0.008)
CS 8.37 (8.19, 8.55)
FS 8.11 (7.95, 8.28)
NS 8.09 (7.96, 8.23)
FASLG(P−value: 0.003)
CS 8.09 (7.99, 8.20)
FS 8.30 (8.21, 8.38)
NS 8.25 (8.18, 8.33)
CXCL11(P−value: 0.104)
CS 8.16 (7.99, 8.33)
FS 8.33 (8.19, 8.47)
NS 8.39 (8.27, 8.50)
IFN.gamma(P−value: 0.521)
CS 8.44 (8.24, 8.63)
FS 8.29 (8.11, 8.48)
NS 8.30 (8.14, 8.46)
CCL4(P−value: 0.654)
CS 8.44 (8.32, 8.55)
FS 8.39 (8.25, 8.52)
NS 8.40 (8.30, 8.49)
PDCD1(P−value: 0.395)
CS 8.82 (8.67, 8.98)
FS 8.73 (8.62, 8.84)
NS 8.85 (8.75, 8.96)
TIE2(P−value: 0.605)
CS 8.94 (8.86, 9.0
FS 8.90 (8.81, 8.98)
NS 8.94 (8.89, 8.9
CXCL13(P−value: 0.243)
CS 9.00 (8.87
FS 8.96 (8.86, 9
NS 8.93 (8.83, 9.03
7.5
8.0
8.5
9.0
Smoke_Stat
NS
FS
CS

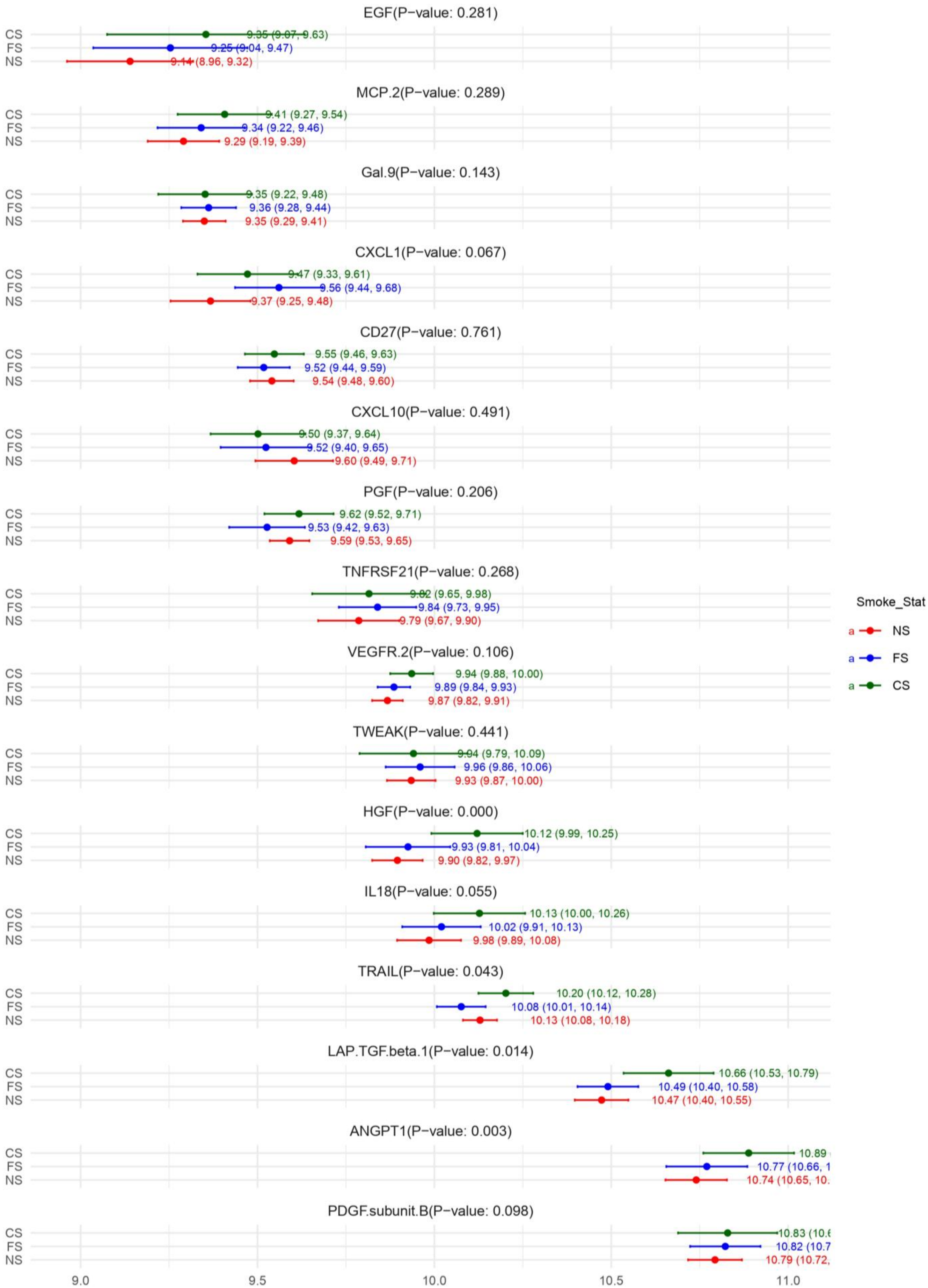

EGF(P−value: 0.281)
CS 9.35 (9.07, 9.63)
FS 9.25 (9.04, 9.47)
NS 9.14 (8.96, 9.32)
MCP.2(P−value: 0.289)
CS 9.41 (9.27, 9.54)
FS 9.34 (9.22, 9.46)
NS 9.29 (9.19, 9.39)
Gal.9(P−value: 0.143)
CS 9.35 (9.22, 9.48)
FS 9.36 (9.28, 9.44)
NS 9.35 (9.29, 9.41)
CXCL1(P−value: 0.067)
CS 9.47 (9.33, 9.61)
FS 9.56 (9.44, 9.68)
NS 9.37 (9.25, 9.48)
CD27(P−value: 0.761)
CS 9.55 (9.46, 9.63)
FS 9.52 (9.44, 9.59)
NS 9.54 (9.48, 9.60)
CXCL10(P−value: 0.491)
CS 9.50 (9.37, 9.64)
FS 9.52 (9.40, 9.65)
NS 9.60 (9.49, 9.71)
PGF(P−value: 0.206)
CS 9.62 (9.52, 9.71)
FS 9.53 (9.42, 9.63)
NS 9.59 (9.53, 9.65)
TNFRSF21(P−value: 0.268)
CS 9.82 (9.65, 9.98)
FS 9.84 (9.73, 9.95)
NS 9.79 (9.67, 9.90)
VEGFR.2(P−value: 0.106)
CS 9.94 (9.88, 10.00)
FS 9.89 (9.84, 9.93)
NS 9.87 (9.82, 9.91)
TWEAK(P−value: 0.441)
CS 9.94 (9.79, 10.09)
FS 9.96 (9.86, 10.06)
NS 9.93 (9.87, 10.00)
HGF(P−value: 0.000)
CS 10.12 (9.99, 10.25)
FS 9.93 (9.81, 10.04)
NS 9.90 (9.82, 9.97)
IL18(P−value: 0.055)
CS 10.13 (10.00, 10.26)
FS 10.02 (9.91, 10.13)
NS 9.98 (9.89, 10.08)
TRAIL(P−value: 0.043)
CS 10.20 (10.12, 10.28)
FS 10.08 (10.01, 10.14)
NS 10.13 (10.08, 10.18)
LAP.TGF.beta.1(P−value: 0.014)
CS 10.66 (10.53, 10.79)
FS 10.49 (10.40, 10.58)
NS 10.47 (10.40, 10.55)
ANGPT1(P−value: 0.003)
CS 10.89
FS 10.77 (10.66, 1
NS 10.74 (10.65, 10.
PDGF.subunit.B(P−value: 0.098)
CS 10.83 (10.6
FS 10.82 (10.7
NS 10.79 (10.72,
9.0
9.5
10.0
10.5
11.0
Smoke_Stat
NS
FS
CS

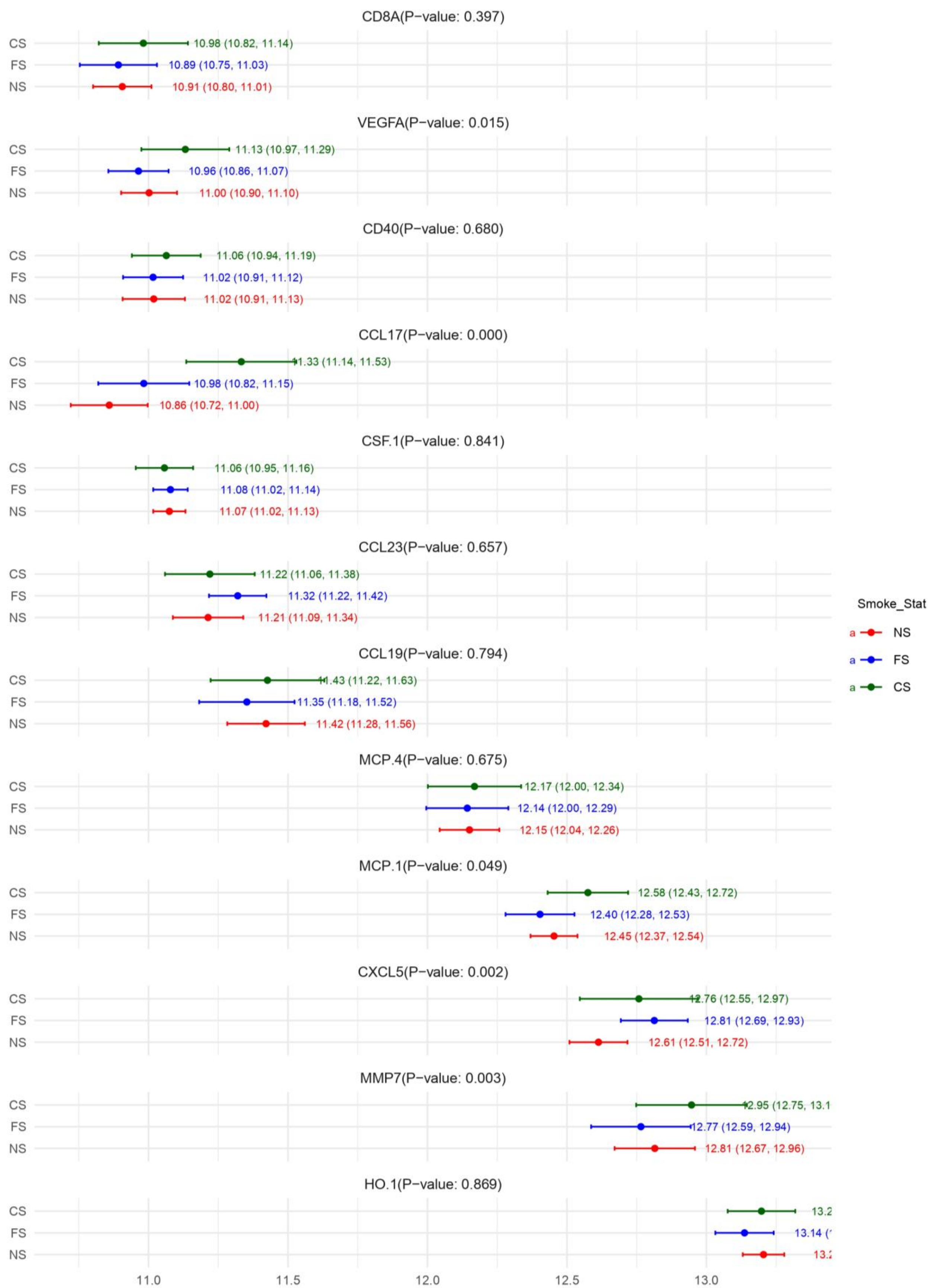

*Adjusted for batch, age at recruitment, sex and country. NS = never smoker, FS = former smoker, CS = current smoker. The p-value was obtained using the Mann-Whitney U test because the 92 biomarkers do not follow a normal distribution.

Figure S9: Mean and 95% CI of Biomarkers by Alcohol Consumption, among controls *

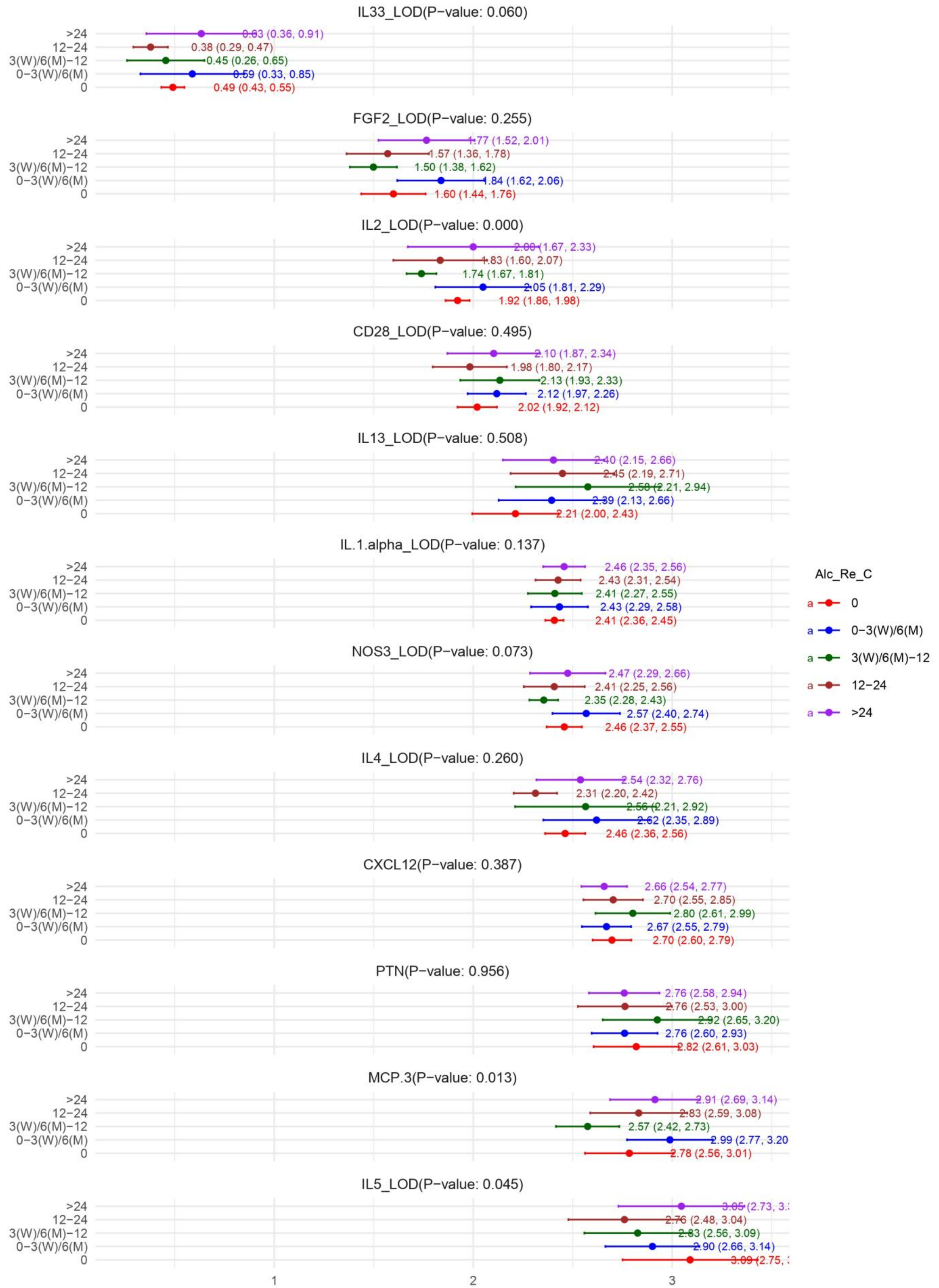

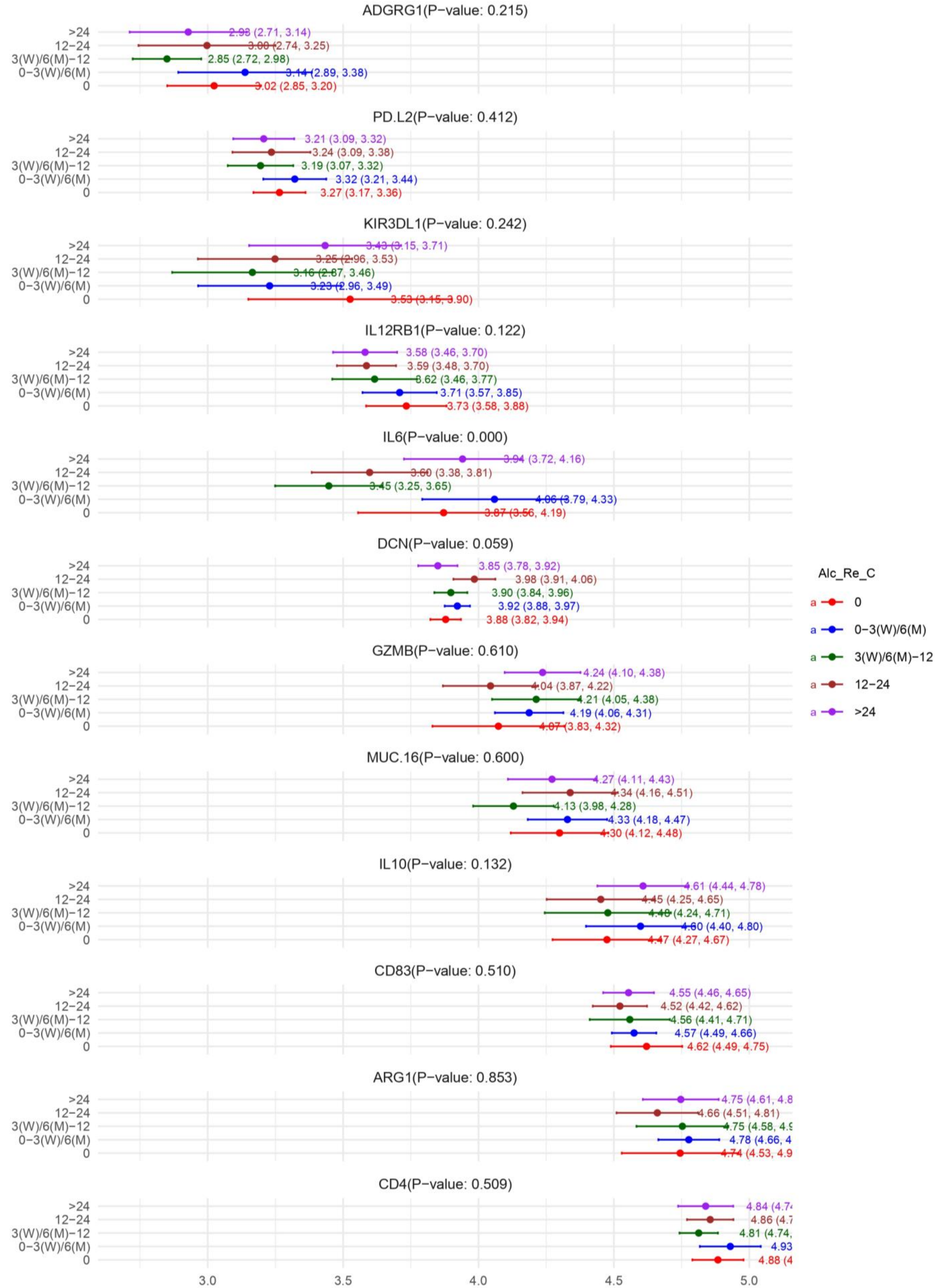

ADGRG1(P−value: 0.215)
2.93 (2.71, 3.14)
3.00 (2.74, 3.25)
2.85 (2.72, 2.98)
3.14 (2.89, 3.38)
3.02 (2.85, 3.20)
PD.L2(P−value: 0.412)
3.21 (3.09, 3.32)
3.24 (3.09, 3.38)
3.19 (3.07, 3.32)
3.32 (3.21, 3.44)
3.27 (3.17, 3.36)
KIR3DL1(P−value: 0.242)
3.43 (3.15, 3.71)
3.25 (2.96, 3.53)
3.16 (2.87, 3.46)
3.23 (2.96, 3.49)
3.53 (3.15, 3.90)
IL12RB1(P−value: 0.122)
3.58 (3.46, 3.70)
3.59 (3.48, 3.70)
3.62 (3.46, 3.77)
3.71 (3.57, 3.85)
3.73 (3.58, 3.88)
IL6(P−value: 0.000)
3.94 (3.72, 4.16)
3.60 (3.38, 3.81)
3.45 (3.25, 3.65)
4.06 (3.79, 4.33)
3.87 (3.56, 4.19)
DCN(P−value: 0.059)
3.85 (3.78, 3.92)
3.98 (3.91, 4.06)
3.90 (3.84, 3.96)
3.92 (3.88, 3.97)
3.88 (3.82, 3.94)
GZMB(P−value: 0.610)
4.24 (4.10, 4.38)
4.04 (3.87, 4.22)
4.21 (4.05, 4.38)
4.19 (4.06, 4.31)
4.07 (3.83, 4.32)
MUC.16(P−value: 0.600)
4.27 (4.11, 4.43)
4.34 (4.16, 4.51)
4.13 (3.98, 4.28)
4.33 (4.18, 4.47)
4.30 (4.12, 4.48)
IL10(P−value: 0.132)
4.61 (4.44, 4.78)
4.45 (4.25, 4.65)
4.48 (4.24, 4.71)
4.60 (4.40, 4.80)
4.47 (4.27, 4.67)
CD83(P−value: 0.510)
4.55 (4.46, 4.65)
4.52 (4.42, 4.62)
4.56 (4.41, 4.71)
4.57 (4.49, 4.66)
4.62 (4.49, 4.75)
ARG1(P−value: 0.853)
4.66 (4.51, 4.81)
CD4(P−value: 0.509)
>24
12−24
3(W)/6(M)−12
0−3(W)/6(M)
0
3.0
3.5
4.0
4.5
5.0
Alc_Re_C

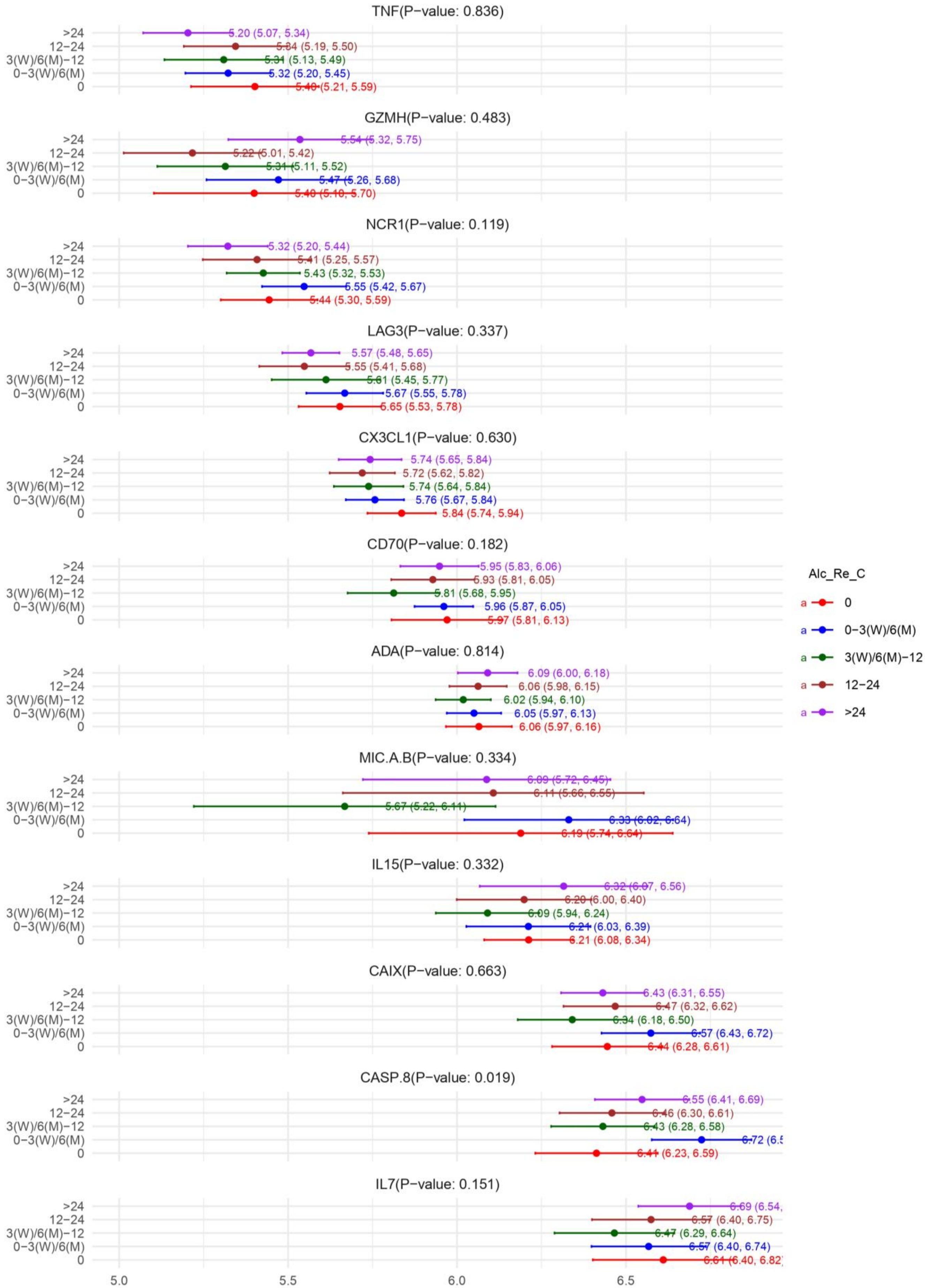
TNF(P−value: 0.836)
>24
12−24
3(W)/6(M)−12
0−3(W)/6(M)
0
5.20 (5.07, 5.34)
5.34 (5.19, 5.50)
5.31 (5.13, 5.49)
5.32 (5.20, 5.45)
5.40 (5.21, 5.59)
GZMH(P−value: 0.483)
5.54 (5.32, 5.75)
5.22 (5.01, 5.42)
5.31 (5.11, 5.52)
5.47 (5.26, 5.68)
5.40 (5.10, 5.70)
NCR1(P−value: 0.119)
5.32 (5.20, 5.44)
5.41 (5.25, 5.57)
5.43 (5.32, 5.53)
5.55 (5.42, 5.67)
5.44 (5.30, 5.59)
LAG3(P−value: 0.337)
5.57 (5.48, 5.65)
5.55 (5.41, 5.68)
5.61 (5.45, 5.77)
5.67 (5.55, 5.78)
5.65 (5.53, 5.78)
CX3CL1(P−value: 0.630)
5.74 (5.65, 5.84)
5.72 (5.62, 5.82)
5.74 (5.64, 5.84)
5.76 (5.67, 5.84)
5.84 (5.74, 5.94)
CD70(P−value: 0.182)
5.95 (5.83, 6.06)
5.93 (5.81, 6.05)
5.81 (5.68, 5.95)
5.96 (5.87, 6.05)
5.97 (5.81, 6.13)
ADA(P−value: 0.814)
6.09 (6.00, 6.18)
6.06 (5.98, 6.15)
6.02 (5.94, 6.10)
6.05 (5.97, 6.13)
6.06 (5.97, 6.16)
MIC.A.B(P−value: 0.334)
6.09 (5.72, 6.45)
6.11 (5.66, 6.55)
5.67 (5.22, 6.11)
6.33 (6.02, 6.64)
6.19 (5.74, 6.64)
IL15(P−value: 0.332)
6.32 (6.07, 6.56)
6.20 (6.00, 6.40)
6.09 (5.94, 6.24)
6.21 (6.03, 6.39)
6.21 (6.08, 6.34)
CAIX(P−value: 0.663)
6.43 (6.31, 6.55)
6.47 (6.32, 6.62)
6.34 (6.18, 6.50)
6.57 (6.43, 6.72)
6.44 (6.28, 6.61)
CASP.8(P−value: 0.019)
6.55 (6.41, 6.69)
6.46 (6.30, 6.61)
6.43 (6.28, 6.58)
6.72 (6.5
6.41 (6.23, 6.59)
IL7(P−value: 0.151)
6.69 (6.54,
6.57 (6.40, 6.75)
6.47 (6.29, 6.64)
6.57 (6.40, 6.74)
6.61 (6.40, 6.82)
5.0
5.5
6.0
6.5
Alc_Re_C
0
0−3(W)/6(M)
3(W)/6(M)−12
12−24
>24

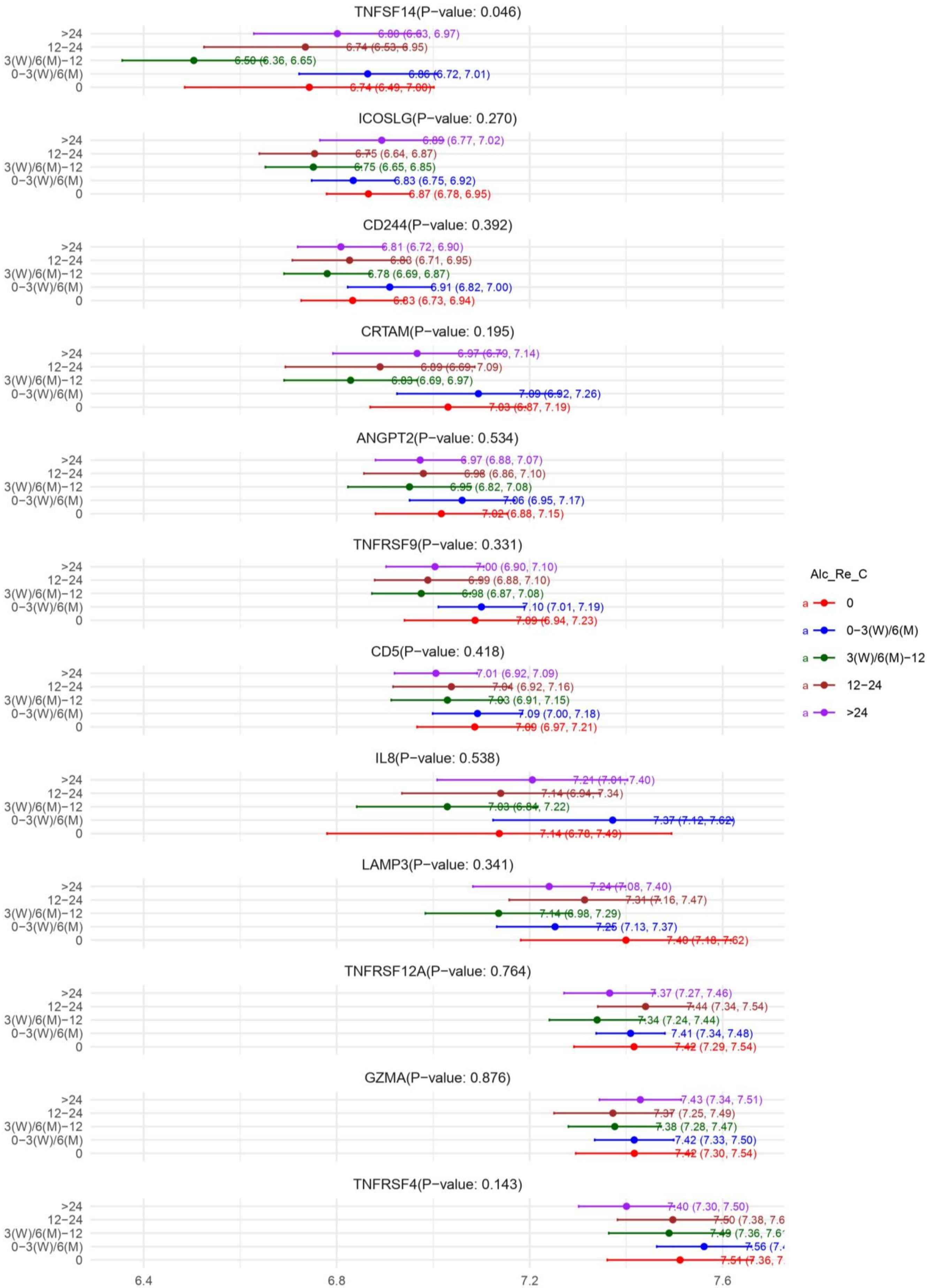

TNFSF14(P−value: 0.046)
>24
12−24
3(W)/6(M)−12
0−3(W)/6(M)
0
6.80 (6.63, 6.97)
6.74 (6.53, 6.95)
6.50 (6.36, 6.65)
6.86 (6.72, 7.01)
6.74 (6.49, 7.00)
ICOSLG(P−value: 0.270)
6.89 (6.77, 7.02)
6.75 (6.64, 6.87)
6.75 (6.65, 6.85)
6.83 (6.75, 6.92)
6.87 (6.78, 6.95)
CD244(P−value: 0.392)
6.81 (6.72, 6.90)
6.80 (6.71, 6.95)
6.78 (6.69, 6.87)
6.91 (6.82, 7.00)
6.83 (6.73, 6.94)
CRTAM(P−value: 0.195)
6.97 (6.79, 7.14)
6.89 (6.69, 7.09)
6.83 (6.69, 6.97)
7.09 (6.92, 7.26)
7.03 (6.87, 7.19)
ANGPT2(P−value: 0.534)
6.97 (6.88, 7.07)
6.98 (6.86, 7.10)
6.95 (6.82, 7.08)
7.06 (6.95, 7.17)
7.02 (6.88, 7.15)
TNFRSF9(P−value: 0.331)
7.00 (6.90, 7.10)
6.99 (6.88, 7.10)
6.98 (6.87, 7.08)
7.10 (7.01, 7.19)
7.09 (6.94, 7.23)
CD5(P−value: 0.418)
7.01 (6.92, 7.09)
7.04 (6.92, 7.16)
7.03 (6.91, 7.15)
7.09 (7.00, 7.18)
7.09 (6.97, 7.21)
IL8(P−value: 0.538)
7.21 (7.01, 7.40)
7.14 (6.94, 7.34)
7.03 (6.84, 7.22)
7.37 (7.12, 7.62)
7.14 (6.78, 7.49)
LAMP3(P−value: 0.341)
7.24 (7.08, 7.40)
7.31 (7.16, 7.47)
7.14 (6.98, 7.29)
7.25 (7.13, 7.37)
7.40 (7.18, 7.62)
TNFRSF12A(P−value: 0.764)
7.37 (7.27, 7.46)
7.44 (7.34, 7.54)
7.34 (7.24, 7.44)
7.41 (7.34, 7.48)
7.42 (7.29, 7.54)
GZMA(P−value: 0.876)
7.43 (7.34, 7.51)
7.37 (7.25, 7.49)
7.38 (7.28, 7.47)
7.42 (7.33, 7.50)
7.42 (7.30, 7.54)
TNFRSF4(P−value: 0.143)
7.40 (7.30, 7.50)
7.50 (7.38, 7.6
7.49 (7.36, 7.6
7.56 (7.4
7.51 (7.36, 7.
6.4
6.8
7.2
7.6
Alc_Re_C
0
0−3(W)/6(M)
3(W)/6(M)−12
12−24
>24

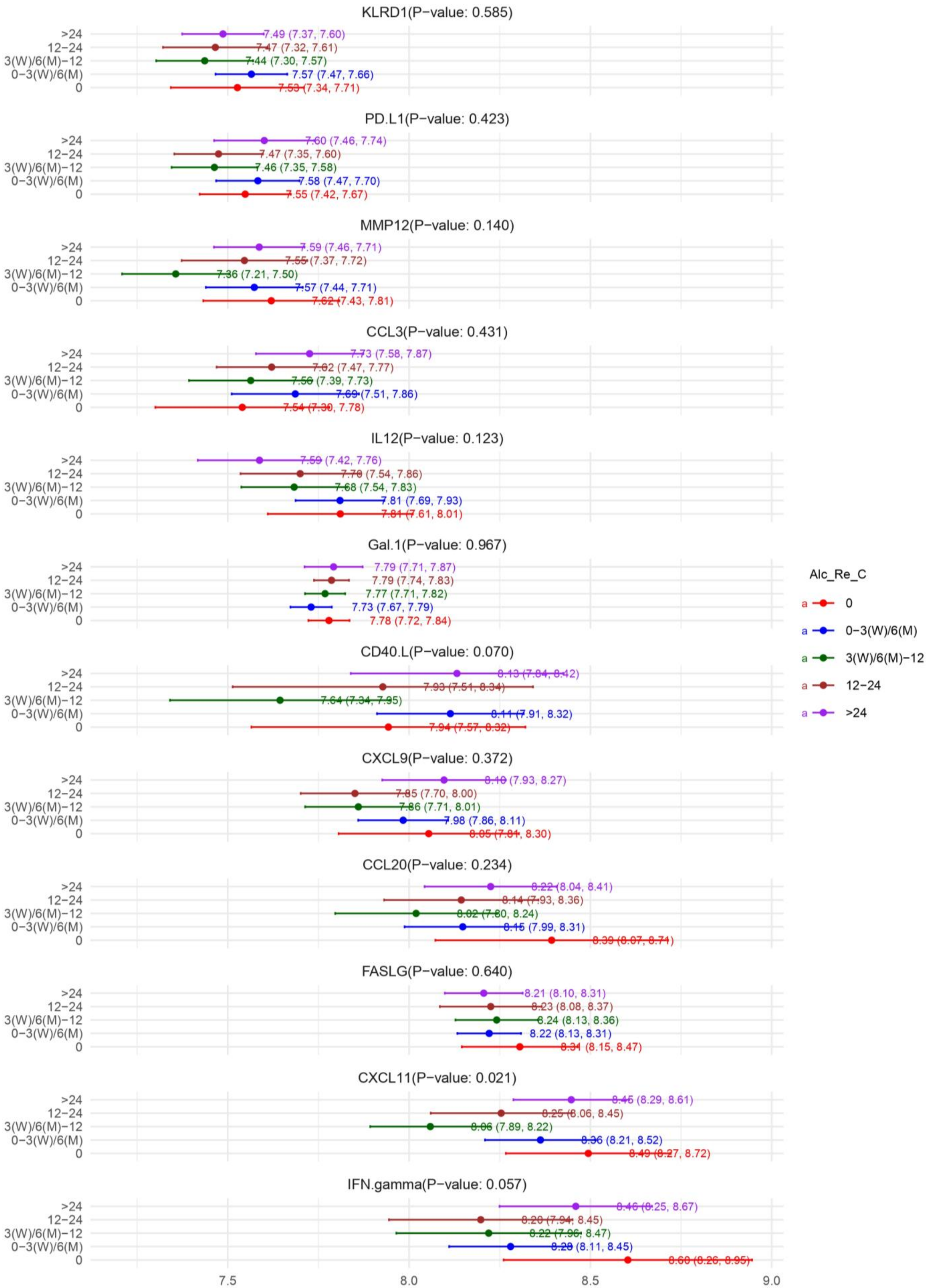

KLRD1(P−value: 0.585)
7.49 (7.37, 7.60)
7.47 (7.32, 7.61)
7.44 (7.30, 7.57)
7.57 (7.47, 7.66)
7.53 (7.34, 7.71)
PD.L1(P−value: 0.423)
7.60 (7.46, 7.74)
7.47 (7.35, 7.60)
7.46 (7.35, 7.58)
7.58 (7.47, 7.70)
7.55 (7.42, 7.67)
MMP12(P−value: 0.140)
7.59 (7.46, 7.71)
7.55 (7.37, 7.72)
7.36 (7.21, 7.50)
7.57 (7.44, 7.71)
7.62 (7.43, 7.81)
CCL3(P−value: 0.431)
7.73 (7.58, 7.87)
7.62 (7.47, 7.77)
7.56 (7.39, 7.73)
7.69 (7.51, 7.86)
7.54 (7.30, 7.78)
IL12(P−value: 0.123)
7.59 (7.42, 7.76)
7.70 (7.54, 7.86)
7.68 (7.54, 7.83)
7.81 (7.69, 7.93)
7.81 (7.61, 8.01)
Gal.1(P−value: 0.967)
7.79 (7.71, 7.87)
7.79 (7.74, 7.83)
7.77 (7.71, 7.82)
7.73 (7.67, 7.79)
7.78 (7.72, 7.84)
CD40.L(P−value: 0.070)
8.13 (7.84, 8.42)
7.93 (7.51, 8.34)
7.64 (7.34, 7.95)
8.11 (7.91, 8.32)
7.94 (7.57, 8.32)
CXCL9(P−value: 0.372)
8.10 (7.93, 8.27)
7.85 (7.70, 8.00)
7.86 (7.71, 8.01)
7.98 (7.86, 8.11)
8.05 (7.81, 8.30)
CCL20(P−value: 0.234)
8.22 (8.04, 8.41)
8.14 (7.93, 8.36)
8.02 (7.80, 8.24)
8.15 (7.99, 8.31)
8.39 (8.07, 8.71)
FASLG(P−value: 0.640)
8.21 (8.10, 8.31)
8.23 (8.08, 8.37)
8.24 (8.13, 8.36)
8.22 (8.13, 8.31)
8.31 (8.15, 8.47)
CXCL11(P−value: 0.021)
8.45 (8.29, 8.61)
8.25 (8.06, 8.45)
8.06 (7.89, 8.22)
8.36 (8.21, 8.52)
8.49 (8.27, 8.72)
IFN.gamma(P−value: 0.057)
8.46 (8.25, 8.67)
8.20 (7.94, 8.45)
8.22 (7.96, 8.47)
8.28 (8.11, 8.45)
8.60 (8.26, 8.95)
>24
12−24
3(W)/6(M)−12
0−3(W)/6(M)
0
7.5
8.0
8.5
9.0
Alc_Re_C
0
0−3(W)/6(M)
3(W)/6(M)−12
12−24
>24

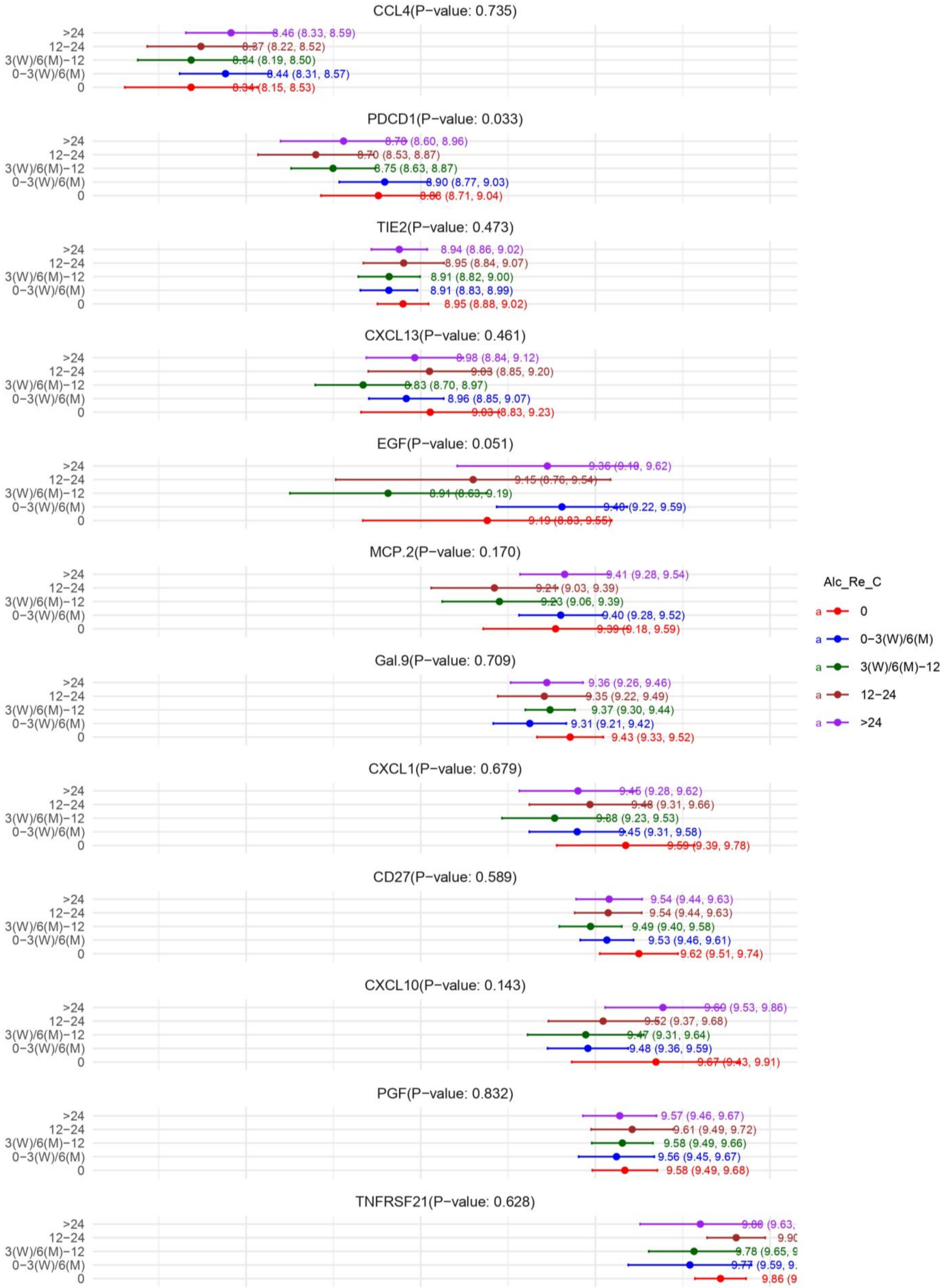

CCL4(P−value: 0.735)
PDCD1(P−value: 0.033)
TIE2(P−value: 0.473)
CXCL13(P−value: 0.461)
EGF(P−value: 0.051)
MCP.2(P−value: 0.170)
Gal.9(P−value: 0.709)
CXCL1(P−value: 0.679)
CD27(P−value: 0.589)
CXCL10(P−value: 0.143)
PGF(P−value: 0.832)
TNFRSF21(P−value: 0.628)
>24
12−24
3(W)/6(M)−12
0−3(W)/6(M)
0
8.5
9.0
9.5
10.0
Alc_Re_C
0
0−3(W)/6(M)
3(W)/6(M)−12
12−24
>24

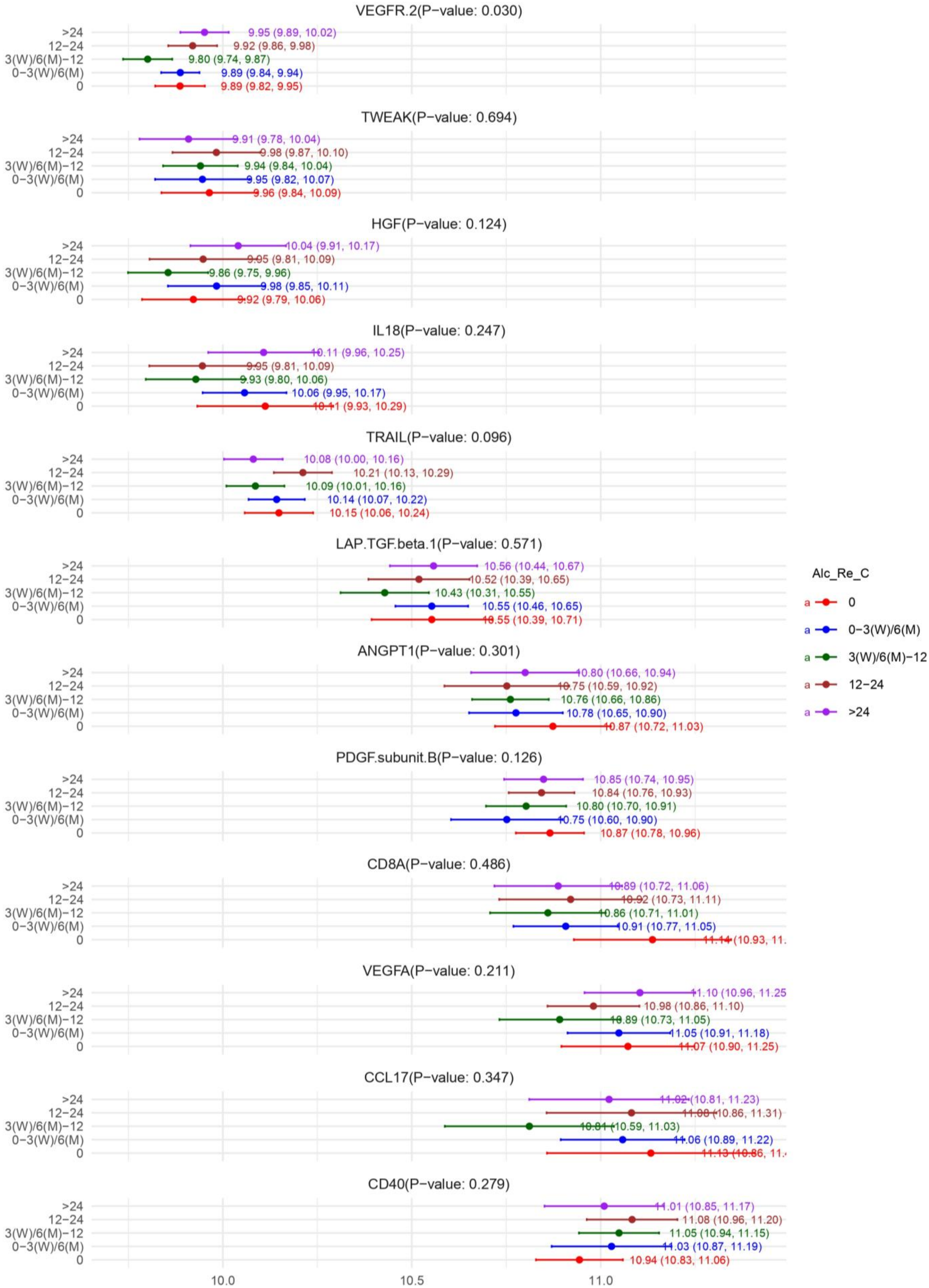

VEGFR.2(P−value: 0.030)
9.95 (9.89, 10.02)
9.92 (9.86, 9.98)
9.80 (9.74, 9.87)
9.89 (9.84, 9.94)
9.89 (9.82, 9.95)
TWEAK(P−value: 0.694)
9.91 (9.78, 10.04)
9.98 (9.87, 10.10)
9.94 (9.84, 10.04)
9.95 (9.82, 10.07)
9.96 (9.84, 10.09)
HGF(P−value: 0.124)
10.04 (9.91, 10.17)
9.95 (9.81, 10.09)
9.86 (9.75, 9.96)
9.98 (9.85, 10.11)
9.92 (9.79, 10.06)
IL18(P−value: 0.247)
10.11 (9.96, 10.25)
9.95 (9.81, 10.09)
9.93 (9.80, 10.06)
10.06 (9.95, 10.17)
10.11 (9.93, 10.29)
TRAIL(P−value: 0.096)
10.08 (10.00, 10.16)
10.21 (10.13, 10.29)
10.09 (10.01, 10.16)
10.14 (10.07, 10.22)
10.15 (10.06, 10.24)
LAP.TGF.beta.1(P−value: 0.571)
10.56 (10.44, 10.67)
10.52 (10.39, 10.65)
10.43 (10.31, 10.55)
10.55 (10.46, 10.65)
10.55 (10.39, 10.71)
ANGPT1(P−value: 0.301)
10.80 (10.66, 10.94)
10.75 (10.59, 10.92)
10.76 (10.66, 10.86)
10.78 (10.65, 10.90)
10.87 (10.72, 11.03)
PDGF.subunit.B(P−value: 0.126)
10.85 (10.74, 10.95)
10.84 (10.76, 10.93)
10.80 (10.70, 10.91)
10.75 (10.60, 10.90)
10.87 (10.78, 10.96)
CD8A(P−value: 0.486)
10.89 (10.72, 11.06)
10.92 (10.73, 11.11)
10.86 (10.71, 11.01)
10.91 (10.77, 11.05)
11.14 (10.93, 11.
VEGFA(P−value: 0.211)
11.10 (10.96, 11.25
10.98 (10.86, 11.10)
10.89 (10.73, 11.05)
11.05 (10.91, 11.18)
11.07 (10.90, 11.25)
CCL17(P−value: 0.347)
11.02 (10.81, 11.23)
11.08 (10.86, 11.31)
10.81 (10.59, 11.03)
11.06 (10.89, 11.22)
11.13 (10.86, 11.
CD40(P−value: 0.279)
11.01 (10.85, 11.17)
11.08 (10.96, 11.20)
11.05 (10.94, 11.15)
11.03 (10.87, 11.19)
10.94 (10.83, 11.06)
>24
12−24
3(W)/6(M)−12
0−3(W)/6(M)
0
10.0
10.5
11.0
Alc_Re_C
0
0−3(W)/6(M)
3(W)/6(M)−12
12−24
>24

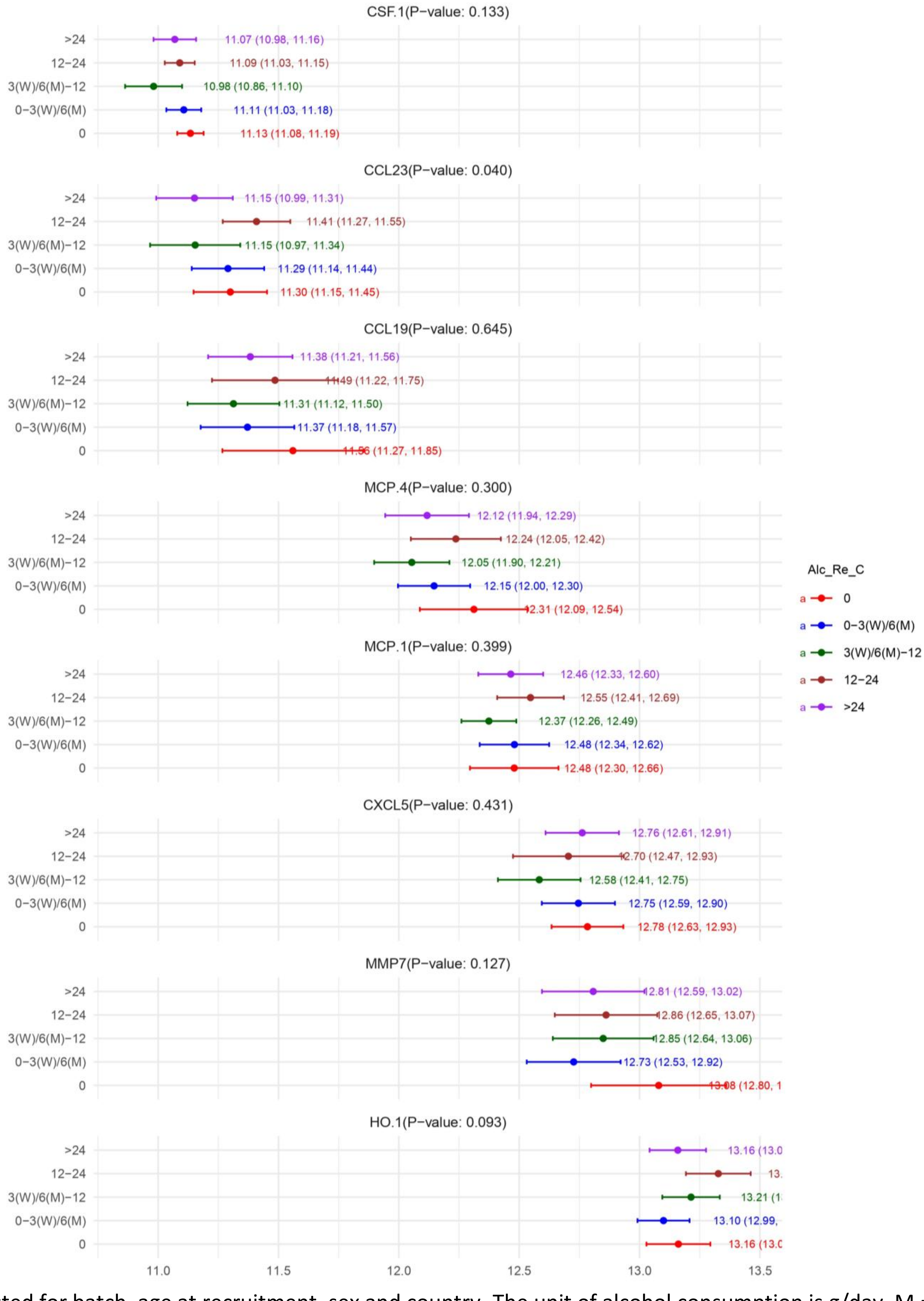

*Adjusted for batch, age at recruitment, sex and country. The unit of alcohol consumption is g/day, M = men, and W = women. The p-value was obtained using the Mann-Whitney U test because the 92 biomarkers do not follow a normal distribution.

Figure S10: Mean and 95% CI of Biomarkers by Self-reported Diabetes Status, among controls *

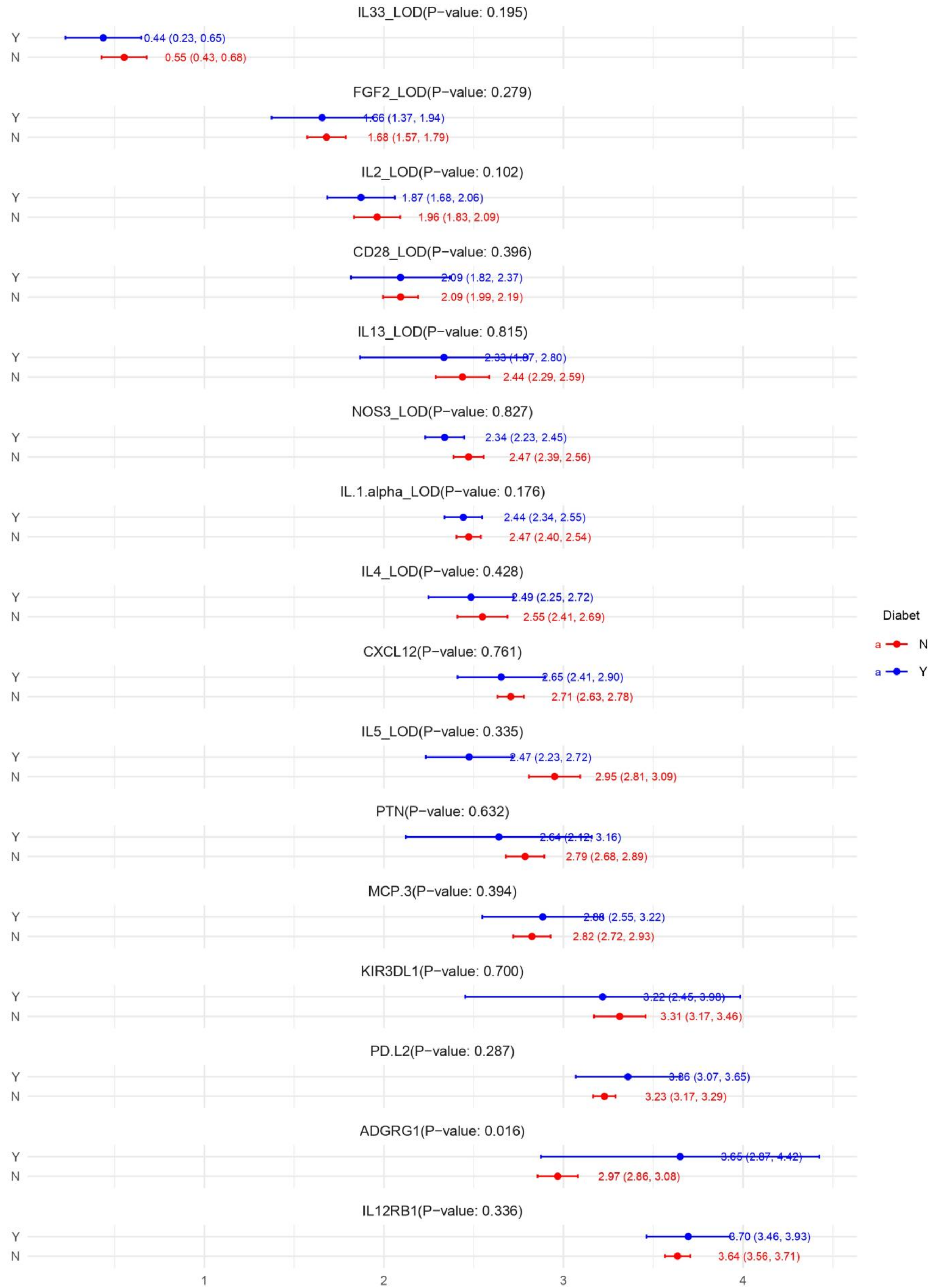

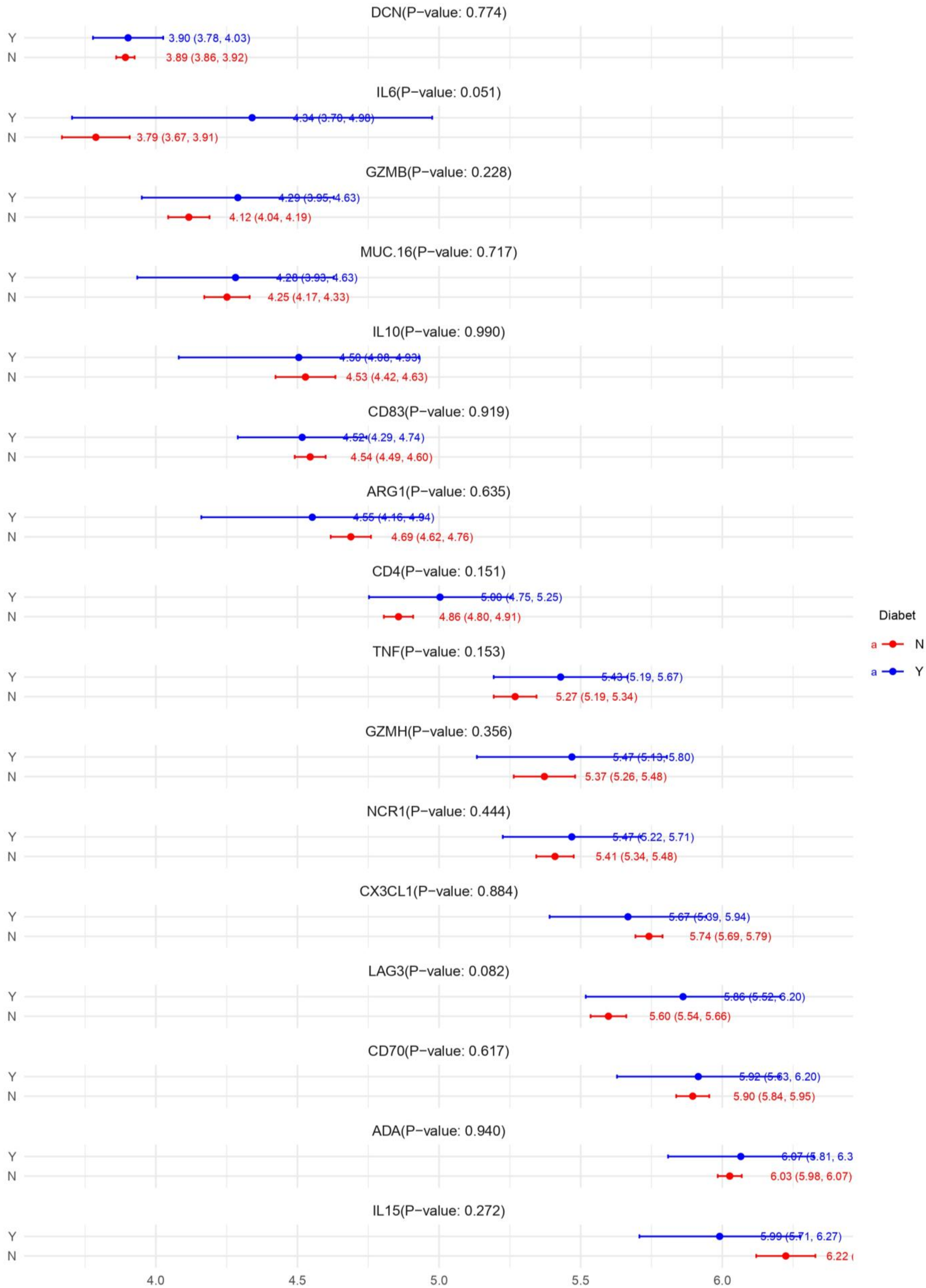
DCN(P−value: 0.774)
3.90 (3.78, 4.03)
3.89 (3.86, 3.92)
IL6(P−value: 0.051)
4.34 (3.70, 4.98)
3.79 (3.67, 3.91)
GZMB(P−value: 0.228)
4.29 (3.95, 4.63)
4.12 (4.04, 4.19)
MUC.16(P−value: 0.717)
4.28 (3.93, 4.63)
4.25 (4.17, 4.33)
IL10(P−value: 0.990)
4.50 (4.08, 4.93)
4.53 (4.42, 4.63)
CD83(P−value: 0.919)
4.52 (4.29, 4.74)
4.54 (4.49, 4.60)
ARG1(P−value: 0.635)
4.55 (4.16, 4.94)
4.69 (4.62, 4.76)
CD4(P−value: 0.151)
5.00 (4.75, 5.25)
4.86 (4.80, 4.91)
TNF(P−value: 0.153)
5.43 (5.19, 5.67)
5.27 (5.19, 5.34)
GZMH(P−value: 0.356)
5.47 (5.13, 5.80)
5.37 (5.26, 5.48)
NCR1(P−value: 0.444)
5.47 (5.22, 5.71)
5.41 (5.34, 5.48)
CX3CL1(P−value: 0.884)
5.67 (5.39, 5.94)
5.74 (5.69, 5.79)
LAG3(P−value: 0.082)
5.86 (5.52, 6.20)
5.60 (5.54, 5.66)
CD70(P−value: 0.617)
5.92 (5.63, 6.20)
5.90 (5.84, 5.95)
ADA(P−value: 0.940)
6.07 (5.81, 6.3
6.03 (5.98, 6.07)
IL15(P−value: 0.272)
5.99 (5.71, 6.27)
6.22 (
Y
N
4.0
4.5
5.0
5.5
6.0
Diabet
N
Y

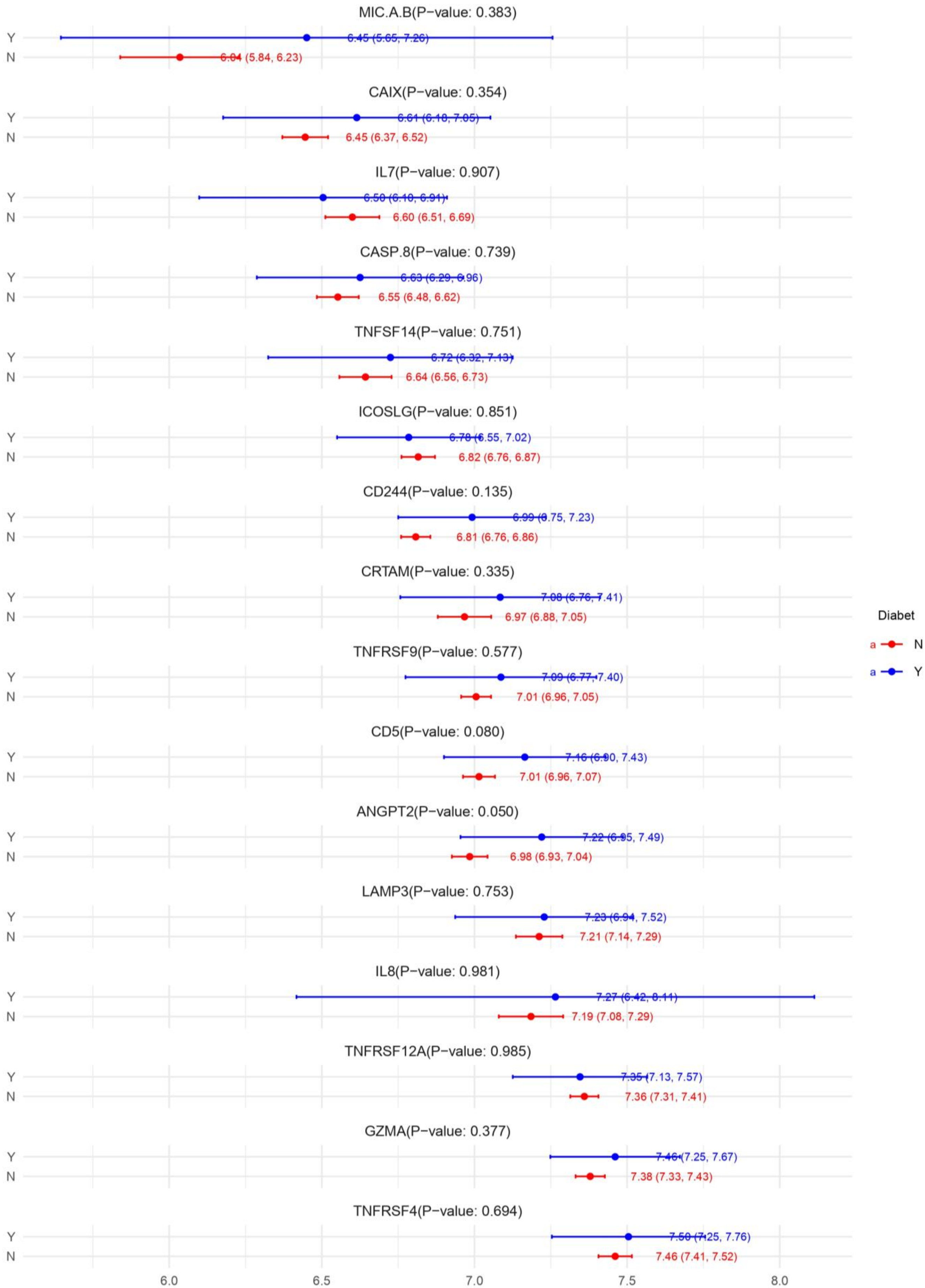

MIC.A.B(P−value: 0.383)
Y 6.45 (5.65, 7.26)
N 6.04 (5.84, 6.23)
CAIX(P−value: 0.354)
Y 6.61 (6.18, 7.05)
N 6.45 (6.37, 6.52)
IL7(P−value: 0.907)
Y 6.50 (6.10, 6.91)
N 6.60 (6.51, 6.69)
CASP.8(P−value: 0.739)
Y 6.63 (6.29, 6.96)
N 6.55 (6.48, 6.62)
TNFSF14(P−value: 0.751)
Y 6.72 (6.32, 7.13)
N 6.64 (6.56, 6.73)
ICOSLG(P−value: 0.851)
Y 6.78 (6.55, 7.02)
N 6.82 (6.76, 6.87)
CD244(P−value: 0.135)
Y 6.99 (6.75, 7.23)
N 6.81 (6.76, 6.86)
CRTAM(P−value: 0.335)
Y 7.08 (6.76, 7.41)
N 6.97 (6.88, 7.05)
TNFRSF9(P−value: 0.577)
Y 7.09 (6.77, 7.40)
N 7.01 (6.96, 7.05)
CD5(P−value: 0.080)
Y 7.16 (6.90, 7.43)
N 7.01 (6.96, 7.07)
ANGPT2(P−value: 0.050)
Y 7.22 (6.95, 7.49)
N 6.98 (6.93, 7.04)
LAMP3(P−value: 0.753)
Y 7.23 (6.94, 7.52)
N 7.21 (7.14, 7.29)
IL8(P−value: 0.981)
Y 7.27 (6.42, 8.11)
N 7.19 (7.08, 7.29)
TNFRSF12A(P−value: 0.985)
Y 7.35 (7.13, 7.57)
N 7.36 (7.31, 7.41)
GZMA(P−value: 0.377)
Y 7.46 (7.25, 7.67)
N 7.38 (7.33, 7.43)
TNFRSF4(P−value: 0.694)
Y 7.50 (7.25, 7.76)
N 7.46 (7.41, 7.52)
6.0
6.5
7.0
7.5
8.0
Diabet
N
Y

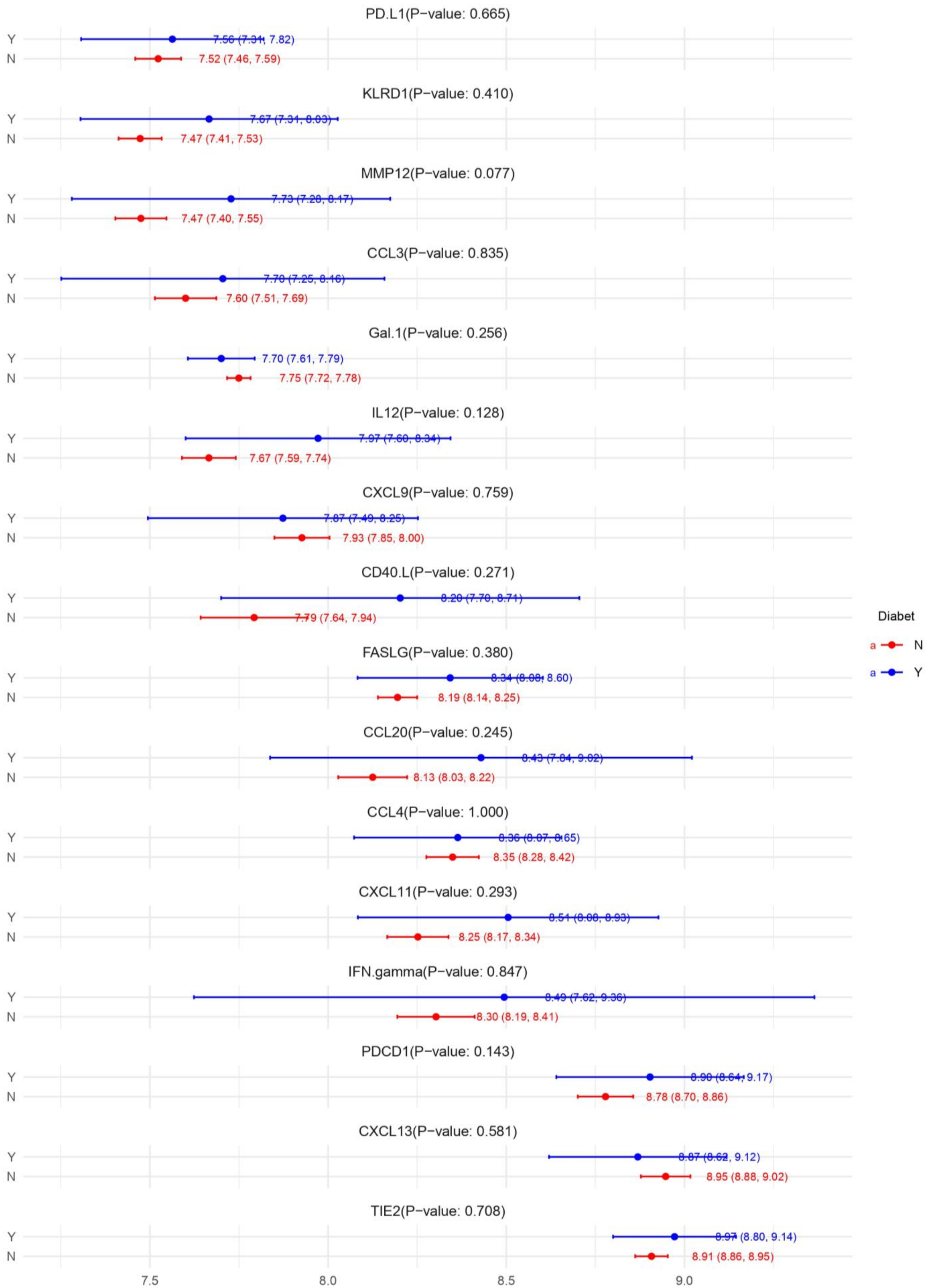

PD.L1(P−value: 0.665)
Y
N
7.56 (7.31, 7.82)
7.52 (7.46, 7.59)
KLRD1(P−value: 0.410)
7.67 (7.31, 8.03)
7.47 (7.41, 7.53)
MMP12(P−value: 0.077)
7.73 (7.28, 8.17)
7.47 (7.40, 7.55)
CCL3(P−value: 0.835)
7.70 (7.25, 8.16)
7.60 (7.51, 7.69)
Gal.1(P−value: 0.256)
7.70 (7.61, 7.79)
7.75 (7.72, 7.78)
IL12(P−value: 0.128)
7.97 (7.60, 8.34)
7.67 (7.59, 7.74)
CXCL9(P−value: 0.759)
7.87 (7.49, 8.25)
7.93 (7.85, 8.00)
CD40.L(P−value: 0.271)
8.20 (7.70, 8.71)
7.79 (7.64, 7.94)
FASLG(P−value: 0.380)
8.34 (8.08, 8.60)
8.19 (8.14, 8.25)
CCL20(P−value: 0.245)
8.43 (7.84, 9.02)
8.13 (8.03, 8.22)
CCL4(P−value: 1.000)
8.36 (8.07, 8.65)
8.35 (8.28, 8.42)
CXCL11(P−value: 0.293)
8.51 (8.08, 8.93)
8.25 (8.17, 8.34)
IFN.gamma(P−value: 0.847)
8.49 (7.62, 9.36)
8.30 (8.19, 8.41)
PDCD1(P−value: 0.143)
8.90 (8.64, 9.17)
8.78 (8.70, 8.86)
CXCL13(P−value: 0.581)
8.87 (8.62, 9.12)
8.95 (8.88, 9.02)
TIE2(P−value: 0.708)
8.97 (8.80, 9.14)
8.91 (8.86, 8.95)
7.5
8.0
8.5
9.0
Diabet
N
Y

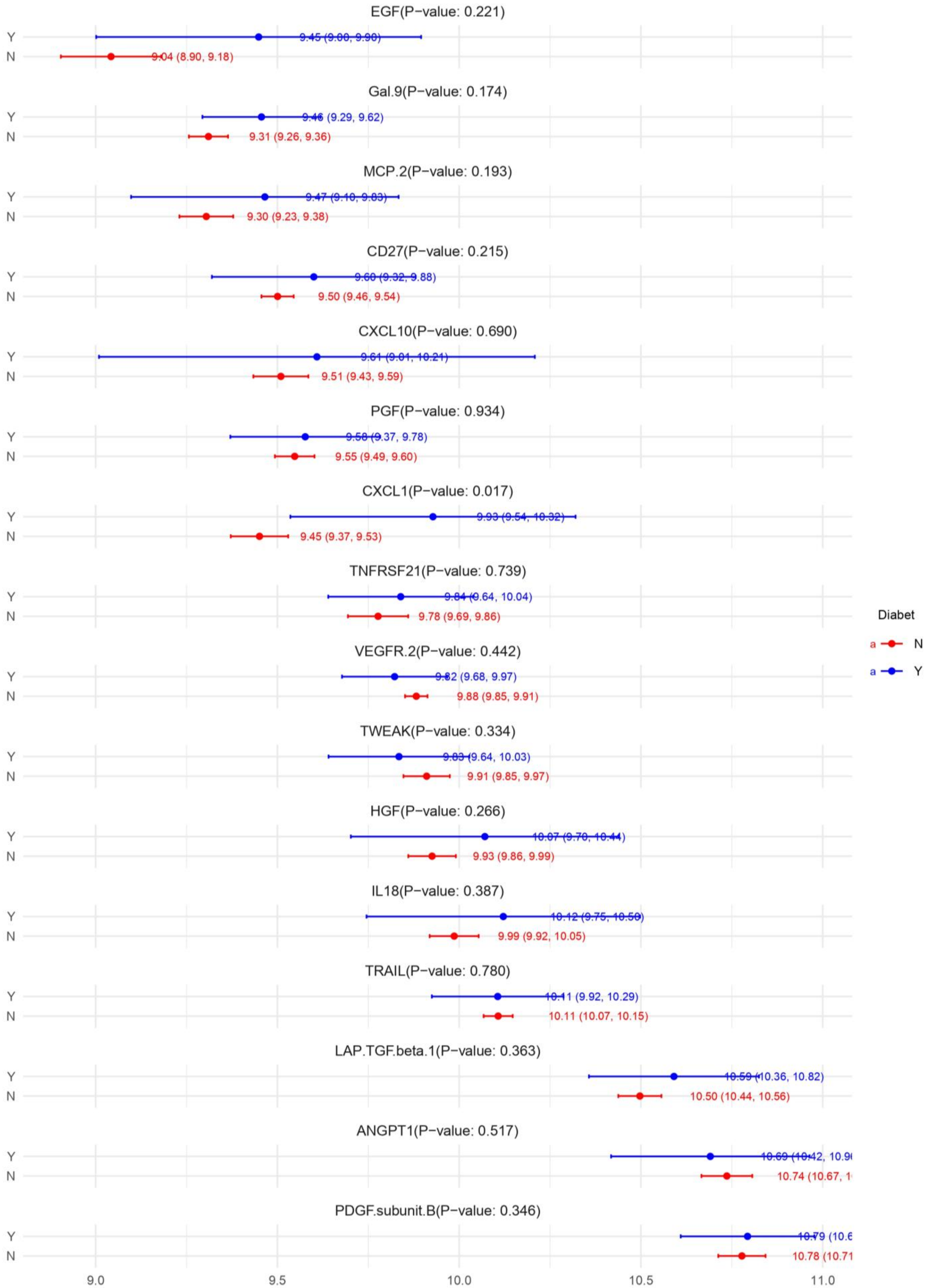
EGF(P−value: 0.221)
Y
N
9.45 (9.00, 9.90)
9.04 (8.90, 9.18)
Gal.9(P−value: 0.174)
9.46 (9.29, 9.62)
9.31 (9.26, 9.36)
MCP.2(P−value: 0.193)
9.47 (9.10, 9.83)
9.30 (9.23, 9.38)
CD27(P−value: 0.215)
9.60 (9.32, 9.88)
9.50 (9.46, 9.54)
CXCL10(P−value: 0.690)
9.61 (9.01, 10.21)
9.51 (9.43, 9.59)
PGF(P−value: 0.934)
9.58 (9.37, 9.78)
9.55 (9.49, 9.60)
CXCL1(P−value: 0.017)
9.93 (9.54, 10.32)
9.45 (9.37, 9.53)
TNFRSF21(P−value: 0.739)
9.84 (9.64, 10.04)
9.78 (9.69, 9.86)
VEGFR.2(P−value: 0.442)
9.82 (9.68, 9.97)
9.88 (9.85, 9.91)
TWEAK(P−value: 0.334)
9.83 (9.64, 10.03)
9.91 (9.85, 9.97)
HGF(P−value: 0.266)
10.07 (9.70, 10.44)
9.93 (9.86, 9.99)
IL18(P−value: 0.387)
10.12 (9.75, 10.50)
9.99 (9.92, 10.05)
TRAIL(P−value: 0.780)
10.11 (9.92, 10.29)
10.11 (10.07, 10.15)
LAP.TGF.beta.1(P−value: 0.363)
10.59 (10.36, 10.82)
10.50 (10.44, 10.56)
ANGPT1(P−value: 0.517)
10.69 (10.42, 10.9
10.74 (10.67, 1
PDGF.subunit.B(P−value: 0.346)
10.79 (10.6
10.78 (10.71
9.0
9.5
10.0
10.5
11.0
Diabet
N
Y

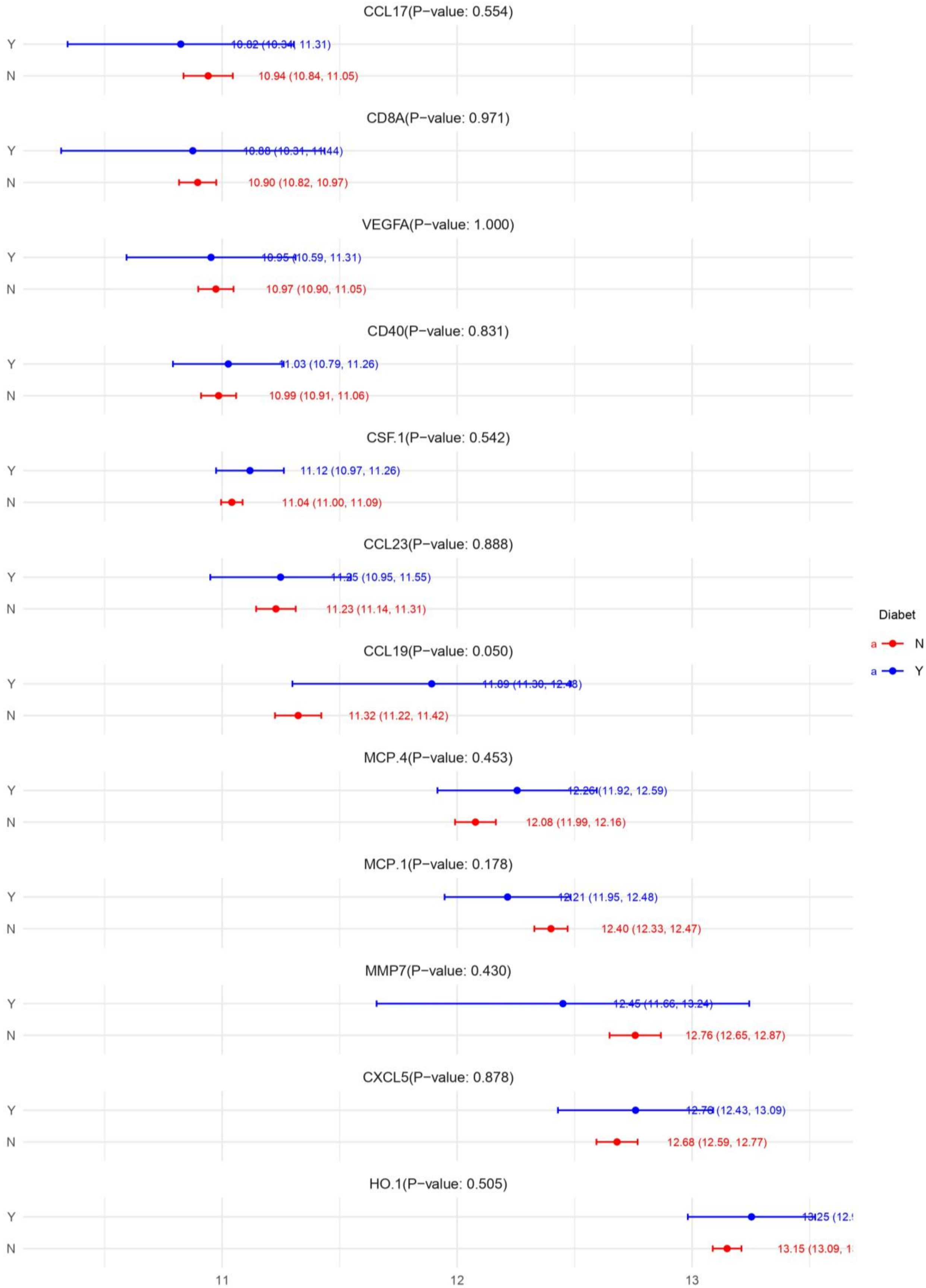

*Adjusted for batch, age at recruitment, sex and country. After excluding samples with unknown diabetes status, 720 participants remain in this analysis. Y = diabetes yes, N = diabetes no. The p-value was obtained using the Mann-Whitney U test because the 92 biomarkers do not follow a normal distribution.

Figure S11: Spearman partial correlation coefficients between the 8 significant biomarkers, among controls*

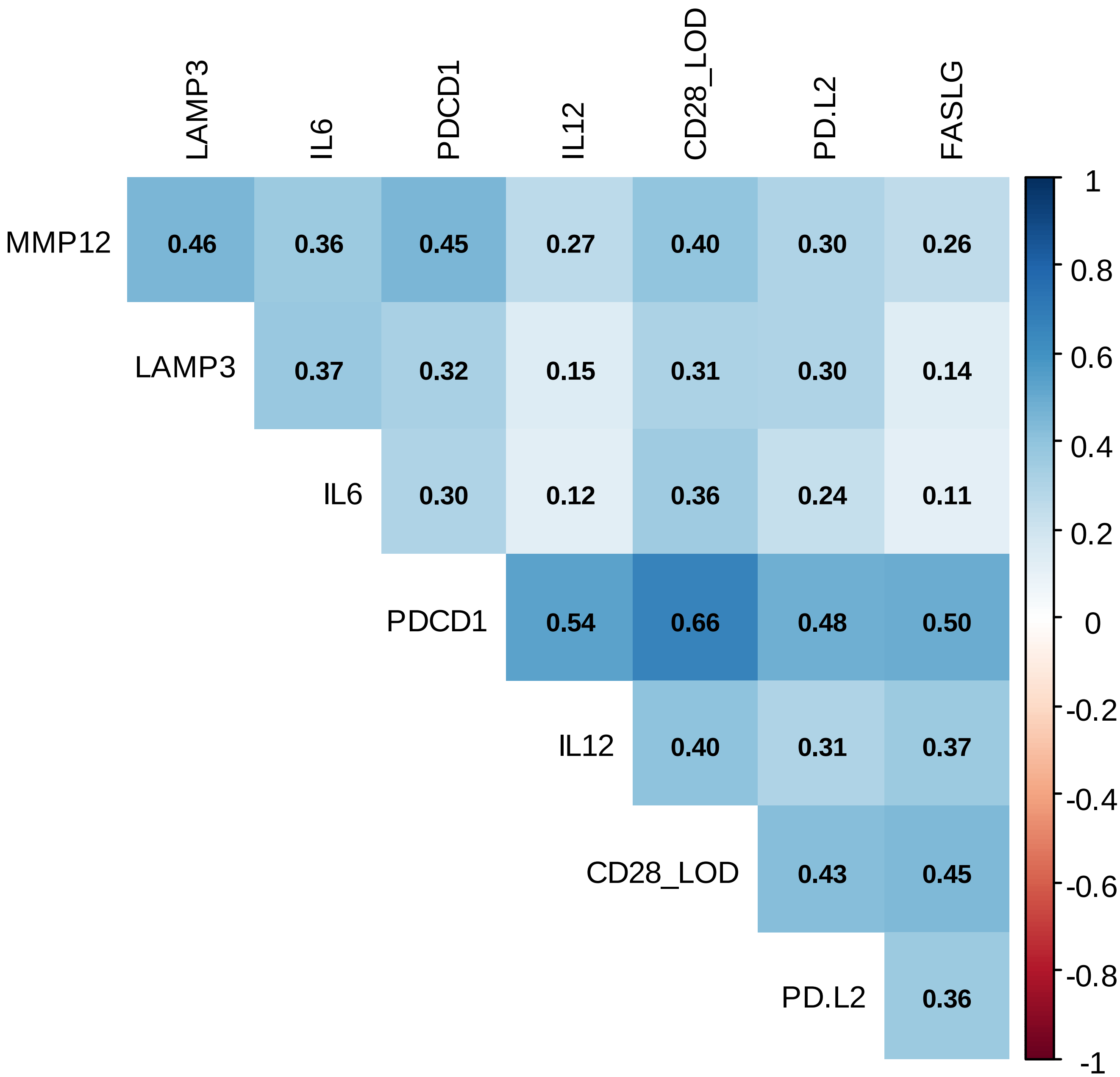

*Adjusted for batch, age at recruitment, sex and country
